# Safety and tolerability of weekly docetaxel plus nintedanib: A phase I trial after first-line chemotherapy failure in NSCLC

**DOI:** 10.1371/journal.pone.0292307

**Published:** 2023-10-17

**Authors:** Virginie Westeel, Wolfgang Schuette, Thierry Urban, Dejan Radonjic, Ute von Wangenheim, Robert M. Lorence, Martin Reck

**Affiliations:** 1 Service de Pneumologie, CHRU Besançon-Hôpital Minjoz, Besançon, France; 2 INSERM UMR1098, Université de Bourgogne Franche Comté, Besançon, France; 3 2^nd^ Medical Department, Krankenhaus Martha-Maria Halle-Dölau, Halle/Salle, Germany; 4 Service de Pneumologie, CHU Angers, Angers, France; 5 TA Oncology Medicine, Boehringer Ingelheim, Ingelheim am Rhein, Germany; 6 Department Global Biostatistics and Data Sciences, Boehringer Ingelheim, Biberach an der Riß, Germany; 7 Department of Medical Oncology, Boehringer Ingelheim, Ridgefield, CT, United States of America; 8 Department of Thoracic Oncology, Airway Research Center North, German Center for Lung Research, LungenClinic, Grosshansdorf, Germany; University College London Hospitals NHS Foundation Trust, UNITED KINGDOM

## Abstract

**Introduction:**

Studies have shown improved tolerability with once-weekly versus three-weekly docetaxel in the second-line treatment of advanced non-small cell lung cancer (NSCLC). This study aimed to evaluate the tolerability of nintedanib plus weekly docetaxel in patients with NSCLC.

**Methods:**

This phase I, open-label, dose-escalation study (NCT02668393) enrolled patients with locally advanced/metastatic adenocarcinoma NSCLC that had progressed on first-line platinum chemotherapy. The primary endpoint was to determine the maximum tolerated dose of nintedanib (up to 200 mg twice daily [BID]) combined with weekly docetaxel (35 mg/m^2^) on days 1, 8, and 15 based on the occurrence of dose-limiting toxicities (DLTs) over a 28-day treatment cycle. Adverse events (AEs) were also evaluated.

**Results:**

The trial terminated prematurely due to recruitment challenges. At termination, seven patients had received nintedanib 150 mg BID and seven nintedanib 200 mg BID, in combination with weekly docetaxel. In the first treatment cycle, DLTs were reported for 1/6 evaluable patients (16.7%) in each group. The disease control rates were 57.1% and 42.9%, respectively. Grade ≥3 treatment-related AEs affected three patients in each group (42.9%); neutropenia was reported in one patient (14.3%) in each group. Treatment-related serious AEs were reported in three patients (42.9%) receiving nintedanib 150 mg, and two patients (28.6%) receiving nintedanib 200 mg.

**Conclusions:**

Overall, nintedanib plus weekly docetaxel was well-tolerated in patients with locally advanced or metastatic lung adenocarcinoma who progressed on first-line platinum-based chemotherapy, without loss of efficacy. DLTs were manageable.

## Introduction

Although the treatment landscape for advanced non-small cell lung cancer (NSCLC) has undergone recent advances, there remains an unmet need for well-tolerated and clinically effective treatments for patients who progress after first-line immunochemotherapy. The known interrelationship between pro-angiogenic factors and immune evasion provides a strong rationale for the use of anti-angiogenic treatments in patients with NSCLC in this setting [1]. The angiokinase inhibitor, nintedanib, is approved in the European Union and other countries for use in combination with docetaxel for the treatment of locally advanced metastatic or locally recurrent NSCLC of adenocarcinoma histology after failure of first-line chemotherapy [2]. Furthermore, several recent observational trials have indicated that the combination is clinically active in patients with NSCLC following immunochemotherapy or immunotherapy [3–5].

In the phase III registration trial, LUME-Lung 1, nintedanib (200 mg twice daily [BID]) plus docetaxel (75 mg/m^2^ once every 3 weeks) significantly increased survival compared with docetaxel alone in patients with lung adenocarcinoma who progressed following first-line platinum-based chemotherapy [6]. However, the administration of docetaxel 75 mg/m^2^ on a three-weekly dosing schedule has been associated with a high frequency of severe myelosuppression, including neutropenia and leukopenia, resulting in more infection-related adverse events (AEs) such as sepsis [6, 7]. In an effort to mitigate these AEs, docetaxel in a weekly dosing regimen has been investigated. A meta-analysis of five trials with a combined enrolment of 865 patients showed that weekly docetaxel as second-line treatment of advanced NSCLC led to significantly lower incidence and severity of febrile neutropenia compared with three-weekly docetaxel, without loss of efficacy [7]. In the phase IIb SENECA trial, there was a tolerability benefit with once-weekly versus three-weekly docetaxel, with significantly lower incidences of overall afebrile neutropenia and grade ≥3 afebrile neutropenia, while median overall survival was comparable between regimens [7].

This phase I study aimed to build on the existing evidence by exploring the maximum tolerated dose (MTD) of nintedanib and its tolerability in combination with a weekly schedule of docetaxel in patients with advanced/metastatic NSCLC after failure of first-line therapy.

## Materials and methods

### Study design and patients

**Fig 1:**
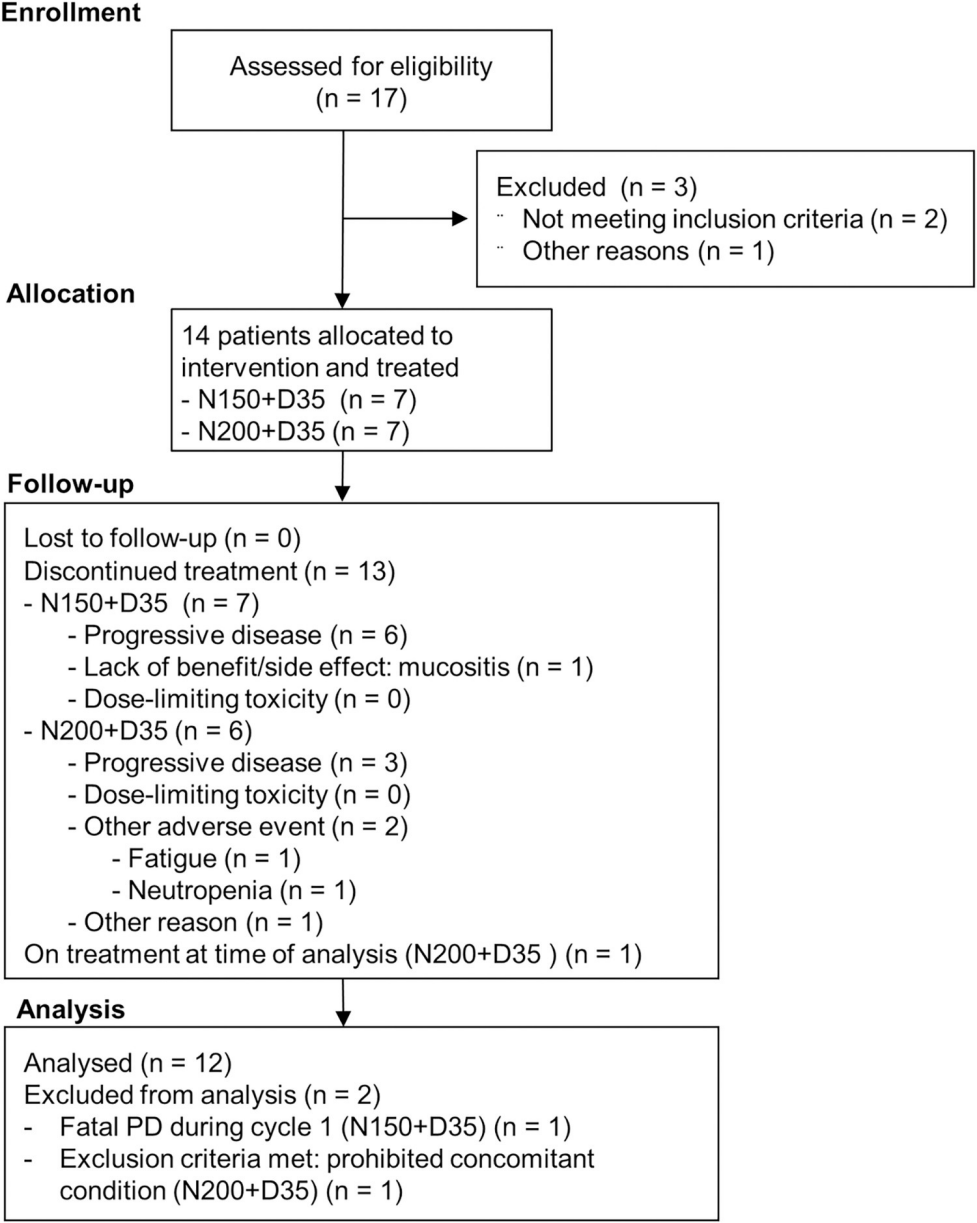
Participant flow.

This phase I, open-label study (NCT02668393) used a 3+3 dose-escalation design to explore the tolerability of second-line nintedanib plus weekly docetaxel in a 28-day cycle in patients with advanced/metastatic NSCLC. This study was conducted in four centers in France and Germany. Participant flow is shown in Fig 1. Data were collected by the investigator in an electronic case report form. The Independent Ethics Committees of the participating centers approved the protocol, and written informed consent was obtained from every participant. The study was carried out in accordance with Good Clinical Practice and the Declaration of Helsinki. The study protocol was approved by Le comité de protection des personnes Est II, CHU hôpital Saint Jacques, Besançon, France, and the Ethikkommissionen bei der Ärztekammer Schleswig-Holstein, Bad Segeberg, Germany.

The key criteria for eligibility were a diagnosis of histologically/cytologically confirmed, locally advanced, or metastatic (stage IIIB–IV) lung adenocarcinoma that had progressed on first-line platinum-based chemotherapy (which included continuation or switch maintenance therapy, one prior adjuvant and/or neoadjuvant chemotherapy line, and prior immunochemotherapy regimens). Other inclusion criteria were age ≥18 years at the date of informed consent, a life expectancy of ≥3 months, and an Eastern Cooperative Oncology Group (ECOG) performance status of ≤1 at screening. Patients were excluded if they had received ≥1 line of prior chemotherapy for advanced or metastatic NSCLC, tested positive for activating epidermal growth factor receptor (*EGFR*) mutations or anaplastic lymphoma kinase (*ALK*) translocations, or received previous therapy with other vascular endothelial growth factor (VEGF) or VEGF receptor inhibitors (other than bevacizumab). Patients were also ineligible if they had received other investigational drugs or received any of the following treatments within 4 weeks prior to the study start date: chemotherapy, hormone therapy, immunotherapy, antibody treatment or radiotherapy.

### Study treatments

**Fig 2:**
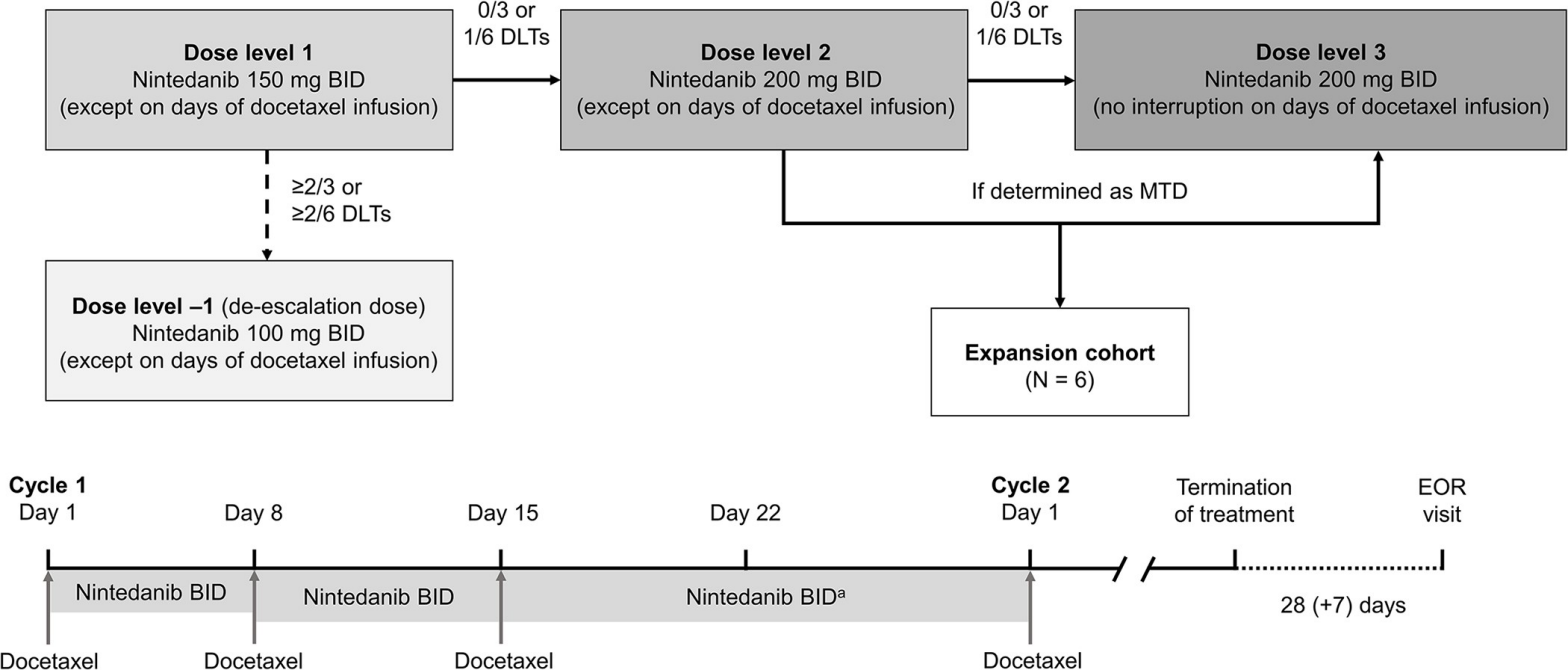
Study design. Shaded boxes represent treatment groups for which patients were recruited following a 3+3 design. Clear boxes represent additional treatment groups that did not follow a 3+3 design. ^a^2 weeks of rest for docetaxel administration after administration on Day 15.

Nintedanib was initiated at a dose of 150 mg orally BID, starting with the morning dose on the day after docetaxel administration days (Days 2–7, 9–14, and 16–28 in each 28-day cycle). The dose of nintedanib was increased to a maximum of 200 mg BID using a 3+3 dose escalation design (Fig 2). If the dose of nintedanib 200 mg BID was well-tolerated without dose-limiting toxicities (DLTs), it was administered continuously, without interruption on the days of docetaxel infusion. Docetaxel was administered at a fixed dose of 35 mg/m^2^ on Days 1, 8 and 15 of each 28-day cycle (intravenous infusion over 60 minutes). All patients received oral corticosteroids for 3 days, starting 1 day before docetaxel infusion. Treatment was continued until progressive disease (PD), intolerable toxicity or withdrawal of consent. The trial was considered completed for individual patients following the end of the residual effect period (28 + 7 days after discontinuation), loss to follow up, withdrawal of consent or death.

### Management of AEs

If AEs occurred and were deemed to be related to nintedanib by the investigator, treatment was interrupted to facilitate resolution. The dose of nintedanib was then restarted at the lower dose of either 150 mg BID or 100 mg BID. AEs that were deemed to be related to docetaxel were managed conservatively where possible between doses of docetaxel. Patients were permitted to receive treatment for symptomatic side effects such as a decreased neutrophil count, for which granulocyte colony-stimulating factor could be administered at the investigator’s discretion. Treatment was discontinued in patients who had PD, significant protocol deviations, or were unable to complete docetaxel infusions for reasons such as infusion-related hypersensitivity. Patients who experienced radiological progression according to the Response Evaluation Criteria in Solid Tumours (RECIST) version 1.1 but were judged by the investigator to have derived clinical benefit could remain in the study if appropriate following discussion with the sponsor.

### Study outcomes

The primary objective was to determine the MTD of nintedanib, and this was based on the occurrence of DLTs during the first treatment cycle. The MTD was defined as the highest dose combination studied for which the incidence of DLTs was no more than 16.7% (n = 1/6) during the first treatment cycle. Patients who did not complete the first treatment cycle for reasons other than DLT were to be excluded from the MTD evaluation set used in the primary endpoint analysis. The objective response (OR) in all patients treated (treated set, TS), duration of response, and progression-free survival (PFS) were evaluated as exploratory efficacy endpoints. OR was confirmed by imaging 4 weeks or later after the first occurrence of the response. Response was assessed according to RECIST version 1.1.

Safety was evaluated in the TS based on the occurrence of AEs, which were defined according to the Common Terminology Criteria for Adverse Events version 4.0 and the Medical Dictionary for Regulatory Activities version 22.0, and AEs relating to DLTs. AEs that started before the first drug intake and deteriorated during treatment were also evaluated as treatment-emergent AEs (TEAEs). A serious AE (SAE) was defined as any AE that resulted in death or hospitalization or presented a significant hazard to the patient.

### Statistical analysis

This trial was exploratory and aimed to enroll a fixed sample of 30 patients without formal calculation of sample size. The statistical evaluation set for determining the MTD consisted of all patients who received at least one dose of the study medication and were not replaced. Efficacy and safety endpoints were determined using descriptive exploratory analyses performed on the treated analysis set, which consisted of all patients who received at least one dose of study medication. OS and PFS were assessed based on Kaplan–Meier estimates, point estimates and confidence intervals (CIs). All analyses were performed using the software SAS^®^ (version 9.4 or higher) [8].

## Results

### Patient characteristics

The trial was terminated prematurely due to difficulty enrolling the target number of patients. Between 6 June 2016 and 27 November 2019, a total of seven patients received nintedanib 150 mg BID plus docetaxel, and seven patients received nintedanib 200 mg BID plus docetaxel (TS, Fig 1). At the time of primary analysis, 13 patients had discontinued combination treatment and one remained on treatment. The most common reason for permanent discontinuation was PD. None of the patients discontinued due to DLT. No patients were lost to follow up.

The baseline characteristics of the patients were similar in both treatment groups (Table 1). All patients for whom race was known were white (71.4%, five in each group). The median age was 65.0 years in the nintedanib 150 mg BID group and 63.0 years in the 200 mg BID group. All patients were either ex or current smokers. All patients had baseline comorbidities, most commonly respiratory disorders, which affected 4/7 patients (57.1%) in each group.

**Table 1:** Patient demographics and disease characteristics.

		Nintedanib 150 mg BID + docetaxel (n = 7)	Nintedanib 200 mg BID + docetaxel (n = 7)	Total (n = 14)
**Median age, years (range)**		65.0 (58.0–77.0)	63.0 (48.0–71.0)	63.0 (48.0–77.0)
Sex, n (%)	Male	5 (71.4)	5 (71.4)	10 (71.4)
	Female	2 (28.6)	2 (28.6)	4 (28.6)
Race, n (%)	White	5 (71.4)	5 (71.4)	10 (71.4)
	Missing^a^	2 (28.6)	2 (28.6)	24 (28.6)
**Median BMI, kg/m**^**2**^ **(range)**		26.12 (23.4–27.9)	26.68 (21.2–33.5)	26.18 (21.2–33.5)
Smoking status, n (%)	Never smoked	0	0	0
	Ex-smoker	7 (100.0)	6 (85.7)	13 (92.9)
	Current smoker	0	1 (14.3)	1 (7.1)
ECOG PS at baseline, n (%)	0	3 (42.9)	3 (42.9)	6 (42.9)
	1	4 (57.1)	4 (57.1)	8 (57.1)
**Months since diagnosis, median (range)**		8.48 (3.9–28.9)	9.53 (4.7–37.1)	9.13 (3.9–37.1)
Clinical stage at diagnosis (UICC/AJCC), n (%)	<4	3 (42.9)	1 (14.3)	4 (28.6)
	4	4 (57.1)	6 (85.7)	10 (71.4)
Histological classification, n (%)	Adenocarcinoma	7 (100.0)	6 (85.7)	13 (92.9)
	Other^b^	0	1 (14.3)	1 (7.1)
Tumor clinical stage at screening, n (%)	Locally advanced	1 (14.3)	0	1 (7.1)
	Metastatic	6 (85.7)	7 (100.0)	13 (92.9)
*EGFR* mutation result, n (%)	Negative	7 (100.0)	5 (71.4)	12 (85.7)
	Unknown/missing	0	2 (28.6)	2 (14.2)
*ALK* mutation result, n (%)	Negative	7 (100.0)	5 (71.4)	12 (85.7)
	Unknown/missing	0	2 (28.6)	2 (14.2)
Previous treatment(s), n (%)	Radiotherapy	5 (71.4)	1 (14.3)	6 (42.9)
	Surgery	1 (14.3)	2 (28.6)	3 (21.4)
	Immunotherapy	0	1 (14.3)	1 (7.1)
	Adjuvant therapy	1 (14.3)	0	1 (7.1)
**Number of lines of previous systemic anticancer therapies, n (%)**	1	5 (71.4)	6 (85.7)	11 (78.6)
	2	2 (28.6)	1 (14.3)	3 (21.4)

BID, twice daily; BMI, body mass index; ECOG PS, Eastern Cooperative Oncology Group performance status; EGFR, epidermal growth factor receptor; UICC/AJCC, Union for International Cancer Control /American Joint Committee on Cancer.

^a^Race data was not collected in France

^b^One patient who was classified histologically as ‘other’ was considered to have adenocarcinoma based on cytology results.

All enrolled patients had lung cancer of adenocarcinoma histology, as required by the trial protocol, and all patients except one had metastatic disease (Table 1). The majority of patients had stage IV disease at diagnosis (four patients [57.1%] in the nintedanib 150 mg BID group, and six patients [85.7%] in the nintedanib 200 mg BID group). The results of *EGFR* and *ALK* mutation testing were both negative for all patients in the nintedanib 150 mg BID group, and 5/7 patients (71.4%) in the nintedanib 200 mg BID group (results were unknown or missing for two patients who received nintedanib 200 mg). All patients had received previous systemic anticancer therapy; one patient in the nintedanib 150 mg BID group had received previous adjuvant therapy (14.3%) and one patient in the 200 mg BID group had received previous nivolumab (14.3%). The proportion of patients who had received prior radiotherapy was higher in the 150 mg BID group than in the 200 mg BID group (five patients [71.4%] and one patient [14.3%], respectively).

### Primary endpoint

Of 14 patients treated, two were excluded from the MTD evaluation set. During the first treatment cycle, DLTs were reported for 1/6 patients (16.7%) in each group in the MTD analysis set (n = 12, Table 2). One patient in the nintedanib 150 mg BID group had intermittent treatment-related grade 3 diarrhea, which started on Day 4, with a total of three episodes across 4 days. One patient in the nintedanib 200 mg BID group had treatment-related grade 3 elevated alanine aminotransferase (ALT) for 7 days between Day 8 and Day 14 and resolved without the need for treatment. Both DLTs were non-serious and were managed with dose reductions to nintedanib 100 mg BID and 150 mg BID, respectively. In the treated analysis set, an additional DLT of treatment-related grade 3 hypertension was reported in the nintedanib 150 mg BID group, which had not resolved at the final study visit. This was considered non-serious and study treatment was continued. The MTD of nintedanib was not reached due to the premature termination of the trial, which precluded further dose escalation.

**Table 2:** Dose-limiting toxicities during the first treatment cycle in the MTD evaluation set.

		Nintedanib 150 mg BID + docetaxel (n = 6)			Nintedanib 200 mg BID + docetaxel (n = 6)		
		All grades n (%)	Grade 1/2 n (%)	Grade ≥3 n (%)	All grades n (%)	Grade 1/2 n (%)	Grade ≥3 n (%)
**Patients with events**		1 (16.7)	0	1 (16.7)	1 (16.7)	0	1 (16.7)
GI disorders	Diarrhea	1 (16.7)	0	1 (16.7)	0	0	0
Investigations	Elevated ALT	0	0	0	1 (16.7)	0	1 (16.7)

ALT, alanine aminotransferase; BID, twice daily; GI, gastrointestinal.

### Efficacy outcomes

**Fig 3:**
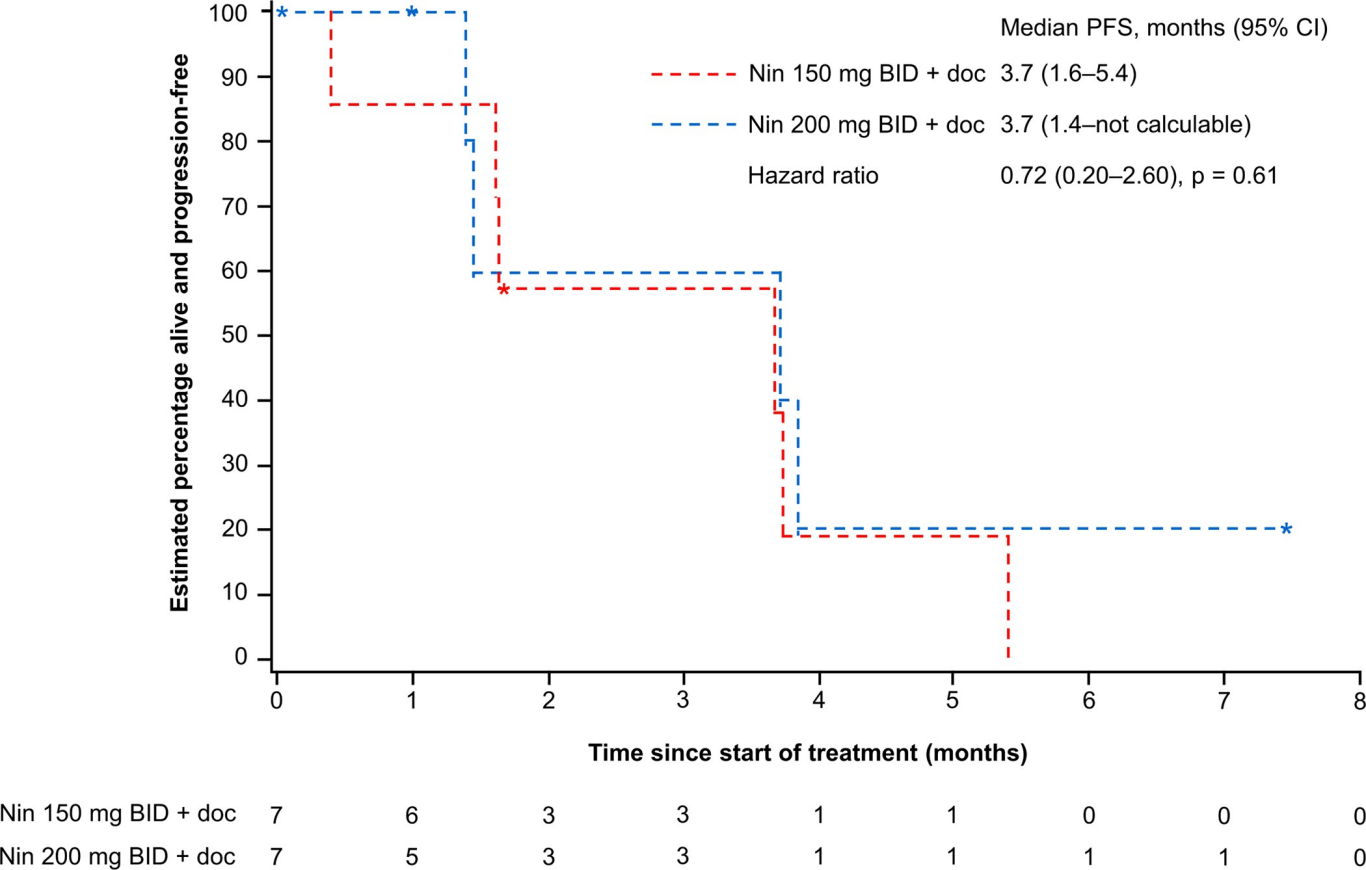
PFS in the treated set.

An OR (partial or complete response) was not achieved in any patient (Table 3). In the nintedanib 150 mg BID group, four patients (57.1%) had stable disease (SD) and two (28.6%) had PD. In the nintedanib 200 mg BID group, three patients (42.9%) had SD and one (14.3%) had PD. One of the three patients with SD was re-evaluated as having PD after primary database lock. Four patients were not evaluable for response due to missing data. At the time of termination, the median PFS was 3.7 months (95% CI: 1.6–5.4) in the nintedanib 150 mg BID group and 3.7 months (95% CI: 1.4–not determined) in the nintedanib 200 mg BID group (Fig 3).

**Table 3:** Best overall tumor response in patients who received at least one dose of study medication^a^.

	Nintedanib 150 mg + docetaxel (n = 7)	Nintedanib 200 mg + docetaxel (n = 7)
**CR**	0	0
**PR**	0	0
**SD**	4 (57.1)	3 (42.9)
**PD**	2 (28.6)	1 (14.3)
**ORR (CR + PR)**	0	0
**DCR (CR + PR + SD)**	4 (57.1)	3 (42.9)

CR, complete response; DCR, disease control rate; ORR, objective response rate; PD, progressive disease; PR, partial response; SD, stable disease.

^a^Best overall response data was missing for one patient in the nintedanib 150 mg treatment group, and three patients in the nintedanib 200 mg treatment group.

### Safety results

In the nintedanib 150 mg BID group, the median duration of exposure was 1.8 months (range: 0.3–5.5) for nintedanib and 1.4 months (range: 0.3–3.7) for docetaxel. In the nintedanib 200 mg BID group, the median exposure was 1.3 months (range: 0.2–9.9) for nintedanib and 1.2 months (range: 0.3–8.1) for docetaxel.

All patients in the nintedanib 150 mg BID group and 6/7 patients (85.7%) in the 200 mg BID group reported TEAEs (S1 Table). One patient in the nintedanib 200 mg BID group had a protocol-defined AE of special interest of elevated ALT. The most common all-grade TEAEs that affected ≥50% of patients were diarrhea (71.4%), cough (57.1%), and dyspnea (57.1%) in the nintedanib 150 mg BID group, and diarrhea (57.1%) in the nintedanib 200 mg BID group. Grade ≥3 TEAEs occurred in five patients in the nintedanib 150 mg BID group (71.4%) and four patients in the 200 mg BID group (57.1%). Investigator-defined treatment-related AEs (TRAEs) were reported in six patients (85.7%) receiving nintedanib 150 mg BID and five patients (71.4%) receiving nintedanib 200 mg BID. The incidence did not appear to be dose related. The only TRAEs to affect ≥50% of patients in each group were gastrointestinal disorders, most commonly diarrhea, which affected 71.4% of patients receiving nintedanib 150 mg BID and 57.1% of patients receiving nintedanib 200 mg BID. Grade ≥3 TRAEs occurred in three patients in each group (42.9%). AEs of treatment-related myelosuppression were generally uncommon. In the nintedanib 150 mg BID group, anemia was reported in three patients (42.9%), all of which were grade 1 or 2, and neutropenia and leukopenia were each reported in one patient (14.3%), both of which were grade ≥3. In the nintedanib 200 mg BID group, anemia and neutropenia were each reported in one patient (14.3%) and were of grade 1 or 2 severity; there were no reported cases of leukopenia in this group.

In addition to the dose reductions due to DLTs described above, the nintedanib dose was reduced for one patient receiving nintedanib 200 mg BID (to 150 mg) for grade 2 diarrhea, which started on Day 3 and was unresolved at the end of the study despite treatment. The same patient had a grade 2 rectal hemorrhage lasting 2 days that resolved without requiring an intervention. AEs required permanent discontinuation of either one or both trial drugs in one patient receiving nintedanib 150 mg BID and two patients receiving nintedanib 200 mg BID. In the nintedanib 150 mg BID group, discontinuation of both docetaxel and nintedanib was required in a patient experiencing grade 3 fatigue, which started on Day 8 and was unresolved at the end of treatment. This was considered non-serious and not related to the trial medication. In the nintedanib 200 mg BID group, docetaxel was discontinued in two patients. One patient experienced grade 3 fatigue, which started on day 40 and had not resolved by the final visit. The other patient experienced grade 1 neutropenia, which started on day 73 and lasted for 30 days before resolving. Both events were considered treatment-related but non-serious.

SAEs were reported in three patients (42.9%) in the nintedanib 150 mg BID group, and two patients (28.6%) in the nintedanib 200 mg BID group, all of which required hospitalization. SAEs in the nintedanib 150 mg BID group were grade 3 nausea, dyspnea, and cough (cough was concomitant with dyspnea in one patient). SAEs in the nintedanib 200 mg BID group were grade 3 general physical health deterioration, and grade 2 empyema. None were considered treatment related. Two deaths occurred, which included a 60-year-old male in the nintedanib 150 mg BID group and a 66-year-old male in the nintedanib 200 mg BID group. Both deaths were related to disease progression, and neither was considered to be treatment related.

## Discussion

This open-label, phase I trial builds on the existing evidence supporting the use of weekly docetaxel. The trial was terminated prematurely due to recruitment challenges relating to the approval of immunotherapies as a second-line treatment option in NSCLC after the trial was initiated [9]. Although the MTD of nintedanib was not reached, the results suggest that nintedanib doses of 150 mg BID and 200 mg BID plus weekly docetaxel are well tolerated; AEs were manageable, and there were no unexpected safety signals. The disease control rate (DCR) and median PFS with nintedanib 200 mg BID in this study (42.9% months and 3.7 months, respectively) were similar to the DCR and PFS reported in the LUME-Lung 1 study (38.5% and 4.2 months, respectively) [6], suggesting that the use of weekly docetaxel is not associated with inferior efficacy.

Our study adds to the existing evidence by indicating that nintedanib plus weekly docetaxel has a manageable safety profile [7], and that the occurrence of neutropenia is not necessarily treatment-limiting. In LUME-Lung 1, grade ≥3 neutropenia affected 12.1% of patients receiving nintedanib plus docetaxel [6]. In this study, there were no reported cases of grade ≥3 neutropenia in the nintedanib 200 mg group, and one case (14.3%) in the nintedanib 150 mg group. Despite the small sample, the results suggest that nintedanib plus weekly docetaxel may reduce the severity of cytotoxic AEs pertaining to myelosuppression. DLTs affected one patient (16.7%) in each group and were manageable. The overall pattern of AEs observed in this study did not appear to increase with the higher dose of nintedanib 200 mg. Overall, the safety results suggest that the combination of nintedanib with weekly docetaxel has a manageable safety profile.

This study builds on the findings of the phase IIb SENECA trial of nintedanib after first-line chemotherapy in NSCLC. The SENECA trial evaluated nintedanib 200 mg BID in combination with a reduced dose of docetaxel (33 mg/m^2^) administered on Day 1 and Day 8 of a 21-day cycle versus standard three-weekly dosing [10]. At a median follow-up of 35.5 months, there was a lower incidence of toxicities with weekly docetaxel compared with three-weekly docetaxel, including afebrile neutropenia (2.5% vs 10.4%, p < 0.0001), with no significant difference in PFS (4.8 months for both dosing groups, p = 0.8) or OS (8.5 vs 9.6 months, p = 0.3) [10]. The SENECA trial concluded that weekly docetaxel plus nintedanib has comparable efficacy to three-weekly docetaxel in combination with nintedanib in the second-line treatment of NSCLC but with reduced toxicity [10]. The median PFS in our study (3.7 months in both dose groups) is similar to the results of the SENECA trial. The similarity in PFS between dosing groups in our study also suggests that weekly docetaxel has similar efficacy in combination with either nintedanib 200 mg BID or 150 mg BID. The main limitation of this study was the premature termination and the resultant low number of participants enrolled. This precluded formal hypothesis testing, subgroup analyses, and testing for interactions between age and treatment effect.

In conclusion, this study provides further evidence that the combination of nintedanib plus weekly docetaxel is well tolerated in patients with locally advanced or metastatic lung adenocarcinoma who progressed on first-line platinum-based chemotherapy, with no apparent loss of docetaxel efficacy. Since the initiation of this trial, immunochemotherapy (or immunotherapy alone) has replaced chemotherapy as standard first-line treatment for patients with NSCLC lacking actionable mutations, based on seminal trials such as KEYNOTE 189 [11]. Effective and tolerable second-line treatment options following immunochemotherapy remain a significant unmet clinical need. Several studies have demonstrated that nintedanib plus docetaxel is an effective option in a second-line setting and beyond, with a manageable tolerability profile [3–6]. This study provides further evidence of the tolerability of nintedanib plus docetaxel, but with an alternative dosing schedule designed to minimize AEs.

## Supporting information

S1 Checklist TREND statement checklist. (PDF)

S1 Table AEs occurring in at least one patient overall during the on-treatment period in the treated set. (DOCX)

S1 Protocol (PDF)

## References

[1] Fukumura D, Kloepper J, Amoozgar Z, Duda DG, Jain RK. Enhancing cancer immunotherapy using antiangiogenics: opportunities and challenges. Nat Rev Clin Oncol. 2018;15(5):325–40. PubMed Central PMCID: MC29508855. doi: 10.1038/nrclinonc.2018.29 29508855 PMC5921900

[2] European Medicines Agency. Nintedanib Summary of Product Characteristics. Available at: https://www.ema.europa.eu/en/medicines/human/EPAR/vargatef. Last accessed: December 2021.

[3] Grohé C, Gleiber W, Haas S, Hammerschmidt S, Krüger S, Mueller-Huesmann H, et al. 1372P Efficacy and safety of nintedanib + docetaxel in lung adenocarcinoma patients (pts) after failure of previous immune checkpoint inhibitor therapy (ICIs): updated results from the ongoing non-interventional study (NIS) VARGADO (NCT02392455). Ann Oncol. 2020;31(Suppl4):S875–S6. 10.1016/j.annonc.2020.08.1686.

[4] Corral J, Majem M, Rodríguez-Abreu D, Carcereny E, Cortes Á A, Llorente M, et al. Efficacy of nintedanib and docetaxel in patients with advanced lung adenocarcinoma treated with first-line chemotherapy and second-line immunotherapy in the nintedanib NPU program. Clin Transl Oncol. 2019;21(9):1270–9. Epub 2019/02/17. doi: 10.1007/s12094-019-02053-7 ; PubMed Central PMCID: MC30771085. 30771085

[5] Reck M, Syrigos K, Miliauskas S, Zöchbauer-Müller S, Fischer JR, Buchner H, et al. Non-interventional LUME-BioNIS study of nintedanib plus docetaxel after chemotherapy in adenocarcinoma non-small cell lung cancer: a subgroup analysis in patients with prior immunotherapy. Lung Cancer. 2020;148:159–65. Epub 2020/09/15. doi: 10.1016/j.lungcan.2020.08.004 ; PubMed Central PMCID: MC32927350. 32927350

[6] Reck M, Kaiser R, Mellemgaard A, Douillard JY, Orlov S, Krzakowski M, et al. Docetaxel plus nintedanib versus docetaxel plus placebo in patients with previously treated non-small-cell lung cancer (LUME-Lung 1): a phase 3, double-blind, randomised controlled trial. Lancet Oncol. 2014;15(2):143–55. Epub 2014/01/15. doi: 10.1016/S1470-2045(13)70586-2 ; PubMed Central PMCID: MC24411639. 24411639

[7] Di Maio M, Perrone F, Chiodini P, Gallo C, Camps C, Schuette W, et al. Individual patient data meta-analysis of docetaxel administered once every 3 weeks compared with once every week second-line treatment of advanced non-small-cell lung cancer. J Clin Oncol. 2007;25(11):1377–82. Epub 2007/04/10. doi: 10.1200/JCO.2006.09.8251 ; PubMed Central PMCID: MC17416857. 17416857

[8] SAS Institute Inc. The data analysis for this paper was generated using SAS software. Copyright © 2020 SAS Institute Inc. SAS and all other SAS Institute Inc. product or service names are registered trademarks or trademarks of SAS Institute Inc., Cary, NC, USA. Available from: https://www.sas.com/en_us/legal/editorial-guidelines.html.

[9] Planchard D, Popat S, Kerr K, Novello S, Smit EF, Faivre-Finn C, et al. Metastatic non-small cell lung cancer: ESMO Clinical Practice Guidelines for diagnosis, treatment and follow-up. Ann Oncol. 2018;29(Suppl4):iv192–iv237. Epub 2018/10/05. doi: 10.1093/annonc/mdy275 ; PubMed Central PMCID: MC30285222. 30285222

[10] Capelletto E, Migliorino MR, Morabito A, Chiari R, Grossi F, Tiseo M, et al. Final results of the SENECA (SEcond line NintEdanib in non-small cell lung CAncer) trial. Lung Cancer. 2019;134:210–7. Epub 2019/07/20. doi: 10.1016/j.lungcan.2019.06.028 ; PubMed Central PMCID: MC31319983. 31319983

[11] Kiessling AH. Hemodynamic consequence of different pacing modes after aortic valve replacement. Heart Surg Forum. 2018;21(2):E090–e5. Epub 2018/04/17. doi: 10.1532/hsf.1785 ; PubMed Central PMCID: MC29658865. 29658865

