# Supplementary material for: Safety and tolerability of weekly docetaxel plus nintedanib: A phase I trial after first-line chemotherapy failure in NSCLC
Source: PLoS One. 2023 Oct 17;18(10):e0292307. doi: 10.1371/journal.pone.0292307 (PMC10581470; doi:10.1371/journal.pone.0292307)
Supplement: S1 Table — (DOCX) [file pone.0292307.s002.docx]

**Supporting information**

**S1 Table.** **AEs occurring in at least one patient overall during the on-treatment period.**

|  | **Nintedanib 150 mg BID + docetaxel (n = 7)** | | | **Nintedanib 200 mg BID + docetaxel (n = 7)** | | |
| --- | --- | --- | --- | --- | --- | --- |
|  | **All grades** | **Grade 1−2** | **Grade ≥3** | **All grades** | **Grade 1−2** | **Grade ≥3** |
| **Total with events** | 7 (100.0) | 2 (28.6) | 5 (71.4) | 6 (85.7) | 2 (28.6) | 4 (57.1) |
| **Diarrhea** | 5 (71.4) | 4 (57.1) | 1 (14.3) | 4 (57.1) | 4 (57.1) | 0 |
| **Cough** | 4 (57.1) | 2 (28.6) | 2 (28.6) | 0 | 0 | 0 |
| **Dyspnea** | 4 (57.1) | 2 (28.6) | 2 (28.6) | 2 (28.6) | 2 (28.6) | 0 |
| **Anemia** | 3 (42.9) | 3 (42.9) | 0 | 1 (14.3) | 1 (14.3) | 0 |
| **Epistaxis** | 3 (42.9) | 3 (42.9) | 0 | 1 (14.3) | 1 (14.3) | 0 |
| **Constipation** | 2 (28.6) | 2 (28.6) | 0 | 0 | 0 | 0 |
| **Nausea** | 2 (28.6) | 1 (14.3) | 1 (14.3) | 3 (42.9) | 3 (42.9) | 0 |
| **Asthenia** | 2 (28.6) | 2 (28.6) | 0 | 0 | 0 | 0 |
| **Fatigue** | 2 (28.6) | 1 (14.3) | 1 (14.3) | 2 (28.6) | 0 | 2 (28.6) |
| **Mucosal inflammation** | 2 (28.6) | 2 (28.6) | 0 | 0 | 0 | 0 |
| **Pain** | 2 (28.6) | 2 (28.6) | 0 | 1 (14.3) | 0 | 1 (14.3) |
| **Decreased appetite** | 2 (28.6) | 2 (28.6) | 0 | 1 (14.3) | 1 (14.3) | 0 |
| **Nail disorder** | 2 (28.6) | 2 (28.6) | 0 | 0 | 0 | 0 |
| **Neutropenia** | 1 (14.3) | 0 | 1 (14.3) | 1 (14.3) | 1 (14.3) | 0 |
| **Stomatitis** | 1 (14.3) | 1 (14.3) | 0 | 1 (14.3) | 1 (14.3) | 0 |
| **Pyrexia** | 1 (14.3) | 1 (14.3) | 0 | 1 (14.3) | 1 (14.3) | 0 |
| **AST increased** | 1 (14.3) | 1 (14.3) | 0 | 1 (14.3) | 1 (14.3) | 0 |
| **GGT increased** | 1 (14.3) | 1 (14.3) | 0 | 1 (14.3) | 0 | 1 (14.3) |
| **Malignant neoplasm progression** | 1 (14.3) | 0 | 1 (14.3) | 1 (14.3) | 0 | 1 (14.3) |
| **Headache** | 1 (14.3) | 1 (14.3) | 0 | 1 (14.3) | 0 | 1 (14.3) |
| **Onychalgia** | 1 (14.3) | 1 (14.3) | 0 | 1 (14.3) | 1 (14.3) | 0 |
| **Hypertension** | 1 (14.3) | 0 | 1 (14.3) | 1 (14.3) | 0 | 1 (14.3) |
| **Vomiting** | 0 | 0 | 0 | 2 (28.6) | 2 (28.6) | 0 |
| **Dizziness** | 0 | 0 | 0 | 2 (28.6) | 1 (14.3) | 1 (14.3) |
| **Alopecia** | 0 | 0 | 0 | 2 (28.6) | 2 (28.6) | 0 |
