# Supplementary material for: Safety and tolerability of weekly docetaxel plus nintedanib: A phase I trial after first-line chemotherapy failure in NSCLC
Source: PLoS One. 2023 Oct 17;18(10):e0292307. doi: 10.1371/journal.pone.0292307 (PMC10581470; doi:10.1371/journal.pone.0292307)
Supplement: S1 Protocol — (PDF) [file pone.0292307.s003.pdf]

Clinical Trial Protocol

**Document Number:**		**c03239885-01**
**EudraCT No.:**	2015-000317-52	
**BI Trial No.:**	1199.224	
**BI Investigational Product(s):**	nintedanib, BIBF 1120	
**Title:**	An open label Phase I of oral nintedanib plus weekly docetaxel therapy in patients with locally advanced or metastatic lung adenocarcinoma after failure of platinum-based first line chemotherapy	
**Brief Title:**	Phase I nintedanib and weekly docetaxel in lung adenocarcinoma.	
**Clinical Phase:**	Phase I	
**Trial Clinical Monitor:**	Dr Hélène de Mont-Serrat<br>Boehringer Ingelheim France<br>12, rue André Huet<br>51721 Reims Cedex<br>France<br>Phone : + 33 (0)3 26 50 46 97<br>Mobile : + 33 (0)6 68 45 57 67	
**Coordinating Investigator:**	Professor Virginie Westeel<br>CHU Besançon<br>Hôpital Jean Minjoz<br>1 boulevard Fleming<br>25030 Besançon Cedex<br>Tel: + 33 (0)3 81 66 82 04	
**Status:**	Final Protocol	
**Version and Date:**	**Version:**	**Date:**
	Version 1.0	04 Nov 2015

CLINICAL TRIAL PROTOCOL SYNOPSIS

**Name of company:**		**Boehringer Ingelheim**	
**Name of finished product:**		**Vargatef**	
**Name of active ingredient:**		**Nintedanib, BIBF1120**	
**Protocol date:**<br>**04 Nov 2015**	**Trial number:**<br>**1199.224**		**Revision date:**

**Title of trial:**	An open label Phase I of oral nintedanib plus weekly docetaxel therapy in patients with locally advanced or metastatic lung adenocarcinoma after failure of platinum-based first line chemotherapy
**Coordinating Investigator:**	Professor Virginie Westeel<br>CHU Besançon<br>Hôpital Jean Minjoz<br>1 boulevard Fleming<br>25030 Besançon Cedex<br>Tel : + 33 (0)3 81 66 82 04
**Trial site(s):**	Multi-centre trial conducted in 2 countries (2 sites in France and 2 in Germany)
**Clinical phase:**	I
**Objective(s):**	To determine the MTD of nintedanib twice daily (b.i.d.) plus weekly docetaxel 35 mg/m2 (day1, day8, day15 following a 28 days cycle) in patients with locally advanced or metastatic lung adenocarcinoma after failure of platinum-based first line chemotherapy
**Methodology:**	Open - label
**No. of patients:**	35
**total entered:**	Up to 30 patients (24 evaluable patients)
**each treatment:**	NA
**Diagnosis :**	Patients with locally advanced or metastatic lung adenocarcinoma after failure of platinum-based first line chemotherapy
**Main criteria for inclusion:**	1. Patient aged 18 or older at the date of informed consent<br>2. Patients with histologically/cytologically confirmed locally advanced or metastatic lung adenocarcinoma after failure of first line platinum-based chemotherapy (patients with non-target lesions only are eligible) First line chemotherapy may include continuation or switch maintenance therapy. One prior adjuvant and/or neoadjuvant chemotherapy is accepted. Prior immunotherapy is allowed.<br>3. Patients who have a life expectancy of at least 3 months<br>4. Patients who are Eastern Cooperative Oncology Group (ECOG) performance status (PS) of 0 or 1 at screening<br>5. Patient must have given written informed consent in accordance with ICH-GCP
**Test product(s):**	Nintedanib with weekly docetaxel in second line chemotherapy.

**Name of company:**		**Boehringer Ingelheim**	
**Name of finished product:**		**Vargatef**	
**Name of active ingredient:**		**Nintedanib, BIBF1120**	
**Protocol date:**<br>**04 Nov 2015**	**Trial number:**<br>**1199.224**		**Revision date:**

**dose:**	100 mg b.i.d (dose level – 1, de-escalation dose), 150 mg b.i.d (dose level 1, starting dose), 200 mg b.i.d (dose level 2, escalation dose) continuously except on the days of docetaxel infusion.<br>Continuous administration of nintedanib 200 mg b.i.d without interruption on docetaxel infusion days (dose level 3)<br>If the MTD reaches the nintedanib dose of 200 mg b.i.d (with or without interruption on the docetaxel infusion days), 6 additional patients will be included in the expansion cohort at the MTD level.
**mode of administration:**	Oral
**Backbone chemotherapy**	Docetaxel
**dose:**	35 mg/m² at Day 1, at Day 8, at Day 15 every 28 days cycle.
**mode of administration:**	i.v.
**Duration of treatment:**	Twenty-eight days per treatment cycle (docetaxel administration on day 1, day 8 and day 15 and nintedanib every day except the days of docetaxel infusion and continuously even during docetaxel infusion in the dose-level 3 cohort). Treatment cycles may be repeated until disease progression, development of intolerable toxicity, withdrawal of consent or until other discontinuation criteria are met.<br>In case patients have to discontinue docetaxel or nintedanib for reasons other than progression, patients can continue therapy with remaining drug as monotherapy.
**Endpoints**	Primary endpoints :<br>1. MTD of nintedanib in combination with weekly docetaxel.<br>2. Number of patients with Dose Limiting Toxicity (DLT) for nintedanib in combination with docetaxel during Cycle 1<br>Secondary endpoints :<br>There are no formal secondary safety endpoints.<br>There is no primary or secondary endpoint for efficacy.
**Safety criteria:**	The safety evaluation is based on the occurrence of DLTs during cycle 1 to determine the MTD, frequency and severity of adverse events according to CTCAE version 4.0, on safety laboratory parameters, ECOG status and electrocardiogram (ECG)

**Name of company:**		**Boehringer Ingelheim**	
**Name of finished product:**		**Vargatef**	
**Name of active ingredient:**		**Nintedanib, BIBF1120**	
**Protocol date:**<br>**04 Nov 2015**	**Trial number:**<br>**1199.224**		**Revision date:**
**Statistical methods:**	Descriptive statistics.		

FLOW CHART

Combination therapy with nintedanib plus docetaxel. Also to be used for docetaxel monotherapy in case nintedanib is discontinued

**Trial period**	**Screening**	**Treatment period (28 days per cycle)**										
		**Cycle 1**			**Cycle 2**			**Cycle n onwards**				
Visit		C1D1[3]	C1D8[3]	C1D15[3]	C2D1[3]	C2D8[3]	C2D15[3]	CnD1[3]	CnD8[3]	CnD15[3]	**EOT[1] ± 7**	**EOR[2]+ 7**
Day	-14 ~ -1	1	8±2	15±2	1±2	8±2	15±2	1± 2	8 ± 2	15 ± 2		
Informed consent:	X											
Demographics	X											
Medical history	X											
Inclusion/exclusion criteria	X											
Patient registration [4]	X											
Eligibility for further courses			X	X	X	X	X	X	X	X		
Physical examination	X	X			X			X			X	
ECOG PS	X	X			X			X			X	X
Vital signs[5] (blood pressure, heart rate, body temperature)	X	X[5]	X[5]	X[5]	X[5]	X[5]	X[5]	X[5]	X[5]	X[5]	X	
Height	X											
Weight	X	X	X	X	X	X	X	X	X	X	X	
ECG (resting)[6]	X[7]	X[6]						X[6]			X[6]	
Safety laboratory test : Haematology and biochemistry[7-8]	X[7]	X[8]	X[8]	X[8]	X[8]	X[8]	X[8]	X[8]	X[8]	X[8]	X[8]	
Coagulation parameters[7-9]	X[7]	X[9]			X[9]			X[9]			X[9]	
Urine examination[7-9]	X[7]	X[9]			X[9]			X[9]			X[9]	
Pregnancy test[10]	X[10]	X[10]						X[10]			X[10]	
Premedication for docetaxel treatment[11]		X[11]	X[11]	X[11]	X[11]	X[11]	X[11]	X[11]	X[11]	X[11]		
Docetaxel treatment		X	X	X	X	X	X	X	X	X		
Dispensing of nintedanib[12]		X			X[12]			X[12]				
Nintedanib treatment[13]		X[13]	X[13-14]	X[13-14]	X[13-14]	X[13-14]	X[13-14]	X[13]	X[13]	X[13]		
Compliance check[15]					X[15]			X[15]			X	
Concomitant therapy	X	X	X	X	X	X	X	X	X	X	X	X
Tumour assessment (CT/MRI)[16-17-18]	X[16]							X[17]			X[18]	
Blood sampling for PK analysis[19]			X[19]	X[19]	X[19]	X[19]	X[19]					
AEs/SAEs	X	X	X	X	X	X	X	X	X	X	X	X
Other subsequent anti-cancer therapy												X

1 End of treatment (EOT) visit is to be performed within 7 days after permanent discontinuation of last trial medication. If the patient permanently discontinues the trial medication during treatment interruption, see Section 6.2.3.1.
2 EOR: End of residual effect period (REP) visit is to be performed at 28 days or later after permanent discontinuation of last trial medication.
3 Day 1, Day 8 and Day 15 of each cycle may be postponed for up to 2 days.
4 Registration will be performed following RDC. The IRT system will assign nintedanib and docetaxel kit numbers.
5 Vital signs at Day 1, Day 8 and Day 15 of each cycle at pre and post-dose of docetaxel infusion.
6 Resting electrocardiogram (ECG) to be performed on day 1 of every odd number of treatment cycles (can be performed within 2 days prior to docetaxel administration) and at EOT.
7 If ECG and safety laboratory data (Heamatology, biochemistry, coagulation parameters and urine examination) were obtained within 7 days prior to registration including before obtaining informed consent, that data may be used for registration at screening.
8 Safety laboratory test (haematology and biochemistry) to be performed within 2 days prior to docetaxel administration (CnDay 1, CnDay 8 and Cn Day 15) and at EOT.
9 Coagulation parameters and urinalysis (see Section 5.3.3) to be performed on day 1 of each cycle (can be performed within 2 days prior to docetaxel infusion) and at EOT
10 In women of childbearing potential (i.e. women who have not had a hysterectomy, premenopausal, or have been amenorrhoeic for less than 12 months), a pregnancy test will be performed by urinalysis or serum β-human chorionic gonadotropin (β-hCG) at screening, pre-dose on Day 1 of Cycle 1 (if the test at screening has been performed beyond 72 hours on Day 1 of Cycle 1, it may be skipped), every odd number of treatment cycle (3, 5, and so on) within 2 days before administration of docetaxel, and at the EOT.
11 All patients should be pre-medicated prior to docetaxel treatment (see Section 4.1.4).
12 If Day 1 of the next treatment cycle is planned later than 28 days from Day 1 of current cycle, one more medication kit of nintedanib can be dispensed.
13 Nintedanib will be administered from Day 2 to Day 7, from Day 9 to Day 14 and from Day 16 to Day 27 on each treatment cycle but not on the days of docetaxel infusion. For the cohort of patients, nintedanib 200 mg b.i.d (dose level 3) will be administered continuously from Day 1 to Day 28 without interruption during docetaxel infusion.
14 The patients of the cohort dose level 3 (nintedanib 200 mg b.i.d) will take the morning dose of nintedanib at the hospital at days of PK blood sample.
15 Check nintedanib actual drug intake and collect unused nintedanib capsules at the next dispense of nintedanib.
16 Tumour assessment (CT / MRI) taken within 28 days prior to start of Cycle 1 may be used as the baseline scan.
17 Tumour assessments according to Response Evaluation Criteria in Solid Tumours (RECIST) version 1.1 have to be performed on Day 1 of every odd number of treatment cycles from Cycle 3 (within 7 days prior to planned date) until tumour progression. Imaging can be performed outside of scheduled visit if progression is suspected.
18 If a patient discontinues from the study without progression, tumour assessment should be performed if not done in the past 21 days.
19 Blood samples for PK analysis (nintedanib) will be drawn in patients treated with nintedanib at 200 mg b.i.d continuously (dose level 3). Just before nintedanib and docetaxel administration, PK samples will be withdrawn at C1D8, C1D15, at C2D1, C2D8 and C2D15. Detailed schedule of PK blood sampling is provided in Appendix 10.2.

Monotherapy with nintedanib (after permanent discontinuation of docetaxel treatment)

Trial period	Treatment period (28 days per cycle)	EOT[1]	EOR[2]
	Cycle n		
Visit	Cn Day1		
Day	1 [3]		
Eligibility for the next cycle	X		
Physical examination	X	X	
ECOG PS	X	X	X
Vital sign (blood pressure, heart rate, body temperature)	X	X	
Weight	X[4]	X	
ECG (resting)	X[4]	X	
Safety laboratory test (Haematology and biochemistry) and coagulation parameters.[4]	X[4]	X	
Urine examination [4]	X[4]	X	
Pregnancy test[5]	(X)[5]	X	
Dispensing of nintedanib[6]	X[6]	X	
Nintedanib treatment[7]	X	X	
Compliance check[8]	X	X	
Concomitant therapy	X	X	X
Tumour assessment (CT/MRI)	(X)[9]	X[10]	
AEs/SAEs	X	X	X
Other subsequent anti-cancer therapy			X

1. EOT visit is to be performed within 7 days after permanent discontinuation of trial medication. If the patient permanently discontinues the trial medication during treatment interruption, see Section 6.2.3.
2. EOR visit is to be performed at 28 days or later after permanent discontinuation of trial medication.
3. Day 1 of each cycle may be postponed for administrative reasons for up to 7 days.
4. Examinations such as weight, ECG, safety laboratory test, and urinalysis (see Sections 5.3.3 and 5.3.4) can be performed within 2 days before the scheduled visit.
5. In women of childbearing potential (i.e. women who have not had a hysterectomy, premenopausal, or have been amenorrhoeic for less than 12 months), a pregnancy test will be performed by urinalysis or serum β-hCG every odd number of treatment cycle (3, 5, and so on) within 2 days before treatment and at the EOT.
6. If Day 1 of the next treatment cycle is planned later than 28 days from Day 1 of current cycle, one more medication kit of nintedanib can be dispensed.
7. Nintedanib will be administered from Day 1 to Day 28 on each treatment cycle.
8. Check nintedanib actual drug intake and collect unused nintedanib capsules at the next dispense of nintedanib.
9. Tumor assessments according to RECIST version 1.1 have to be performed on Day 1 of every odd number of treatment cycles from Cycle 3 (within 14 days prior to planned date) until tumour progression. Imaging can be performed outside of scheduled visit if progression is suspected.
10. If a patient discontinues from the study without progression, tumour assessment should be performed if not done in the past 21 days

TABLE OF CONTENTS

TITLE PAGE 1
CLINICAL TRIAL PROTOCOL SYNOPSIS 2
FLOW CHART 5
TABLE OF CONTENTS 8
ABBREVIATIONS 12
1. INTRODUCTION 16
1.1 MEDICAL BACKGROUND 16
1.2 DRUG PROFILE 18
1.2.1 Nintedanib 18
1.2.2 Docetaxel 20
1.2.3 Combination of nintedanib and docetaxel 21
2. RATIONALE, OBJECTIVES, AND BENEFIT - RISK ASSESSMENT 22
2.1 RATIONALE FOR PERFORMING THE TRIAL 22
2.2 TRIAL OBJECTIVES 22
2.3 BENEFIT - RISK ASSESSMENT 22
3. DESCRIPTION OF DESIGN AND TRIAL POPULATION 24
3.1 OVERALL TRIAL DESIGN AND PLAN 24
3.1.1 Administrative structure of the trial 25
3.2 DISCUSSION OF TRIAL DESIGN, INCLUDING THE CHOICE OF CONTROL GROUP(S) 26
3.3 SELECTION OF TRIAL POPULATION 26
3.3.1 Main diagnosis for study entry 27
3.3.2 Inclusion criteria 27
3.3.3 Exclusion criteria 27
3.3.4 Removal of patients from therapy or assessments 30
3.3.4.1 Removal of individual patients: 30
3.3.4.2 Discontinuation of the trial by the sponsor 30
3.3.4.3 Patient replacement 31
4. TREATMENTS 32
4.1 TREATMENTS TO BE ADMINISTERED 32
4.1.1 Identity of BI investigational product(s) and combination product(s) 32
4.1.1.1 Nintedanib (investigational medical product) 32
4.1.2 Method of assigning patients to treatment groups 33
4.1.3 Selection of doses in the trial 35
4.1.3.1 General recommendations for dose selection in the present trial 35
4.1.3.1.1 Nintedanib 35
4.1.3.1.2 Docetaxel 35
4.1.4 Drug assignment and administration of doses for each patient 36

4.1.4.1 Treatment cycle intervals 36
4.1.4.1.1 Treatment criteria for docetaxel therapy 37
4.1.4.1.2 Stopping criteria for docetaxel therapy 37
4.1.4.1.3 Temporary treatment interruption of nintedanib treatment 37
4.1.4.1.4 Retreatment criteria for nintedanib 38
4.1.4.2 Dose reduction scheme of nintedanib (given in combination or as monotherapy) 38
4.1.5 Blinding and procedures for unblinding 40
4.1.6 Packaging, labelling, and re-supply 41
4.1.7 Storage conditions 41
4.1.8 Drug accountability 42
4.2 CONCOMITANT THERAPY, RESTRICTIONS, AND RESCUE TREATMENT 43
4.2.1 Rescue medication, emergency procedures, and additional treatment(s) 43
4.2.2 Restrictions 43
4.2.2.1 Restrictions regarding concomitant treatment 43
4.2.2.2 Restrictions on diet and life style 44
4.3 TREATMENT COMPLIANCE 44
5. VARIABLES AND THEIR ASSESSMENT 46
5.1 TRIAL ENDPOINTS 46
5.1.1 Primary Endpoint(s) 46
5.1.2 Secondary Endpoint(s) 47
5.1.3 Further Endpoint(s) 47
5.1.3.1 Pharmacokinetics 47
5.1.3.2 Endpoint(s) of efficacy 47
5.2 ASSESSMENT OF EFFICACY 48
5.2.1 Assessment of efficacy following RECIST criteria (version 1.1) (Appendix 10.3). 48
5.2.2 Progression of disease 48
5.3 ASSESSMENT OF SAFETY 49
5.3.1 Physical examination 49
5.3.2 Vital Signs 49
5.3.3 Safety laboratory parameters 49
5.3.4 Electrocardiogram 50
5.3.5 Other safety parameters: 50
5.3.6 Assessment of adverse events 50
5.3.7 Adverse event collection and reporting 54
5.4 DRUG CONCENTRATION MEASUREMENTS AND PHARMACOKINETICS 56
5.4.1 Assessment of Pharmacokinetics 56
5.4.2 Methods of sample collection 56
5.4.3 Analytical determinations 56
5.4.4 Pharmacokinetic – Pharmacodynamic Relationship: 56
5.5 ASSESSMENT OF EXPLORATORY BIOMARKERS 57
5.5.1 Biobanking 57

5.6 OTHER ASSESSMENTS 57
5.7 APPROPRIATENESS OF MEASUREMENTS 57
6. INVESTIGATIONAL PLAN 58
6.1 VISIT SCHEDULE 58
6.2 DETAILS OF TRIAL PROCEDURES AT SELECTED VISITS 58
6.2.1 Screening and treatment period(s) 58
6.2.2 Treatment cycles 59
6.2.2.1 Cycle 1 Day 1 (C1D1) 59
6.2.2.2 Cycle 1 Day 8 (C1D8) and Cycle 1 Day 15 (C1D15) 59
6.2.2.3 Cn Day 1 ± 2 days of every treatment onwards 60
6.2.2.4 Cn Day 8 and Cn Day 15 of treatment cycle 2 onwards : 61
6.2.2.5 Cn (Day 1) of a treatment cycle with monotherapy nintedanib 62
6.2.2.6 Optional visits between scheduled visits 62
6.2.3 End of treatment (EOT) and EOR (End of Residual Effect Period (REP) 63
6.2.3.1 End of treatment 63
6.2.3.2 EOR (End of Residual Effect Period (REP) 63
6.2.3.3 Trial completion for an individual patient 64
7. STATISTICAL METHODS AND DETERMINATION OF SAMPLE SIZE 65
7.1 STATISTICAL DESIGN - MODEL 65
7.2 NULL AND ALTERNATIVE HYPOTHESES 65
7.3 PLANNED ANALYSES 65
7.3.1 Primary endpoint analyses 65
7.3.2 Secondary endpoint analyses 65
7.3.3 Further endpoint analyses 65
7.3.4 Safety analyses 66
7.3.5 Pharmacokinetic analyses 66
7.4 INTERIM ANALYSES 67
7.5 HANDLING OF MISSING DATA 67
7.5.1 Plasma drug concentration - time profiles 67
7.6 RANDOMISATION 67
7.7 DETERMINATION OF SAMPLE SIZE 67
8. INFORMED CONSENT, DATA PROTECTION, TRIAL RECORDS 68
8.1 TRIAL APPROVAL, PATIENT INFORMATION, AND INFORMED CONSENT 68
8.2 DATA QUALITY ASSURANCE 69
8.3 RECORDS 69
8.3.1 Source documents 69
8.3.2 Direct access to source data and documents 70
8.3.3 Storage period of records 70
8.4 LISTEDNESS AND EXPEDITED REPORTING OF ADVERSE EVENTS 70
8.4.1 Listedness 70
8.4.2 Expedited reporting to health authorities and IEC / IRB 71

8.6 END OF TRIAL....................71
8.7 PROTOCOL VIOLATIONS....................71
8.8 COMPENSATION AVAILABLE TO THE PATIENT IN THE EVENT OF TRIAL RELATED INJURY....................71

9. REFERENCES....................72

9.1 PUBLISHED REFERENCES....................72
9.2 UNPUBLISHED REFERENCES....................75

10. APPENDICES....................77

10.1 CLINICAL EVALUATION OF LIVER INJURY....................77
10.1.1 Introduction....................77
10.1.2 Procedures....................77
10.2 PHARMACOKINETIC ANALYSES....................79
10.2.1 Handling procedure of blood samples for plasma concentration-time measurements....................79
10.2.2 Time schedule for Pharmacokinetic (PK) blood sampling....................79
10.3 RECIST CRITERIA (VERSION 1.1)....................80
10.4 STATISTICAL APPENDIX....................85

11. DESCRIPTION OF GLOBAL AMENDMENT(S)....................87

ABBREVIATIONS

AE	adverse event
AESI	adverse event of special interest
ALK	anaplastic lymphoma kinase
ALP	alkaline phosphatase
ALT	alanine amino transferase
ANC	absolute neutrophil count
aPTT	activated partial thromboplastin time
AST	aspartate aminotransferase
ATP	adenosine triphosphate
AUC	area under the curve
BI	Boehringer Ingelheim
b.i.d.	twice daily
β-hCG	β-human chorionic gonadotropin
BLQ	below the lower limit of quantification
BSA	body surface area
CA	competent authority
CK	creatine kinase
CI	confidence interval
CML	Local Clinical Monitor
C_{max}	maximum measured concentration of the analyte in plasma
$C_{pre(,ss)}$	Pre-dose concentration of the analyte in plasma (at steady-state)
CR	complete response
CRA	clinical research associate
CRO	contract research organization
CT	computerized tomography
CTCAE	Common Terminology Criteria for Adverse Events – Version 4.
CTP	clinical trial protocol
CTR	clinical trial report
CYP	cytochrome p
D35	docetaxel 35 mg/m^2
D75	docetaxel 75 mg/m^2
DCR	disease control rate
DEDP	drug exposure during pregnancy
DILI	drug induced liver injury
DLT	dose limiting toxicity
DMC	Data Monitoring Committee
ECG	electrocardiogram
eCRF	electronic case report form
ECOG PS	Eastern Cooperative Oncology Group performance status
EDTA	ethylenediaminetetraacetic acid
EGFR	epidermal growth factor receptor
EMA	European Medicines Agency
EML4-	echinoderm microtubule-associated protein-like 4 anaplastic lymphoma

ALK	kinase
EOT	end of treatment
EOR	end of residual effect period
EU	European Union
FDA	Food and Drug Administration
FGF	fibroblast growth factor
FGFR	fibroblast growth factor receptor
mFOLFOX	modified FOLFOX (Folinic acid, leucoverin, 5 FU and oxaliplatin)
G-CSF	granulocyte colony stimulating factor
GCP	Good Clinical Practice
γ-GT	gamma glutamyl transferase
GI	gastrointestinal
h	hour
HBV	hepatitis B virus
HCV	hepatitis C virus
HDV	hepatitis D virus
HIV	human immunodeficiency virus
HR	hazard ratio
IC_{50}	half maximal (50%) inhibitory concentration
ICH	International Conference on Harmonisation
IEC	Independent Ethics Committee
IMP	Investigational product
INN	international approved names for pharmacopoeial substances
INR	international normalised ratio
IPF	idiopathic pulmonary fibrosis
IRB	institutional review board
IRT	interactive response technology
ISF	Investigator Site File
IWRS	interactive web-based response system
LC-	liquid chromatography-tandem mass spectrometry
L	litre
LD	longest diameter
LDH	lactate dehydrogenase
m^2	squaral meter
MAPK	mitogen-activated protein kinase
MedDRA	Medical Dictionary for Drug Regulatory Activities
mg	Milligram
ml	millilitre
MRI	magnetic resonance imaging
MRT	mean residence time
MTD	maximum tolerated dose
N100	nintedanib 100 mg b.i.d.
N150	nintedanib 150 mg b.i.d.
N200	nintedanib 200 mg b.i.d.
NC	not calculated

NE	not evaluable
NIMP	non investigational product
nM	nano molar
NOA	not analysed
NOR	no valid result
NOS	no sample available
NSCLC	non-small cell lung cancer
NYHA	New York Heart Association
OC	ovarian cancer
OPU	operative unit
OR	objective response
ORR	objective response rate
OS	overall survival
PD	progressive disease
PD-1	programmed death 1 receptor
PK/PD	Pharmacokinetics/Pharmacodynamics,
PD-L ½	Ligand of programmed death 1 receptor
PDGF	platelet derived growth factor
PDGFR	platelet derived growth factor receptor
PFS	progression free survival
PGx	pharmacogenomics
P-gp	P-glycoprotein
PK	pharmacokinetics
PMDA	Pharmaceuticals and Medical Devices Agency
PR	partial response
PS	performance status
PT	prothrombin time
RDC	remote data capture
RECIST	Response Evaluation Criteria in Solid Tumours
REP	residual effect period
SAE	serious adverse event
SD	stable disease
SOP	standard operating procedures
SPC	summary of product characteristics
SRC	safety review committee
SUSAR	suspected unexpected serious adverse reactions
TCM	Trial Clinical Monitor
TCPK	Trial Clinical Pharmacokineticist
TDM	Trial Data Manager
TDMAP	trial data management and analysis plan
TKI	tyrosine kinase inhibitor
TSH	thyroid-stimulating hormone
TSTAT	trial statistician
TMF	Trial Master File
TTP	Time to tumour progression
ULN	upper limit of normal

US	United States
VEGF	vascular endothelial growth factor
VEGFR	vascular endothelial growth factor receptor
WBC	white blood count
WHO-DD	World Health Organisation Drug dictionary

1. INTRODUCTION

1.1 MEDICAL BACKGROUND

Lung cancer is one of the leading causes of cancer death. In 2012, 1.8 million people were newly diagnosed with lung cancer worldwide, and 1.6 million deaths were caused by lung cancer i.e. 19.4% of the overall cancer mortality [R14-2236]. Non-small cell lung cancer (NSCLC) accounts for approximately 85% of lung cancer cases [R08-2617]. Histological subtypes of adenocarcinoma and squamous cell carcinoma each account for about 30 to 50% of NSCLC cases [P11-08063]. The majority of patients with lung cancer are diagnosed after the disease has progressed to a more advanced stage. The prognosis for the majority of patients with advanced stage disease has not changed significantly in the past decade. With an overall 5-year survival rate of 9 to 13%, the treatment of NSCLC remains a major clinical challenge [R11-0052].

Several treatment options are available for NSCLC patients in the first-line setting. These include platinum-combination regimens and, as a more recent addition to the treatment option, targeted therapies such as epidermal growth factor receptor tyrosine kinase inhibitors (EGFR TKIs) or echinoderm microtubule-associated protein-like 4 anaplastic lymphoma kinase (ALK) inhibitors in patients carrying EGFR mutation and ALK translocation positive, respectively. In the second line setting, the development of treatment options has significantly lagged behind developments in the first-line setting, and there is a high unmet medical need to improve the treatment options for patients with advanced NSCLC in the second line setting.

The understanding of NSCLC has evolved tremendously over recent years. Today, NSCLC is viewed as a cluster of different disease variants that can be identified by histological subtyping or genetic characterisation of tumours harboring specific mutations. These different variants and subtypes have been shown to respond differently to different therapies. Examples include pemetrexed that shows benefit in one histological subtype and not the other; differentiation by genetic alterations such as EGFR mutations where agents including afatinib, erlotinib and gefitinib show the greatest improvement in outcomes; and differentiation by molecular rearrangements such as EML4-ALK where crizotinib has recently demonstrated dramatic prolongation in progression free survival (PFS) [R04-4507, R07-1199, R13-0232]. There is growing evidence that differences in tumour biology make it necessary to develop specific treatment approaches for patients with different tumour histologies, and that the further clinical development in NSCLC needs to focus on specifically defined patient populations and histological subtypes [R13-3173].

Angiogenesis is involved in tumour growth and development of metastases. Vascular endothelial growth factor (VEGF) and its high affinity receptor VEGFR-2 are crucial for the formation of new tumour vessels. In addition, there is preclinical evidence that fibroblast growth factor (FGF) and platelet-derived growth factor (PDGF) and their associated receptor tyrosine kinases substantially contribute to tumour angiogenesis. The VEGF–VEGFR-2 axis, besides promoting angiogenesis, may also be involved in stimulating growth of tumour cells

themselves via autocrine or paracrine interactions [R13-4194, R13-4197]. Increased expression of VEGF, found in most solid tumours including NSCLC, has been associated with an increased risk of recurrence, metastases and death [R13-4198]. In addition to their roles in angiogenesis, these pathways are also expressed in tumour cells and can play a role in tumour growth, metastatic spread, and therapeutic resistance. For example, copy number gains in the VEGFR-2 gene, have been observed in 20-30% of NSCLC tumours and is associated with recurrence after adjuvant therapy and chemo-resistance [R13-4198]. Likewise, the FGFR1 gene is also amplified in NSCLC and has been identified as a potential therapeutic target [R11-1327].

First line use of bevacizumab, an approved anti-VEGF monoclonal antibody in combination with platinum based chemotherapy, has been shown to improve overall survival (OS) beyond 1 year for patients with advanced non-squamous pathology of NSCLC compared with chemotherapy alone [R07-1161].

Despite the efficacy of a number of first-line treatments, including targeted therapies, the vast majority of patients with advanced-stage NSCLC will inevitably experience disease progression. Since a decade only few advances were made in the management after first-line progression for patients with NSCLC who are not candidates for specific targeted therapies. Recently, two anti-angiogenic agents have been approved in patients with advanced NSCLC after first-line chemotherapy in combination with docetaxel. The results of the LUME-Lung 1 trial (NCT00805194; study 1199.13) led to the EU approval of nintedanib, in combination with docetaxel, for the treatment of patients with locally advanced, metastatic or locally recurrent NSCLC of adenocarcinoma tumour histology after first-line chemotherapy (current SmPC). Subsequently, results of the REVEL trial (NCT01168973; study 13852) led to the US approval of ramucirumab in combination with docetaxel for patients with metastatic NSCLC with disease progression on or after platinum-based chemotherapy (current SmPC).

Immunotherapy, a novel therapeutic approach to treating NSCLC, was evaluated. Circulating immune cells may have the ability to recognize infiltrate and eliminate some incipient cancer cells, but some evade immune surveillance and immune system-mediated cell death [R12-2552]. Recent studies have improved the understanding of the molecular basis for this phenomenon, and aided in the identification of anticancer approaches that act by modulating the immune system directly.

Immune checkpoint inhibition, involving the programmed death 1(PD-1) receptor and its ligands (PD-L1/2) has clarified the role of these approaches in tumor-induced immune suppression and has been a critical advancement in immunotherapeutic drug development. This new treatment approach is undergoing extensive investigation in NSCLC and other malignancies. They indicate important response rates and high quality responses or prolonged duration. Unlike standard chemotherapy or targeted agents, which act directly on the tumor cells, immune checkpoint inhibitors work by restoring the immune system's capacity to eradicate tumors.

Nivolumab, is a fully human, high affinity, immunoglobulin (Ig) G4 monoclonal antibody to

PD-L1. Phase III trials for nivolumab versus docetaxel in patients with squamous (CheckMate-017; NCT01642004) and non-squamous (CheckMate-057; NCT01673867) histology after failure of platinum-based doublet chemotherapy have both been stopped early following planned interim analyses and a DMC assessment, which concluded that both studies met their primary endpoint, demonstrating superior OS in patients receiving nivolumab, when compared to the control arm [R15-3058 and R15-3057] .
With the advent of these new drugs, more therapeutic options are needed since patients eventually relapse. New treatments or treatment combinations are needed to improve outcomes for these patients.

1.2 DRUG PROFILE

1.2.1 Nintedanib

Nintedanib is approved in the EU in 2014 for use in combination with docetaxel to treat locally advanced, metastatic or locally recurrent NSCLC of adenocarcinoma histology, in patients who have progressed on first-line chemotherapy. It is an oral triple angiokinase inhibitor. It is an indolinone derivative that blocks the kinase activity of VEGFR 1-3, PDGFR α and β, and FGFR 1-3 by occupying the adenosine triphosphate (ATP) binding pocket [U02-1482; U11-1947-01]. VEGFR-2 is considered to be the crucial receptor involved in the formation as well as the maintenance of tumour vasculature. Nintedanib inhibits VEGFR 1-3 kinases with IC50 values of 34, 21, and 13 nM, respectively; PDGFR α and β with IC50 values of 59 nM and 65 nM; and FGFR 1 to 4 with IC50 values of 69, 37, 137, and 604 nM, respectively [P08-08684]. Additional kinases (Flt3, Lck, Src kinases) are inhibited by nintedanib with similar potency [P08-08684, R13-3128].

In vitro within cells, nintedanib has a sustained duration of action. Pulse-chase experiments with VEGFR 2-transfected NIH3T3 fibroblasts demonstrated that after 1-h exposure to 50 nM nintedanib, the auto-phosphorylation of VEGFR-2 was blocked for at least 32 h [U02-1109]. This sustained receptor blockade indicates the potential for a long-lasting anti-angiogenic effect. Nintedanib was shown to interfere with the mitogen-activated protein kinase (MAPK) and Akt signalling pathways downstream of the receptor tyrosine kinases in endothelial cells, pericytes, and smooth muscle cells, resulting in down-regulation of mitogenic signals and induction of apoptosis [U08-1946].

The clinical development programme for nintedanib includes several cancer indications: NSCLC, ovarian cancer (OC), colorectal cancer, renal cell carcinoma, and hepatocellular carcinoma. Nintedanib has been studied as well in non-cancer indication idiopathic pulmonary fibrosis (IPF) and has been approved as monotherapy by both US-FDA and EMA in 2014.

A wide range of nintedanib monotherapy doses was investigated in Phase I and II trials, by using the doses from 50 to 450 mg once daily and from 150 to 300 mg twice daily (b.i.d). In Phase I dose escalation trials the predominant dose limiting toxicities (DLTs) were

gastrointestinal adverse events (AEs) and fully reversible increases of liver enzymes (ALT, AST, gamma glutamyl transferase [γ-GT]) not accompanied by relevant bilirubin increases, and the maximum tolerated dose (MTD) was determined as 250 mg b.i.d. in Caucasian patients [U10-1846-01].

In Phase I trials combining nintedanib with docetaxel, pemetrexed, paclitaxel/carboplatin or mFOLFOX6, the recommended dose of nintedanib was established to be 200 mg b.i.d. in Caucasians [U02-1482]. The pattern of AEs was comparable to the AE profile observed in Phase I monotherapy trials except for the chemotherapy-related AEs. Based on the overall safety profile from all Phase I and Phase II trials, a nintedanib dose of 200 mg b.i.d. was selected for further Phase III trials.

Of over 3300 cancer patients included in 3 Phase III trials in NSCLC or OC, approximately 1900 patients have received nintedanib in combination with the cytotoxic chemotherapy that is standard for the respective indication, namely, docetaxel, pemetrexed or paclitaxel/carboplatin.

After administration as a soft gelatin capsule, nintedanib is absorbed quickly; maximum plasma concentrations are reached within 2 to 4 h. Nintedanib follows at least bi-phasic disposition kinetics. After intravenous administration, it showed a volume of distribution of 1050 L. Non-clinical data suggest a homogenous distribution of the drug, with the exception of the central nervous system, and no marked affinity or retention in any tissue. Nintedanib undergoes a high first-pass metabolism and is primarily metabolised by hydrolytic cleavage by esterases, resulting in the free acid moiety BIBF 1202. Subsequently, BIBF 1202 is glucuronidated by various UGT enzymes, forming BIBF 1202 glucuronide. Metabolisation via CYP enzymes plays a minor role. After intravenous administration, nintedanib showed a high total plasma clearance (gMean: 1390 mL/min). Nintedanib is predominantly eliminated via metabolism and biliary/faecal excretion (about 94%). Renal excretion is a minor elimination pathway, both after intravenous and oral administration. The gMean terminal half-life of nintedanib was10 to 15h. Dose-proportional behaviour of C_{max} and AUC was concluded based on several studies in cancer patients investigating doses from 50 to 450 mg qd and from 150 to 300 mg b.i.d.

Based on *in vitro* investigations, relevant interactions of nintedanib with other drugs via the CYP enzyme system or via glucuronidation reactions are not expected. Transporter profiling was performed for nintedanib and its 2 main metabolites. In general, any interactions with transporter substrates were considered unlikely. Nintedanib is a P-gp substrate. In the drug-drug interaction trial 1199.161, exposure to nintedanib in healthy volunteers increased by about 1.6- to 1.7-fold for AUC and by about 1.8-fold for C_{max} following concomitant administration with the potent P-gp inhibitor ketoconazole. Thus, if administered concomitantly with nintedanib, potent P-gp inhibitors (e.g. ketoconazole, erythromycin) may increase nintedanib exposure. After concomitant administration with the potent P-gp inducer rifampicin, exposure to nintedanib decreased to approximately 50% based on AUC and to approximately 60% based on C_{max}. Thus, as potent P-gp inducers (e.g. rifampicin, carbamazepine, phenytoin, St. John's Wort) may decrease the exposure to nintedanib.

Additional data on the PK of nintedanib in patients with hepatic impairment are available from a dedicated single dose trial in patient volunteers with hepatic impairment (Child Pugh category A and B) but otherwise healthy plus matched healthy controls. Exposure in study 1199.200 increased by approximately 2-fold in subjects with mild liver impairment defined as Child Pugh category A and by approximately 8-fold in subjects with moderate liver impairment defined as Child Pugh category B [c03149997].

For more details please refer to the investigator drug brochure and to the product information for nintedanib [c01632700-18 and current SmPC Nintedanib].

1.2.2 Docetaxel

Docetaxel is an approved drug for a number of malignant cancer diseases including monotherapy of locally advanced and/or metastatic NSCLC after first line therapy. Docetaxel acts by disrupting the microtubular network in cells that is essential for mitotic and interphase cellular functions. Docetaxel binds to tubulin and promotes the assembly of tubulin into stable microtubules while simultaneously inhibiting their disassembly. This leads to microtubule bundles without normal function and to the stabilisation of microtubules causing inhibition of mitosis in cells [R04-2230, R07-4086]. According to the SPC, treatment with docetaxel may be associated with neutrophil count decreased, febrile neutropenia, anaemia, thrombocytopenia, hypersensitivity reactions, anorexia, localised erythema of the extremities, fluid retention, pain, peripheral motor neuropathy and neurosensory symptoms (such as paresthesia, dysesthesia), asthenia, gastrointestinal AEs (such as nausea, stomatitis, vomiting, diarrhoea), constipation, hypotension, nail disorders, alopecia, myalgia, cardiovascular side effects (arrhythmia: no severe) and infusion site reactions. Elevated liver enzyme values are associated with an increased treatment-related mortality [R04-2230, R07-4086]. When docetaxel is administered at a standard dosage (75 mg/m² every 3 weeks), the majority of patients develop grade 3-4 neutropenia. Myelosuppression and its complications can be particularly problematic for older patients and for patients receiving palliative treatment, such as those with advanced NSCLC. Attempts to reduce the incidence of toxicity, particularly of febrile neutropenia, have resulted in the evaluation of weekly docetaxel regimens, which maintain dose intensity while altering the tolerability profile. Three phase III studies have compared 3-weekly to weekly dosing in relapsed NSCLC setting. None of the three studies showed significant changes in response or survival, although two trended toward favouring the 3-weekly [R08-2280; R08-2275] dosing while one showed a trend in favour of weekly dosing [R15-1184]. All three, however, showed decreased grade 3 and 4 neutropenia with the weekly schedule. Results from these three studies and two additional randomized phase II studies were compiled in a meta-analysis, which analysed data from 865 patients in total and found overall a non-significant difference in median survival (27.4 weeks in the 3-week dosing versus 26.1 weeks in the weekly dosing, $P = 0.245$), and a reduction of grade 3 and 4 neutropenia (18% versus 5%) and febrile neutropenia (6% versus <1%), (both $P < 0.001$) [R15-5085]. These results suggest that weekly dosing is an acceptable alternative, particularly in a patient at greater baseline risk for neutropenia. Indeed, results from numerous clinical trials demonstrate that weekly dosing of docetaxel dramatically reduces the incidence of myelosuppression. In fact, there are fewer acute toxicities and the dose-limiting

toxicities, namely fatigue and asthenia, appear to be cumulative. Therefore weekly docetaxel could be recommended as a feasible alternative second-line treatment option for patients with advanced non-small cell lung cancer. [R08-2275, R08-2280; R15-1184].

1.2.3 Combination of nintedanib and docetaxel

A phase I trial of nintedanib plus docetaxel (75 mg/m2) in Caucasian patients with hormone refractory prostate cancer established a MTD of 200 mg b.i.d. for nintedanib [U08-1273-02].

A multicentre, randomised, double-blind, phase III trial (1199.13 trial) to investigate the efficacy and safety of oral nintedanib plus docetaxel therapy compared to placebo plus docetaxel therapy in patients with locally advanced or metastatic NSCLC after failure of first line platinum-based chemotherapy showed that the combination of docetaxel and nintedanib can be safely administrated and demonstrated significant benefit in patients with NSCLC of adenocarcinoma histology [P13-13353, P13-13346].

Overall survival (OS) was increased in patients with adenocarcinoma histology who were treated with nintedanib + docetaxel (median overall survival 12.6 months [95% CI 10.6–15.1] *vs.* 10.3 months [95% CI 8.6–12.2]; HR 0.83 [95% CI 0.70–0.99], p=0.0359).

Nintedanib in combination with docetaxel is an effective second-line option for patients with advanced NSCLC previously treated with one line of platinum-based therapy, especially for patients with adenocarcinoma [P14-00479].

The safety profile of nintedanib plus docetaxel in the 1199.13 trial was generally manageable with dose reductions and supportive care. The most common AEs by preferred term (any CTCAE grade) reported for the adenocarcinoma patient population with an incidence of >20% and with imbalances of more than 10% between treatment arms were diarrhoea (43.4% nintedanib vs. 24.6% placebo); ALT increased (37.8% vs. 9.3%), AST increased (30.3% vs. 7.2%), and nausea (28.4% vs. 17.7%). Severe AEs by preferred term (CTCAE grade 3 or greater) reported in the adenocarcinoma patient population with an incidence of >1% were ALT increased (11.6% nintedanib vs. 0.9% placebo), AST increased (4.1% vs. 0.6%), decreased neutrophil count (36.3% vs. 34.8%), decreased white blood cell (WBC) count (19.7% vs. 18.3%), febrile neutropenia (7.2% vs. 4.5%), diarrhoea (6.3% vs. 3.6%), and asthenia (3.1% vs. 0.9%).

2. RATIONALE, OBJECTIVES, AND BENEFIT - RISK ASSESSMENT

2.1 RATIONALE FOR PERFORMING THE TRIAL

As described in Section 1.2.3, the pivotal trial 1199.13 showed that the combination of nintedanib given continuously at the dose of 200 mg b.i.d. and docetaxel administered at the dose of 75 mg/m² every 3 weeks demonstrated significant OS benefit in patients with NSCLC of adenocarcinoma. In this specific histological pathology, nintedanib is the first drug, which prolongs the overall survival (OS) statistically and clinically significantly with 1-year survival rate over 50% (nintedanib: 52.7%, placebo: 44.7%) in combination with second line standard therapy in the last 10 years.

There is a high frequency of myelosuppression occurring with docetaxel administered at 75 mg/m^2 every 3 weeks in combination with nintedanib and its complications can be problematic for some NSCLC patients. Weekly docetaxel administration, as shown in several studies, is associated with a significant lower rate of febrile neutropenia and of grade 3-4 hematologic toxicities. Therefore weekly docetaxel + nintedanib could be an alternative treatment option for patients with adenocarcinoma of the lung who failed a platinum based-chemotherapy to reduce the frequency of myelosuppression.

This Phase I, 3+3 design, open label dose escalation study will explore the feasibility of the combination of nintedanib and the weekly administration of docetaxel on day 1, 8 and 15 every 28 days at the dose of 35 mg/m² with the goal to reduce the incidence of toxicity, particularly of febrile neutropenia, with keeping at least the same efficacy.
The differences found in the incidence and severity of neutropenia and febrile neutropenia would also imply a possible reduction of the cost of supportive care.

2.2 TRIAL OBJECTIVES

The primary trial objective is to determine the MTD of nintedanib twice daily (b.i.d.) plus weekly docetaxel 35 mg/m^2 (day 1, day 8, day 15 of a 28-day cycle) in patients with locally advanced or metastatic lung adenocarcinoma after failure of platinum-based first line chemotherapy

The further objectives concern the safety of nintedanib at each dose level in association with weekly docetaxel, as well as the PK data of nintedanib when administered continuously with docetaxel treatment. In addition, the efficacy of this combination will be assessed.

2.3 BENEFIT - RISK ASSESSMENT

The pivotal trial 1199.13 showed that the combination of nintedanib and docetaxel demonstrated statistically significant prolongation of centrally assessed progression free survival (PFS) and showed a statistically significant improvement of OS in NSCLC patients

with adenocarcinoma histology. The safety profile of nintedanib in combination with docetaxel in the 1199.13 trial was generally manageable with dose reductions and supportive care. No new or unexpected risks have been identified by the combination of nintedanib and docetaxel beyond what is known for docetaxel and what has been documented for nintedanib. Most common adverse events (AEs) with imbalance of incidence (>10%) in nintedanib arm were gastrointestinal (GI) tract (such as nausea and diarrhoea) and liver enzymes (AST, ALT) increase, and the safety profile was generally manageable with dose reductions and supportive care.

The weekly docetaxel administration demonstrated a better tolerability which translated in less haematological toxicities as compared to the 3-weekly regimen [R08-2275].

The aim of this Phase I study is to determine the MTD of nintedanib given in combination with weekly docetaxel and to evaluate the safety profile of this association in the lung adenocarcinoma.

Although rare, a potential for drug-induced liver injury is under constant surveillance by sponsors and regulators. Therefore, if necessary, this study requires timely detection, evaluation, and follow-up of laboratory alterations of selected liver laboratory parameters to ensure patients safety.
The potential benefit of nintedanib in this combination therapy is anticipated to outweigh the risks.

3. DESCRIPTION OF DESIGN AND TRIAL POPULATION

3.1 OVERALL TRIAL DESIGN AND PLAN

This is a phase I, open-label safety trial to determine the MTD of nintedanib plus docetaxel 35 mg/m2 given at Day1, Day8 and Day15 (28-day cycle) in patients with locally advanced or metastatic lung adenocarcinoma after failure of first line platinum-based chemotherapy.

Recruitment into this Phase I trial will follow a 3 + 3 design [R07-0220]. Four trial sites (two trial sites in France and two trial sites in Germany) with Phase I experience will be included to establish the MTD for nintedanib with docetaxel following a weekly schedule. In this trial, approximately 30 patients will be included. A maximum of 6 evaluable patients per cohort are required to determine the MTD of nintedanib in combination with weekly docetaxel. Patients who meet the inclusion criteria and none of the exclusion criteria and who have given their informed consent will be entered to the trial.

Inclusion of a new patient and the actual dose level will be agreed upon between the investigator and BI as the sponsor, in close interaction with the investigators.

The starting dose level of nintedanib administered continuously except the days of docetaxel infusion (see Section 3.2) will be 150 mg b.i.d, and, provided tolerability is acceptable, will increase to 200 mg b.i.d. Dose-escalation of nintedanib will be performed after discussion and agreement of the sponsor (BI) and the investigators of the trial. In case of unacceptable toxicity of the starting dose level, the dose of nintedanib will be de-escalated to 100 mg b.i.d to determine whether this dose is the MTD.

If the dose of nintedanib of 200 mg b.i.d administered continuously except the days of docetaxel infusion is tolerable an additional cohort will explore the administration of nintedanib 200 mg b.i.d continuously without interruption on the days of docetaxel infusion.

Only if the nintedanib dose reaches 200 mg b.i.d (with or without interruption on the days of docetaxel administration), it is planned to enrol an expansion cohort of 6 patients to better characterize the safety at the 200 mg b.i.d (MTD dose).

Patients will be treated with a combination therapy of docetaxel at the dose of 35 mg/m^2 on days 1, 8 and 15 followed by 2 weeks of rest every 28 days, and with nintedanib as long as they do not meet one of the treatment discontinuation criteria, e.g. progressive disease (PD), withdrawal of informed consent and undue toxicity (see Section 3.3.4.1).The trial will end once the last patient has completed the EOR visit foreseen 28 days (+ 7 days) after termination of treatment, or, once the last patient has been withdrawn from the trial and no further EOR visit is expected.

The database lock of this trial would be triggered by the date when the last patient reaches the end of trial participation.

Details of statistical considerations are given in section 7.

Pharmacokinetics (PK) of nintedanib will be evaluated only in patients treated with nintedanib 200 mg b.i.d given continuously without interruption on the days of docetaxel infusion. The pharmacokinetics in patients treated with nintedanib continuously except the days of docetaxel infusion will not be done.

Nintedanib and Docetaxel will be provided by the Sponsor directly. The trial drugs have to be stored according to the storage conditions.

3.1.1 Administrative structure of the trial

The trial is sponsored by Boehringer Ingelheim

Boehringer Ingelheim will appoint a Trial Clinical Monitor, responsible for coordinating the activities required in order to manage the trial in accordance with applicable regulations and internal SOPs, directing the clinical trial team in the preparation, conduct, and reporting of the trial, ordering the materials as needed for the trial, ensuring appropriate training and information of Local Clinical Monitors (CML), Clinical Research Associate (CRA), and investigators.

Data management, statistical and PK evaluations will be done by BI and a CRO according to BI SOPs. For these activities, a Trial Data Manager (TDM), a Trial Statistician (TSTAT) and a Trial Clinical Pharmacokineticist (TCPK) will be appointed. Tasks and functions assigned in order to organise, manage, and evaluate the trial will be defined according to BI SOPs. A list of responsible persons and relevant local information can be found in the Investigator Site File (ISF) and Trial Master File (TMF).
Investigators of participating sites and BI study clinical monitor and representatives will be appointed to evaluate DLT, overall safety and other available data (e.g. PK data, if available) and confirm dose escalation steps.
The organisation and on-site monitoring of the trial will be performed by Boehringer Ingelheim.

All trial relevant documentation will be stored in BI's TMF. Trial relevant documentation which has to be at the trial site will be filed in the ISF at the investigator site.

A co-ordinating investigator will be nominated by BI to coordinate investigators at different sites participating in this trial, and will sign the clinical trial report of this trial, and has experience in this type of trial and investigations. Tasks and responsibilities for the co-ordinating investigator will be defined in a contract filed before initiation of the trial.

This trial will be conducted by expert investigators in oncology Phase I and also chemotherapy of NSCLC at appropriate study sites where a very cautious safety control procedure has been established. There will be no steering committee or data monitoring committee for this trial.

Data management, statistical and pharmacokinetic analyses will be performed by Boehringer Ingelheim or a CRO authorized by Boehringer Ingelheim.

The safety laboratory investigations (haematology, biochemistry, coagulation parameters, urine analysis) will be performed locally at the sites. The certification for each local laboratory must be provided and filed at the sponsor and the local ISF of the site.

Interactive response technology (IRT) will be provided by a CRO/vendor. Details will be provided in the IRT manual available in the ISF.

An ISF containing all relevant study related documentation will be maintained according to European regulations and BI SOPs at each study site. A copy of the ISF documents will also be kept as an electronic TMF at BI according to BI SOPs. Documents related to participating investigators and other important participants, especially their curricula vitae, will be filed in the TMF.

3.2 DISCUSSION OF TRIAL DESIGN, INCLUDING THE CHOICE OF CONTROL GROUP(S)

In this open label Phase I trial, the dose escalation will follow a 3 + 3 design to evaluate the MTD of nintedanib in combination with weekly docetaxel (Day 1, Day 8 and Day 15 in 28-day cycles). The safety profile of the patients will be evaluated at each dose level of Nintedanib in combination with weekly docetaxel.

The dose levels of nintedanib will be as follows:

- Nintedanib will be administered continuously except the days of docetaxel infusion:
  - Level -1 (de-escalation dose): nintedanib 100 mg b.i.d in combination with docetaxel.
  - Level 1 (starting dose): nintedanib 150 mg b.i.d in combination with docetaxel
  - Level 2 (escalation dose): nintedanib 200 mg b.i.d in combination with docetaxel
- Nintedanib will be administered continuously without interruption on the days of docetaxel infusion:
  - Level 3 nintedanib 200 mg b.i.d in combination with docetaxel.

3.3 SELECTION OF TRIAL POPULATION

A log of all patients included into the study (i.e. having given informed consent) will be maintained in the ISF at the investigational site.
Approximately 30 entered patients will participate to this trial which will be conducted in 2 sites in France and in 2 sites in Germany.

3.3.1 Main diagnosis for study entry

Patients with locally advanced or metastatic lung adenocarcinoma after failure of first line platinum based chemotherapy.

3.3.2 Inclusion criteria

1. Patients aged 18 or older at the date of informed consent
2. Patients with histologically/cytologically locally advanced (Stage IIIB) or metastatic (Stage IV) lung adenocarcinoma after failure of first line platinum-based chemotherapy (patients with non-target lesion only are eligible)
   First line chemotherapy may include continuation or switch maintenance therapy.

   One prior adjuvant and/or neoadjuvant chemotherapy line is accepted. Prior immunotherapy is allowed.
3. Patients who have life expectancy of at least 3 months
4. Patients who are Eastern Cooperative Oncology Group (ECOG) performance status (PS) ≤1 at screening
5. Written informed consent that is consistent with ICH-GCP guidelines and local legislation.

3.3.3 Exclusion criteria

1. Patients who have received more than one prior line of chemotherapy (i.e., second or third line chemotherapy) for advanced or metastatic NSCLC.
2. Patients known to be positive for activating Epidermal Growth Factor Receptor (EGFR) mutation or patients known to be positive for ALK translocation.
3. Patients who have received previous therapy with other vascular endothelial growth factor (VEGF) or vascular endothelial growth factor receptor (VEGFR) inhibitors (other than bevacizumab) for the treatment of NSCLC at any time.
4. Patients who have received following treatments within 4 weeks prior to start of study therapy:
   a) Other investigational drugs
   b) Chemo-, hormone-, immunotherapy, or monoclonal antibody.
5. Patients who have received radiotherapy within the 4 weeks (in the case of limited -field [e.g. brain or bone metastasis]), radiotherapy with palliative intent, within 2 weeks) prior to start of study therapy
6. Persistence at screening of clinically relevant therapy related toxicities from previous chemotherapy and/or radiotherapy (AE from previous treatment ≥ Grade 2)
7. Patients with one or more of the following laboratory values at screening:

a) Serum creatinine>1.5 x upper limit of normal (ULN)

b) Proteinuria ≥CTCAE grade 2

c) Total bilirubin>1.0 x ULN

d) ALT and/or AST ≥ 1.5 x ULN for patients without liver metastases. ALT and/or AST ≥ 2.5 x ULN for patients with liver metastases.

e) Alkaline phosphatase (ALP)>2.5x ULN

f) Prothrombin time and/or partial thromboplastin time>50% deviation from normal limits

g) International normalised prothrombin ratio (INR)> 3

h) Absolute neutrophil count (ANC) < 1500/mm^3

i Platelets<100000/mm^3

j) Haemoglobin< 9 g/dL

8. Patients with any of the following conditions at screening:

a) Ascites and/or uncontrolled pleural effusion

b) Significant weight loss (>10%) within the past 4 weeks prior to registration

c) Current peripheral neuropathy ≥ CTCAE grade 2 except due to trauma

d) Major injuries and/or surgery within the past 4 weeks prior to start of study therapy

e) Diabetes mellitus not controlled by medical therapy

f) Contraindication to high dose corticosteroid therapy

g) Active or chronic hepatitis C (HCV) and/or B (HBV) infection and/or known human immunodeficiency virus (HIV) infection

h) Known or suspected active alcohol or drug abuse

i) Serious illness or concomitant non-oncological disease such as neurologic, psychiatric, infectious disease or active ulcers (gastrointestinal malabsorption or other conditions, skin), or history of hollow-organ perforation

j) History of laboratory abnormality that may increase the risk associated with study participation or study drug administration

k) Other malignancy within the past 3 years other than basal cell skin cancer, or carcinoma in situ of the cervix

l) Active brain metastases or leptomeningeal disease stable for <4 weeks (e.g. no previous adequate treatment with radiotherapy, symptomatic, requiring treatment with anti-convulsants). If brain metastases have been treated, they must be stable for ≥4 weeks. Dexamethasone therapy will be allowed if administered at a stable dose for at least 4 weeks prior to registration. Patients with newly diagnosed brain metastases will be excluded from the study until/unless treated as described above

m) Radiographic evidence (CT or MRI) of cavitary or necrotic tumours or centrally located tumours with local invasion of major blood vessels

n) Clinically significant haemoptysis within the past 3 months (more than five ml)

o) Bleeding or thrombotic disorders requiring oral anticoagulant therapy (treatment with low molecular weight heparin and/or heparin flush as needed for maintenance of an indwelling intravenous device is allowed) or anti-platelet therapy (except for chronic low-dose therapy with acetylsalicylic acid ≤325 mg per day).

p) History of major thrombotic event or known inherited predisposition to thrombosis, or clinically relevant major bleeding event in the past 6 months

q) Significant cardiovascular diseases (i.e. hypertension not controlled by medical therapy, unstable angina, history of myocardial infarction within the past 6 months, congestive heart failure New York Heart Association (NYHA)>II, serious cardiac arrhythmia, pericardial effusion

9. Male or female patients of childbearing potential who are sexually active and unwilling to use a highly effective method of contraception (e.g. contraceptive implants, injectables, combined oral contraceptives, some intrauterine devices or vasectomised partner for participating females, a double barrier method such as condom plus diaphragm with spermicide for participating males) during the trial and for at least 3 months after the end of the treatment with nintedanib and for at least 6 months after the end of the therapy with docetaxel. Patients will be considered to be of childbearing potential unless surgically sterilised by hysterectomy or bilateral tubal ligation/salpingectomy, or post-menopausal for at least 1 year

10. Patients who are pregnant or breast feeding at screening

11. Any contraindications for therapy with docetaxel or history of severe hypersensitivity reactions to docetaxel or other drugs formulated with polysorbate 80 (Tween 80).

12 Hypersensitivity to nintedanib and/or the excipients of the trial drugs.

13. Known allergy to soya and/or peanut proteins. Dietary soya products are known to cause allergic reactions including severe anaphylaxis in persons with soya allergy. Patients with known allergy to peanut proteins carry an enhanced risk for severe reactions to soya preparations.

14. Patients unable to comply with the study protocol for any reason

15 Patients judged inappropriate for entry into the study by the investigator

3.3.4 **Removal of patients from therapy or assessments**

3.3.4.1 Removal of individual patients:

A patient will discontinue study treatment (nintedanib +/- docetaxel) if any one of the following applies:

- Progressive disease.

  If a patient experiences a radiological progression according to RECIST 1.1 but derive an obvious clinical benefit from the treatment according to the investigator's judgement, the patient may remain on study after discussion with the sponsor. The first date of progression will be documented as the date of the progression of the disease in the eCRF.
- Withdrawal of informed consent for active treatment.
- Patient's request.
- The patient is no longer able to participate in the study (e.g. AE, major surgery, pregnancy, concomitant diagnoses, concomitant therapies; if the patient is no longer able to attend study visits due to worsening of disease, or for administrative reasons).
- Significant deviation from the protocol or eligibility criteria; the patient receives prohibited concomitant medication (see Section 4.2.2).
- The patient cannot complete the docetaxel infusion in treatment Cycle 1 due to hypersensitivity reactions etc.

Patients who discontinue only one drug of the combination (e.g., either docetaxel or nintedanib) but qualify to receive monotherapy with the remaining drug are not considered to have ended active treatment.

Reasons for discontinuation will be documented in the electronic case report form (eCRF).

3.3.4.2 Discontinuation of the trial by the sponsor

Boehringer Ingelheim reserves the right to discontinue the trial overall or at a particular trial site at any time for the following reasons:

1. Failure to meet expected enrolment goals overall or at a particular trial site.
2. Emergence of any efficacy/safety information invalidating the earlier positive benefit-risk assessment that could significantly affect continuation of the trial.
3. Violation of GCP, the CTP, or the contract by a trial site or investigator, disturbing the appropriate conduct of the trial.

The investigator / the trial site will be reimbursed for reasonable expenses incurred in case of trial termination (except in case of the third reason).

3.3.4.3 Patient replacement

Patients who meet one of following in Cycle 1 have to be replaced for the DLT evaluation:

1. Patient cannot complete the docetaxel infusion due to hypersensitivity reactions.
2. Patient cannot complete treatment cycle due to other reasons than AEs related to study treatment (i.e. nintedanib and/or docetaxel) such as progressive disease, withdrawn informed consent, non-related AE with CTCAE grade 3 or greater or administrative reason.
3. Missed dose of nintedanib for more than 14 doses (non-compliance) during treatment cycle 1 because of the reasons other than drug related AEs.
4. Patient missed the 2 subsequent docetaxel infusions (Day 8 and Day 15) during cycle 1.

Replacement of patients will be determined individually after discussion between the investigator and the sponsor.

4. TREATMENTS

4.1 TREATMENTS TO BE ADMINISTERED

Patients will be treated in cycles of 28 days duration. During a combination treatment cycle, docetaxel will be administered on Day 1, on Day 8, on Day 15 followed by 2 weeks of rest and nintedanib will be administered twice daily. If patients discontinue docetaxel and meet the retreatment criteria for nintedanib (see Section 4.1.4.1.4) and do not meet one of the discontinuation criteria (see Section 3.3.4.1), patients on nintedanib monotherapy will start therapy on Day 1 of each cycle until Day 28.

4.1.1 Identity of BI investigational product(s) and combination product(s)

4.1.1.1 Nintedanib (investigational medical product)

Table 4.1.1.1: 1 Nintedanib

Substance (INN):	Nintedanib
Pharmaceutical formulation:	Soft gelatine capsule
Pharmaceutical code	BIBF 1120
Source:	Boehringer Ingelheim Pharma GmbH & Co. KG
Unit strength:	100 mg and 150 mg capsules
Route of administration:	Oral
Posology:	See Section 3.2 (100 mg b.i.d or 150 mg b.i.d or 200 mg b.i.d).<br><br>Twice daily (to be swallowed unchewed with a glass of water of about 250 mL with a dose interval of <u>12 hours</u> at the same time every day, usually in the morning and the evening <u>after</u> food intake).<br><br>See Dose reduction according to Section 4.1.4.2.

Table 4.1.1.1: 2 Docetaxel (investigational medicinal product)

Substance:	Docetaxel
Pharmaceutical form:	Injection concentrate
Source:	Sanofi
Unit strength:	Docetaxel 160 mg / 8 ml and 80 mg/4ml
Posology:	At Day 1, at Day 8 and at Day 15 every 28 days cycle
Premedication	Corticosteroid, such as dexamethasone (8mg twice daily orally the day before and after docetaxel infusion; 8 mg intravenously 30 minutes before docetaxel). Antiemetics are recommended before docetaxel infusion.
Route of administration:	Intravenous infusion over 60 minutes
Infusion dose	35 mg/m²
Duration of use:	Weekly until discontinuation criteria (see Sections 3.3.4.1 and 4.1.4.1.2 are fulfilled).

4.1.2 **Method of assigning patients to treatment groups**

As the 1199.224 study is designed to determine the MTD of nintedanib in combination with weekly docetaxel, it is planned to initially start with 150 mg nintedanib b.i.d.

Patients who meet the eligibility criteria and who have given their written informed consent will be entered into the study.
Registration of the patients will be performed following RDC. As there are no predefined cohorts, the IRT system can assign nintedanib and docetaxel kit numbers.
Details are described in the IRT manual available in the Investigator Site File (ISF).

The decision regarding the opening of another cohort of 3 patients (e.g. for escalation/de-escalation of the starting dose) will only be made after discussion between the BI Clinical Monitor and the Investigators of the trial. Depending on the occurrence of drug-related DLT within treatment course 1 in a three or six patient cohort, the Investigators and the BI Clinical Monitor will decide the next dose cohort after discussion of the safety data available at that time (including safety data available also for repeated treatment courses).

Table 4.1.2: 1 Rules for dose escalation

**Number of Patients with DLT at a Given Dose Level**	**Escalation Decision Rule**
0 out of 3 or 1 out of 6	Enter 3 patients at the next higher dose level
≥ 2 out of 3	Dose escalation will be stopped. Three (3) additional patients will be entered at the next lowest dose level if only 3 patients were treated previously at that dose level.<br><br>If 6 patients were already treated at that next lowest dose level, no more patients will be entered and this dose will be considered the MTD.
1 out of 3	Enter 3 more patients at this dose level.<br>If 0 of these 3 patients experience DLT, proceed to the next dose level (if highest dose, this dose will be considered as the MTD).<br>If 1 or more of this group suffer DLT, then dose escalation is stopped. Three (3) additional patients will be entered at the next lowest dose level (see above).
≤ 1 out of 6 at highest dose level	This is the MTD and recommended dose.

The MTD nintedanib in combination with weekly docetaxel 35 mg/m² will be defined as the highest dose of nintedanib at which no more than 1 out of 6 patients have a DLT within treatment course 1. The highest dose is capped to 200 mg bid given without interruption even on days of docetaxel infusion; if there is no DLT at this dose level then this would be considered as the MTD.

Expansion cohort:

Only if the nintedanib dose reaches 200 mg b.i.d (with or without interruption on the day of docetaxel administration), it is planned to enrol an expansion cohort of 6 patients to better characterize the safety at the 200 mg b.i.d (MTD dose).

4.1.3 **Selection of doses in the trial**

4.1.3.1 General recommendations for dose selection in the present trial

4.1.3.1.1 Nintedanib

The registered dose of nintedanib in combination with 75 mg/m2 of docetaxel is 200 mg b.i.d.in Caucasians [U08-1273-02].

The purpose of this study is to assess the MTD of nintedanib following a 3+3 design (orally, 100 mg b.i.d, 150 mg b.i.d or 200 mg b.i.d) in combination with weekly docetaxel (intravenously, 35 mg/m² at day 1, at day 8 and at Day 15, a 28 day cycle) in lung adenocarcinoma. The combination of nintedanib at the MTD with weekly docetaxel should have the same efficacy with a better tolerability than nintedanib with standard 3-weekly docetaxel 75 mg/m².

4.1.3.1.2 Docetaxel

Docetaxel monotherapy is approved for the treatment of patients with NSCLC previously treated with one prior platinum-based chemotherapy. The recommended dose of docetaxel for the second line treatment of patients with NSCLC is 75 mg/m2 in global as an intravenous infusion every 3 weeks.

Weekly scheduling of docetaxel (35 mg/m² Day 1, Day 8 and Day 15, following a 28 day cycle) has been tested and compared to the 3 weekly schedule of docetaxel which showed comparable efficacy and less myelotoxicity in pretreated NSCLC patients. In particular, there were significantly lower rates of grade 3 to 4 anaemia, leukopenia and neutropenia. Febrile neutropenia was observed at a significantly lower rate as well. With less myelotoxicity, the weekly schedule of docetaxel seems to be a good alternative for patients at risk of severe neutropenia.

A weekly dose of 35 mg/m2 will be used as the fixed dose for all patients enrolled in this trial. No dose adjustments will be made and if necessary, docetaxel will not be administered as defined in Section 4.1.4.1. Docetaxel could be stopped after 8 cycles if there is no further clinical benefit is expected in the opinion of the investigator.

As indicated, all patients will receive premedication for docetaxel (such as dexamethasone and antiemetics) prior to the docetaxel infusion. Premedication for docetaxel will be captured in the electronic case report form (eCRF [e.g. trade name or substance name, dose and treatment duration]).

Patients should be observed closely for hypersensitivity reactions especially during the first and second infusion of docetaxel. Hypersensitivity reactions may occur within a few minutes following the initiation of the infusion of docetaxel. Thus, facilities for the treatment of hypotension and bronchospasm should be available. If hypersensitivity reactions occur, minor symptoms such as flushing or localised cutaneous reactions do not require interruption of

therapy. However, severe reactions, such as severe hypotension, bronchospasm or generalised rash/erythema require immediate discontinuation of docetaxel and appropriate therapy. Patients who have developed severe hypersensitivity reactions should not be re-challenged with docetaxel (see Section 4.1.4.1.2).

4.1.4 Drug assignment and administration of doses for each patient

Docetaxel will be administered by intravenous infusion approximately over 60 minutes on Day 1, on Day 8, on Day 15 of each 28 days treatment cycle.

The investigator or his/her assignee will dispense nintedanib trial medication to patients, as described in Section 4.1.6.

Nintedanib will be taken (swallowed) whole, unchewed twice daily after a meal every 12 hours, at approximately the same time e.g. in the morning and in the evening, starting with the morning dose on the day following the day of docetaxel administration (from Day 2 to Day 7, from Day 9 to Day 14; from Day 16 to Day 28 for each cycle). Nintedanib will not be taken on the days of docetaxel administration in dose levels 1; 2 and -1. Nintedanib will be taken without interruption in dose level 3, even on days of docetaxel administration.

Patients who are treated with continuous nintedanib in association with docetaxel (cohort level 3) are also required to visit the study site without taking the study drug for morning dose on the visit day when pharmacokinetic blood samples are planned at Cycle 1 Day 8 and Day 15 and at Cycle 2 Day 1, Day 8 and Day 15.

If a patient misses one or more doses of nintedanib, he/she should not replace the missing dose but take the subsequent doses according to protocol.

If a patient experiences adverse events (AEs) that would require temporary interruption of study treatment (nintedanib and / or docetaxel) as follows (see Section 4.1.4.1 and 4.1.4.2):
- No administration followed by dose reduction of nintedanib.
 and/or
- No administration of docetaxel until the next infusion.
The treatment may be resumed when the retreatment criteria are met.

4.1.4.1 Treatment cycle intervals

Treatment cycles should be repeated every 28 days. In case of retreatment criteria with docetaxel are not met, docetaxel should be administered at the same dosage at the next planned administration. If docetaxel administration was missed due to related AE for two or more consecutive infusions, docetaxel treatment should be discontinued. Docetaxel could be stopped after 8 cycles if there is no further clinical benefit is expected in the opinion of the investigator.

4.1.4.1.1 Treatment criteria for docetaxel therapy

Treatment criteria for docetaxel are as follows:

1. Absolute neutrophil count (ANC) ≥ 1500/mm^3 before Day 1 of docetaxel administration and ANC ≥ 1000 / mm^3 before Day 8 and Day 15 of docetaxel administration.
2. Thrombocyte count ≥ 100000/mm^3 before each day of docetaxel administration
3. Total bilirubin values within normal ranges
4. ALT and/or AST ≤ 1.5xULN and concomitant with alkaline phosphatases ≤ 2.5 x ULN
5. No severe hypersensitivity reaction to docetaxel in a previous treatment cycle
6. Peripheral neuropathy CTCAE grade ≤ 1.
7. Non-hematologic toxicity has recovered to baseline or to CTCAE grade 1 or less (excluding CTCAE grade 2 alopecia)
8. Recovery of docetaxel related AEs to Grade ≤ 1.

In case patients do not meet the treatment criteria for docetaxel therapy, please skip docetaxel infusion and postpone the administration to the next planned administration. During this period, therapy with nintedanib shall continue if none of the discontinuation criteria related to nintedanib is met.

In case of discontinuation of nintedanib for other reasons than progressive disease (e.g. toxicity), the treatment with docetaxel monotherapy is allowed.

4.1.4.1.2 Stopping criteria for docetaxel therapy

In case one of the following criteria would apply, docetaxel therapy has to be stopped:

1. CTCAE grade 3 or greater peripheral neuropathy
2. Severe hypersensitivity to docetaxel
3. Omission of 2 or more consecutive docetaxel infusions.

In this event patients may continue monotherapy with nintedanib as long as none of the discontinuation criteria related to nintedanib treatment are met (see Section 3.3.4.1 and Section 4.1.4.2).

4.1.4.1.3 Temporary treatment interruption of nintedanib treatment

Treatment with nintedanib will be temporarily discontinued and adequate therapy be initiated if one of the following drug related AEs would occur:

1. CTCAE grade 3 or greater non haematological drug related AEs.
2. CTCAE grade 3 or greater diarrhoea.
3. CTCAE grade 2 diarrhoea for more than 7 days despite of supportive care.
4. CTCAE grade 3 or greater nausea despite supportive care.

5. CTCAE grade 2 nausea for more than 7 days despite of supportive therapy
6. CTCAE grade 2 or greater vomiting despite of supportive care.
7. An increase of ALT and/or AST to CTCAE grade 3 or greater.
8. An increase of ALT and/or AST to CTCAE grade 2 or greater in conjunction with CTCAE grade 1 or greater total bilirubin increase.

Treatment with nintedanib can be temporarily discontinued because of reasons other than above by investigator's judgement.

4.1.4.1.4 Retreatment criteria for nintedanib

Treatment with nintedanib may be resumed at a dose level described in Section 4.1.4.2 as long as the events causing temporary treatment interruption have recovered to baseline, or to CTCAE grade 1 (except CTCAE grade 2 alopecia). If the AE is not related to nintedanib following the investigator judgement, dose reduction is not needed.

In case of interruption of retreatment for more than 21 days would be required by the investigator or the patient this needs to be discussed and agreed between the investigator and the sponsor.

4.1.4.2 Dose reduction scheme of nintedanib (given in combination or as monotherapy)

In case of nintedanib related AEs as outlined in this section, the dose of nintedanib will be reduced after a pause.

- For the patients treated with 200 mg b.i.d of nintedanib: nintedanib will be reduced from 200 mg b.i.d. to 150 mg b.i.d. for the first dose reduction and from 150 mg b.i.d. to 100 mg b.i.d. for the second dose reduction when a dose reduction is considered necessary.
- For the patients treated with 150 mg b.i.d of nintedanib: nintedanib will be reduced from 150 mg b.i.d to 100 mg b.i.d for the first reduction dose. No dose reduction is allowed below 100 mg b.i.d.

After a pause followed by a dose reduction if the AE is drug related for nintedanib, it is not allowed to increase the dosage of nintedanib.

Dose reduction and administration schemes according to the occurrence of drug related AEs are presented in the following. (Tables 4.1.4.2.1-4)

Table 4.1.4.2: 1 Dose reduction (nintedanib) for non-haematological AEs except for diarrhoea, nausea, vomiting, isolated gamma-glutamyl transferase, AST and/or ALT increases

AE	Nintedanib
CTCAE grade 3 or greater non haematological AEs	Interruption of the treatment until Recovery (CTCAE grade ≤ 1 or baseline).<br>If drug related, restart with reduced dose.

Table 4.1.4.2: 2 Dose reduction (nintedanib) for CTCAE grade 3 or greater AST and/or ALT increases, or for CTCAE Grade 2 or greater AST and/or ALT increases in conjunction with CTCAE grade 1 or greater total bilirubin

AE	Nintedanib
1st episode	Interruption of the treatment until Recovery (CTCAE grade ≤ 1 or baseline).<br>If drug related, restart with reduced dose.
2nd episode	Interruption of the treatment until Recovery (CTCAE grade ≤ 1 or baseline).<br>If drug related, restart with reduced dose.
3rd episode	Permanent discontinuation

Table 4.1.4.2: 3 Dose reduction (nintedanib) for CTCAE grade 2 or greater vomiting or CTCAE grade 3 or greater nausea despite supportive care[1].

AE	Nintedanib
1st episode	Interruption of the treatment until Recovery (CTCAE grade ≤ 1 or baseline). If drug related, restart with reduced dose.
2nd episode	Interruption of the treatment until Recovery (CTCAE grade ≤ 1 or baseline). If drug related, restart with reduced dose.
3rd episode	Permanent discontinuation

[1] Prophylactic anti-emetic therapy is mandatory for the subsequent treatment cycles. See Section 4.2.1 for recommended therapies.

Table 4.1.4.2: 4 CTCAE grade 2 diarrhoea for more than 7 consecutive days despite supportive care[1] or CTCAE grade 3 diarrhoea.

AE	Nintedanib
1st episode	Interruption of the treatment until Recovery (CTCAE grade ≤ 1 or baseline). If drug related, restart with reduced dose.
2nd episode	Interruption of the treatment until Recovery (CTCAE grade ≤ 1 or baseline). If drug related, restart with reduced dose.
3rd episode	Permanent discontinuation

[1] See Section 4.2.1 for recommended therapies.

4.1.5 Blinding and procedures for unblinding

Not applicable.

4.1.6 Packaging, labelling, and re-supply

Nintedanib and Docetaxel will be provided by BI.

Nintedanib soft gelatine capsules will be packaged in aluminium-blister sheets.

Nintedanib will be delivered in medication kits. Kits will be labelled according to French and German requirements.
Docetaxel will be also labelled according to French and German requirements.

The IRT system ensures that sufficient medication is allocated to a respective patient for daily treatment.

Patients will be handed out the nintedanib trial medication for the upcoming treatment cycle during the planned and/or regular visit on Day 1 of each treatment cycle. In case that a patient is expected to visit the study site more than 28 days after the Day 1, additional medication should be handed out during previous visit. The unused trial drug with the previous cycle has to be collected at the Day 1 of each cycle.

In case a patient interrupts intake of nintedanib during a treatment cycle and meets the criteria for retreatment within the same treatment cycle with reduced dose, the reduced dose should be handed out during the visit by the investigator or a deputy. Nintedanib with the previous dose not used until then has to be collected.

See Section 4.1.8 for drug accountability.

For details of packaging and the description of the label, see ISF.

4.1.7 Storage conditions

Nintedanib has to be stored in the original package according to the storage instructions as provided on the label and in the ISF at a location with limited access to authorised people.

Docetaxel has to be stored according to the manufacturer’s storage instructions. Docetaxel 160 mg or 80 mg concentrate for solution i.v. should not be stored at a temperature above 25 °C. It should be stored in the original container to protect from light. The vials are for single use and must be used immediately after their opening. If not used immediately, in-use storage times and conditions are the responsibility of the use and would normally not be longer than 6 hours below 25°C including the one hour infusion.
Docetaxel 160 mg or 80 mg infusion solution is supersaturated therefore may crystallize over time. If crystals appear the solution must no longer be used and shall be discarded.

Nintedanib and docetaxel may only be dispensed to trial patients according to the protocol by authorised personnel as documented in the form “Investigator’s Trial Staff”.

Temperature logs must be kept. In case temperature would be out of range, this would have to be reported in the ISF and the sponsor be notified.

4.1.8 Drug accountability

Nintedanib and Docetaxel will be provided by the sponsor.

The pharmacist will receive the investigational drugs delivered by the sponsor when the following requirements are fulfilled:

- Approval of the study protocol by the institutional review board (IRB).
- Availability of a signed and dated clinical trial contract between the sponsor and the site.
- Approval/notification of the regulatory authority, e.g. competent authority.
- Availability of the curriculum vitae of the principal investigator.
- Availability of a signed and dated clinical trial protocol (in exceptional cases, medication could already be sent to the site, before its activation via IWRS).

The pharmacist must maintain records of the product's delivery to the trial site, the inventory at the site, the use by each patient, and the return to the sponsor or alternative disposition of unused product(s).

These records will include dates, quantities, batch/serial numbers, expiry ('use by') dates, and the unique code numbers assigned to the investigational product(s) and trial patients. The pharmacist will maintain records that document adequately that the patients were provided the doses specified by the CTP and reconcile all investigational product(s) i.e., nintedanib received from the sponsor.

At the time of return to the sponsor and/or appointed CRO, the pharmacist must verify that all unused or partially used nintedanib supplies have been returned by the clinical trial patient and that no remaining supplies are in the investigator's possession. Unused or used nintedanib medication will be sent back to the sponsor or to an appointed CRO and will be destroyed. A destruction certificate will be required. The used or unused docetaxel medication will be destroyed at each site with a destruction certificate.

4.2 CONCOMITANT THERAPY, RESTRICTIONS, AND RESCUE TREATMENT

4.2.1 Rescue medication, emergency procedures, and additional treatment(s)

An antidote against nintedanib is not available. Potential side effects of nintedanib and/or docetaxel have to be treated symptomatically. Treatment of symptomatic side effects or tumour-associated symptoms is allowed (except for therapies listed in Section 4.2.2).

Nausea and/or vomiting should be treated according to the international guidelines and recommendations (Multinational Association of Supportive Care in Cancer [R06-0986]). Anti-emetic therapy with e.g. glucocorticoids, metoclopramide or dimenhydrinat may be initiated as clinically indicated (CTCAE grade 2 or greater nausea and CTCAE grade 1 or greater vomiting), with adequate hydration. If other anti-emetic therapy is not effective (CTCAE grade 2 or greater vomiting and for CTCAE grade 3 or greater nausea), short-term treatment with a 5-HT3 antagonist (e.g. granisetron) and concomitant steroid (e.g. dexamethasone) may be indicated. Patients known to suffer from anticipatory vomiting and patients who experienced nausea and/or vomiting should receive prophylactic anti-emetic therapy prior to any further treatment cycle with docetaxel. In the event of dehydration administration of electrolytes and fluids is required. Plasma levels of electrolytes should be monitored, if moderate or severe (CTCAE grade 2 or greater) gastrointestinal AEs occur.

Diarrhoea (CTCAE grade 2 or greater) should be treated at first signs with adequate hydration and anti-diarrhoeal medicinal products, e.g. loperamide should be administered according to the recommended dose schedule: i.e., 4 mg of loperamide at onset followed by 2 mg after every stool until diarrhoea has abated for 12 hours (maximal daily dosage 12-16 mg).
All patients will receive oral corticosteroids as pre- and concomitant medication for docetaxel (such as dexamethasone) for three days starting one day prior to the docetaxel infusion.

Patients experiencing neutrophil count decreased may receive granulocyte colony stimulating factor (G-CSF) at investigator’s discretion.

4.2.2 Restrictions

4.2.2.1 Restrictions regarding concomitant treatment

Additional chemo-, immuno-, hormone- or radiotherapy is not allowed during the active treatment period of this trial.

Concomitant medications, or therapy to provide adequate care, may be given as clinically necessary.

The use of bisphosphonates for treatment of bone metastases is allowed and Concomitant treatment with growth factors (e.g. filgrastim, pegfilgrastim) are allowed

For symptom control palliative radiotherapy may be permitted for bone metastases in extremities, hip or vertebrae after discussion with the BI clinical monitor, provided that radiotherapy does not affect the target lesions, and the reason for the radiotherapy does not reflect progressive disease (PD).
The oral anticoagulant therapy is not allowed (anticoagulant therapy with low molecular weight heparin and / or heparin flush may be allowed if clinically required for a patient to treat AEs). These patients will be supervised at close intervals until they reach stable anticoagulation situation.
Anti-platelets are forbidden except for chronic low-dose therapy with acetylsalicyclic acid ≤ 325 mg per day.

In vitro studies have shown that the metabolism of docetaxel may be modified by the concomitant administration of compounds that induce, inhibit, or are metabolised by cytochrome P450 (CYP) 3A4, such as cyclosporine, terfenadine, ketoconazole, erythromycin, nifedipine and troleandomycin. Based on in vitro findings caution should be exercised with these drugs when treating patients receiving docetaxel as there is a potential for a significant interaction (e.g. substantial increases or decrease in docetaxel blood concentrations, see product information).

In contrast, based on in vitro data, only a minor extent of biotransformation of nintedanib consisted of CYP pathways; nintedanib or its metabolites did not inhibit or induce CYP enzymes. Therefore, the likelihood of drug-drug interactions based on CYP metabolism with nintedanib is considered to be low.

P-glycoprotein (P-gp): nintedanib is a substrate of P-gp. If co-administered with nintedanib, potent P-gp inhibitors (e.g. ketoconazole or erythromycin) may increase exposure to nintedanib. In such cases, patients should be monitored closely for tolerability of nintedanib. Management of side effects may require interruption, dose reduction, or discontinuation of therapy with nintedanib. Potent P-gp inducers (e.g. rifampicin, carbamazepine, phenytoin, and St. John’s Wort) may decrease exposure to nintedanib. Co-administration with nintedanib should be carefully considered.

Treatment with other investigational drugs or treatment under another clinical trial is not allowed during the active treatment period.

4.2.2.2 Restrictions on diet and life style

Not applicable

4.3 TREATMENT COMPLIANCE

Docetaxel will be administered as an intravenous infusion in the hospital setting at the investigating sites under supervision of authorized personnel. Date of administration as well as a statement whether infusion was done according to protocol and/or whether infusion was interrupted will be recorded in the e-CRF.

Patients are requested to bring all remaining nintedanib medication including package material with them when attending visits.

Based on capsules counts, treatment compliance of nintedanib will be calculated as the number of capsules, divided by the number of capsules which should have been taken according to the scheduled period, multiplied by 100.

Treatment compliance (%) = Number of nintedanib capsules actually taken x 100 / Number of nintedanib capsules which should have been taken.

If the number of doses taken is not between 80-120%, site staff will explain to the patient the importance of treatment compliance.

5. VARIABLES AND THEIR ASSESSMENT

5.1 TRIAL ENDPOINTS

5.1.1 Primary Endpoint(s)

Determination of the maximum tolerated dose (MTD) of nintedanib in combination with docetaxel given in a weekly schedule and the number of patients with DLTs during the first treatment cycle is the primary endpoint. The MTD will be defined in view of dose-limiting toxicity (DLT) during the first course only. The MTD together with the safety profile will serve to determine the dose to be used in further development.

The MTD is defined as:

The Maximum Tolerated Dose (MTD) is defined as the highest dose studied at which not more than 1 out of 6 patients in a cohort experiences a DLT during the first treatment course (Cycle 1) i.e. the incidence of dose-limiting toxicity is no more than 17% (i.e. 1/6 patients).

The safety evaluation of the dose level 2: continuous administration of nintedanib at 200 mg b.i.d except the days of docetaxel infusion will be followed by the tolerance evaluation of the dose level 3: continuous administration of nintedanib 200 mg b.i.d. This evaluation will be done following the same rules i.e. number of DLT during the first cycle.

However, all DLTs occurring in the trial will be considered for selection of a dose for further development of nintedanib in combination with weekly docetaxel.

For the purpose of escalation, only DLT events that occur during the first treatment cycle of 28 days will be considered. However decisions regarding dose escalation steps will be made only after discussion between the sponsor and the clinical investigators at all sites, and in consideration of the available toxicity. The pharmacokinetic analysis will be done only when nintedanib administration will be done continuously without interruption during docetaxel infusions (dose level 3).

With the agreement of the clinical monitor and the coordinating investigator, patients with a Clinical Benefit may continue to receive treatment for as long as there is a Clinical Benefit. Clinical Benefit can include significant symptom control of originally symptomatic tumours and can be considered even a radiological progression exist. The date of progressive disease will always be documented.

DLT events in individual patients occurring during the first treatment course have to be reported immediately to the Sponsor Clinical Monitor through a DLT form (see Section 5.3.6).

5.1.2 Secondary Endpoint(s)

There are no formal secondary endpoints.

5.1.3 Further Endpoint(s)

5.1.3.1 Pharmacokinetics

The pharmacokinetics of nintedanib and its two metabolites (BIBF 1202 and BIBF 1202 glucuronide) will also be assessed for dose level 3 only. PK parameters evaluated are summarized in section 5.4.1.

5.1.3.2 Endpoint(s) of efficacy

There is no primary or secondary endpoint for efficacy.

Further efficacy endpoints are as follows:

- Objective response (OR) is defined as best overall response of complete response (CR) or partial response (PR) where best overall response is determined according to Response Evaluation Criteria In Solid Tumours (RECIST) version 1.1 recorded from date of first treatment administration until the earliest of disease progression, death or last adequate tumour assessment before new anti-cancer therapy.

  Objective response will be analysed in terms of objective response rate (ORR), defined as the proportion of patients with complete response (CR) or partial response (PR).

  The objective response (i.e. complete response (CR) or partial response (PR)) will be confirmed by imaging 4 weeks or later after the first occurrence of the response.

- Duration of objective response is defined as the time from first documented complete response (CR) or partial response (PR) until the earliest of disease progression or death.

- Progression free survival (PFS) is defined as the time from first treatment administration until tumour progression according to RECIST 1.1 or death from any cause, whichever occurs earlier. Patients who stopped active treatment will be followed-up as described in Section 6.2.3.

5.2 ASSESSMENT OF EFFICACY

5.2.1 Assessment of efficacy following RECIST criteria (version 1.1) (Appendix 10.3).

One to 5 target lesions (up to a maximum of 2 per organ) should be identified at baseline and recorded in the electronic case report form (eCRF) by the investigator. Lesions in areas irradiated within the past 3 months are not considered measurable at baseline. However, new lesions occurring in previously irradiated areas have to be considered for assessment of tumour response. At baseline a full examination has to be done including chest, abdominal, and pelvic CT or MRI to fully assess the extent of the tumour and metastases in an individual patient. MRI/CT of other body parts including brain should be performed as indicated by clinical needs.

The baseline scan must be performed within 28 days prior to administration of first dose of docetaxel. Imaging will be performed at Day 1 of every odd cycle after first administration of docetaxel (within 7 days). Imaging can be performed outside of scheduled visit if progression is suspected. The same method of assessment and the same technique should be used to characterise each reported lesion at baseline and during follow-up (except for brain metastases where CT and/or MRI can be used). Patients with stable brain metastases (for definition please see the exclusion criteria in Section 3.3.3) at trial entry have to be evaluated at each imaging time point for assessment of their brain metastases. Bone scans at baseline need to be performed in patients with known bone metastases and in case of clinical suspicion of previously unknown bone metastasis (i.e. bone or joint pain associated with relevant increases of calcium and alkaline phosphatase [ALP]). In case of a positive bone scan a correlative conventional image should be done of the respective lesion(s). These images should be performed at every imaging time point. Bone scans need to be repeated only at the time when response confirmation imaging is done or when medically indicated i.e., in case of suspected new bone metastases.

Tumour assessment will be evaluated according to the RECIST 1.1 [R09-0262] (Appendix 10.3)

The investigators will assess the tumour images during the trial at the time points specified. Their assessment will be the basis for continuation or discontinuation of treatment in an individual patient (see Section 3.3.4.1).

5.2.2 Progression of disease

Date and reason of progressive disease (PD) based upon RECIST 1.1 imaging evaluation or upon clinical evaluation will be recorded in the eCRF.

5.3 ASSESSMENT OF SAFETY

The safety evaluation is based on the occurrence of DLTs, overall safety and on safety laboratory parameters and will be done following CTCAE version 4.0.

5.3.1 Physical examination

A general physical examination will be performed at screening and at the time points specified in the Flow chart. Whenever possible, the same investigator should perform this examination.

Measurement of height (in cm), body weight (in kg) and the evaluation of the ECOG performance status (PS) will be performed at the time points specified in the Flow chart, which should be recorded with date in the eCRF.

5.3.2 Vital Signs

Vital signs will be performed at screening and at the time points specified in the Flow chart.

5.3.3 Safety laboratory parameters

Blood and urine samples must be collected at the time points specified in the Flow chart. Safety laboratory examinations include haematology, biochemistry, coagulation and urinalysis, which should be recorded with date and time of taken blood sample in the eCRF. In the event of neutrophil count decreased or white blood cell count (WBC) decreased of CTCAE grade 4, blood has to be collected at intervals deemed necessary by the investigator until recovery to a CTCAE grade level that would allow further therapy.

The following parameters will be investigated:

Haematology

Haemoglobin, white blood cells count (WBC), absolute neutrophil count (ANC), and platelets.

Biochemistry

Glucose, sodium, potassium, calcium, phosphorus, magnesium, serum creatinine, clearance creatinine, aspartate amino transferase (AST), alanine amino transferase (ALT), alkaline phosphatase (ALP),γ-GT, lactate dehydrogenase, albumin, , total bilirubin (in case of bilirubin increase to CTCAE Grade 1 or higher, the direct and indirect bilirubin levels have to be determined), urea , total protein, uric acid.

Coagulation parameters

Prothrombin time (PT) and international normalised ratio (INR) or activated partial thromboplastin time (aPTT).

Urine

Proteins, pH, glucose, erythrocytes, leukocytes, nitrite (analysed by quantitative or semi quantitative [dipstick] measurement)
In case of pathological findings, further evaluation should be performed and the findings documented. Urine dipstick for patients with proteinuria ≥2+ at the screening, a 24-hour urine collection for total protein will be obtained within 72 hours to verify the grade of proteinuria for judgment of exclusion criteria.

If laboratory investigations have been performed within 7 days prior to registration, the results may be used for screening. During the treatment period, if the lab investigations have been performed within 2 days prior to a visit and complete results are available, they do not have to be repeated. However, the reference ranges must be provided to the sponsor in case a different laboratory was used.

A laboratory abnormality may meet the criteria to qualify as an AE as described in this protocol. In these instances, the AE corresponding to the laboratory abnormality will be recorded in the eCRF.

5.3.4 Electrocardiogram

A 12-lead resting ECG will be performed at the time points specified in the Flow chart. If the ECG has been performed within 2 days prior to Day 1 of every odd number of treatment cycles and complete results are available, they do not have to be repeated. The investigator should review the ECG during the study and any relevant findings will be reported as AE in the eCRF.

5.3.5 Other safety parameters:

Height, weight and ECOG performance score, pregnancy test will be performed at the time points specified in the Flow chart.

5.3.6 Assessment of adverse events

Adverse event

An adverse event (AE) is defined as any untoward medical occurrence in a patient or clinical investigation subject administered a medicinal product and which does not necessarily have to have a causal relationship with this treatment.

An AE can therefore be any unfavourable and unintended sign (including an abnormal laboratory finding), symptom, or disease temporally associated with the use of a medicinal product, whether or not considered related to the medicinal product.

Dose Limiting Toxicity (DLT)

Only DLTs which occur during the first cycle will be taken into consideration for the determination of the MTD.

DLT is defined as any of the following AEs considered to be related to nintedanib:

1. Drug related CTCAE grade 3 or greater non haematological drug related AEs.
2. Drug related CTCAE grade 2 diarrhoea for more than 7 days despite supportive care.
3. Drug related CTCAE grade 3 or greater nausea despite supportive care.
4. Drug related CTCAE grade 2 or greater vomiting despite supportive care.
5. A drug related increase of ALT and/or AST to CTCAE grade 3 or greater.
6. A drug related increase of ALT and/or AST to CTCAE grade 2 or greater in conjunction with CTCAE grade 1 or greater total bilirubin increase.
7. Platelets < 50000 / mm3 with bleeding (Drug related CTCAE ≥ 3).
8. Drug related Neutropenia of any grade/duration accompanied by fever > 38°5C.
9. Drug related Neutropenia grade 4 without fever > 7 days duration.
10. Inability to resume nintedanib dosing within 21 days after stopping due to drug related toxicity.

The DLT occurring during cycle 1 has to be reported immediately to the Sponsor Clinical Monitor.

All AEs fulfilling the definition of DLT listed above, but occurring after cycle 1, will be termed DLT-Like events and there is no need to report them immediately to the Sponsor Clinical Monitor.

Serious adverse event

A serious adverse event (SAE) is defined as any AE which:

- results in death,
- is life-threatening,
- requires inpatient hospitalisation or prolongation of existing hospitalisation,
- results in persistent or significant disability or incapacity,
- is a congenital anomaly/birth defect,
  or

- is to be deemed serious for any other reason if it is an important medical event when based upon appropriate medical judgment which may jeopardize the patient and may require medical or surgical intervention to prevent one of the other outcomes listed in the above definitions.

Life-threatening in this context refers to an event in which the patient was at risk of death at the time of the event; it does not refer to an event that hypothetically might have caused death if more severe.

If cancer is the indication for treatment, only cancers of new histology and cases where there is clear evidence of exacerbation of an existing cancer qualify as a serious event.
Every new cancer occurrence must be reported as a SAE regardless of the duration of time between the drug discontinuation and the occurrence of the cancer. If progressive disease occurs and is associated with symptoms or meets one of the seriousness criteria, the verbatim "Progressive disease" should not be reported, , instead the signs and symptoms of progressive disease will be reported as an adverse event or a serious AE (if applicable).

AEs considered "Always Serious"

In accordance with the European Medicines Agency initiative on Important Medical Events, Boehringer Ingelheim has set up a list of AEs, which by their nature, can always be considered to be "serious" even though they may not have met the criteria of an SAE as given above.

The latest list of "Always Serious AEs" can be found in the RDC system. These events should always be reported as SAEs as described in Section 5.3.7.

Adverse events of special interest (AESIs)

The term AESI relates to any specific AE that has been identified at the project level as being of particular concern for prospective safety monitoring and safety assessment within this trial, e.g. the potential for AEs based on knowledge from other compounds in the same class. AESI need to be reported to the Sponsor's Pharmacovigilance Department within the same timeframe that applies to SAE, see Section 5.3.7.

The following are considered as AESIs:

Drug-induced liver injury (Appendix 10.1)

Drug-induced liver injury is under constant surveillance by the sponsor and regulators and is considered an adverse event of special interest based on the following definitions. The timely detection, evaluation, and follow-up of laboratory alterations of selected liver laboratory parameters to distinguish an effect of the investigational drug from other causes are important for patient's safety and for the medical and scientific interpretation of the finding.

The following changes in the laboratory values are considered to be an adverse event of special interest for all patients:

- an elevation of ALT and/or AST > 5x ULN without bilirubin elevation measured in the same blood draw sample.
- an elevation of AST and/or ALT >2.5 fold ULN combined with an elevation of bilirubin to >1.5 fold ULN measured in the same blood draw sample.

These lab findings constitute a drug-induced liver injury alert and the patients showing these lab abnormalities need to be followed according to the "DILI checklist" provided in the ISF. In case of clinical symptoms of hepatic injury (icterus, unexplained encephalopathy, unexplained coagulopathy, right upper quadrant abdominal pain, etc.) without lab results (AST, ALT, total bilirubin) available, the investigator should make sure these parameters are analysed, if necessary in an unscheduled blood test. Should the results meet the criteria of hepatic injury alert, the procedures described in the DILI checklist should be followed.

<u>In addition, the following need to be reported to BI as AESIs</u>:

Any gastrointestinal and non-gastrointestinal perforation, leakage, fistula formation, abscess In such case the following additional information needs to be collected, documented in the respective comment field of the eCRF page and forwarded to BI:

- Location of perforation, leakage, fistula, abscess.
- Location/extent of abdominal tumour manifestations.
- Imaging & reports (CT, ultrasound, endoscopy, pathology, etc).
- Prior surgery (location, wound healing complications).
- Concomitant diseases with gastrointestinal (GI) involvement (e.g. Crohn's disease, vasculitis, tuberculosis, diverticulitis).
- Thromboembolic events (or predisposition).

AESIs can be classified as serious or non-serious, but all AESIs must be reported in an expedited manner similar to SAE on an SAE form (i.e., non-serious and serious AESIs must be reported on the SAE form and follow serious timelines) – for details see Section 5.3.7

The intensity of adverse events should be classified and recorded in the eCRF according to the Common Terminology Criteria for Adverse Events (CTCAE) version 4.0.

Causal relationship of AEs

Medical judgment should be used to determine the relationship, considering all relevant factors, including pattern of reaction, temporal relationship, de-challenge or re-challenge, confounding factors such as concomitant medication, concomitant diseases and relevant history.

Yes: There is a reasonable causal relationship between the study medication administered and the AE.

No: There is no reasonable causal relationship between the study medication administered and the AE.

5.3.7 Adverse event collection and reporting

AE collection:

The following must be collected and documented on the appropriate eCRF by the investigator:

From signing the informed consent onwards through the Residual Effect period, until individual patient's end of trial:

- All AEs (non-serious and serious) and all AESIs.

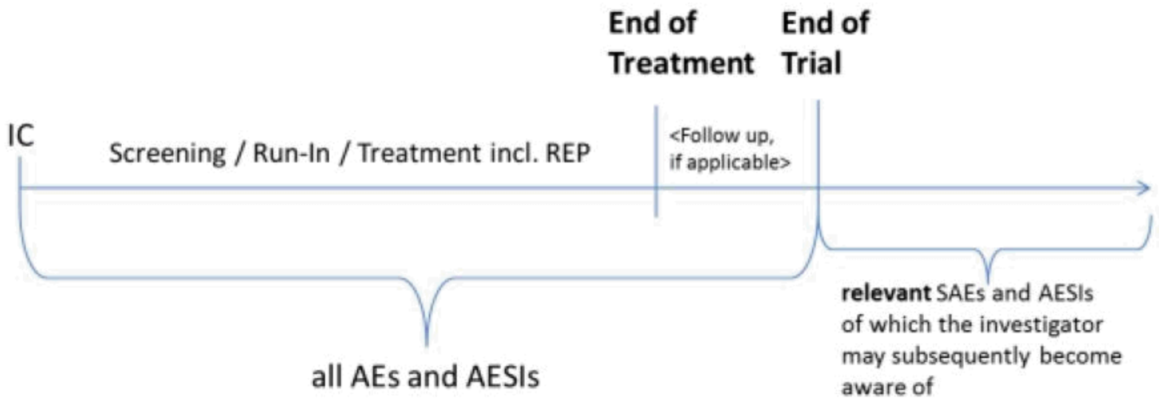

Figure 5.3.7: 1 AE Collection

After the individual patient's end of trial:

- The investigator does not need to actively monitor the patient for AEs but should only report relevant SAEs and relevant AESIs and considered as related to the study drug of which the investigator may become aware of.

Residual effect period (REP)

The residual effect period (REP) is defined as 28 days after the last trial medication application. All AEs which occurred through the treatment phase and throughout the REP will be considered as on treatment. Events which occurred after the REP will be considered as post treatment events.
The EOR is the visit which will be performed at the end of REP, 28 days after permanent discontinuation of trial medication.

AE reporting to sponsor and timelines

The Investigator must report SAEs, AESIs, and non-serious AEs which are relevant for the reported SAE or AESI, on the BI SAE form via fax immediately (within 24 hours) to the Sponsor's unique entry point (country specific contact details will be provided in the ISF).

The same timeline applies if follow-up information becomes available. In specific occasions the Investigator could inform the Sponsor upfront via telephone. This does not replace the requirement to complete and fax the BI SAE form.

Information required

For each AE, the Investigator should provide the information requested on the appropriate eCRF pages and the BI SAE form, e.g. onset, end date, intensity, treatment required, outcome, seriousness, and action taken with the investigational drug(s).
The Investigator should determine the causal relationship to the trial medication.

The following should also be recorded as an (S)AE in the eCRF and SAE form (if applicable):

- Worsening of the underlying disease or of other pre-existing conditions
- Changes in vital signs, ECG, physical examination and laboratory test results, if they are judged clinically relevant by the Investigator.

If such abnormalities already pre-exist prior trial inclusion they will be considered as baseline conditions.

All (S)AEs, including those persisting after individual patient's end of trial must be followed up until they have resolved, have been sufficiently characterized, or no further information can be obtained.

Pregnancy

In rare cases, that a female subject participating in this clinical trial becomes pregnant after having taken trial medication, the investigator must report immediately (within 24 hours) the drug exposure during pregnancy (DEDP) to the Sponsor's unique entry point (country specific contact details will be provided in the ISF). The Pregnancy Monitoring Form for Clinical Trials (Part A) should be used.

The outcome of the pregnancy associated with the drug exposure during pregnancy must be followed up and reported to the Sponsor's unique entry point on the Pregnancy Monitoring Form for Clinical Trials (Part B).

As pregnancy itself is not to be reported as an AE, in the absence of an accompanying SAE, only the Pregnancy Monitoring Form for Clinical Trials and not the SAE form is to be completed. If there is an SAE associated with the pregnancy then the SAE has to be reported on the SAE form in addition.

The ISF will contain the Pregnancy Monitoring Form for Clinical Trials (Part A and B).

Exemptions to SAE Reporting: see Section 5.3.6.

5.4 DRUG CONCENTRATION MEASUREMENTS AND PHARMACOKINETICS

Plasma concentration monitoring of nintedanib (BIBF 1120) and its two main metabolites, BIBF 1202 and BIBF 1202-glucuronide will be performed in order to assess drug exposure in the patients. The obtained predose concentrations will be listed and compared for steady state evaluation.

A detailed PK sampling schedule is provided in section 10.2.2

5.4.1 Assessment of Pharmacokinetics

Nintedanib and its metabolites

$C_{pre,ss}$ (predose concentration of the analyte in plasma at steady state immediately before administration of the next dose)

5.4.2 Methods of sample collection

A detailed description of sample collection and handling is provided in the ISF/ lab manual. For quantification of drug plasma concentrations of nintedanib (BIBF 1120) and its metabolites BIBF 1202 and BIBF 1202- glucuronide, venous blood will be collected using a pre-labeled potassium ethylenediamine-tetraacetic acid (EDTA) containing blood drawing tube.

Date and exact clock time of drug administration and blood sampling on PK sampling days must be recorded on the eCRF.

5.4.3 Analytical determinations

Nintedanib (in form of its free base BIBF 1120 BS), its metabolites BIBF 1202 (in form of the free zwitter ion BIBF 1202 ZW), and the acylglucuronid thereof (BIBF 1202 glucuronide), plasma concentrations will be determined by a validated assay based on liquid chromatography- tandem mass spectrometry (LC-MS/MS) assay.

The procedures and specifications of the analytical method are available at the bioanalytical site (Nuvisan GmbH & Co KG, Wegenerstraße 13, 89231 Neu-Ulm, Germany).

5.4.4 Pharmacokinetic – Pharmacodynamic Relationship:

Not applicable

5.5 ASSESSMENT OF EXPLORATORY BIOMARKERS

Not applicable

5.5.1 Biobanking

Not applicable

5.6 OTHER ASSESSMENTS

Not applicable

5.7 APPROPRIATENESS OF MEASUREMENTS

The RECIST 1.1 to be used for evaluation of tumour response are well established and scientifically accepted (see Appendix 10.3). The CTCAE version 4.0 is used in the assessment of AEs in cancer patients.

6. INVESTIGATIONAL PLAN

6.1 VISIT SCHEDULE

Patients will visit the investigator at the time points specified in the Flow chart. In case, a patient missed a visit and the patient reports to the investigator between this and the next scheduled visit, the missed visit should be done with the actual date. The next visit, however, should take place at the scheduled time after the first administration of the trial drug in the respective treatment cycle.

6.2 DETAILS OF TRIAL PROCEDURES AT SELECTED VISITS

The investigations as outlined in the Flow chart will be performed at the respective visits as described in detail in the following sections.

6.2.1 Screening and treatment period(s)

Screening Period
The screening phase (screening visit) is the phase after informed consent and before the first administration of the trial drug (between Day -14 and Day -1). After having obtained the written informed consent, the investigator may perform the procedures required for screening.
The following will be obtained and/or performed before registration.

- Informed consent for study participation.
- Demographics (sex, birth year, oncological history, smoking status, alcohol consumption, previous therapy, concomitant therapy, baseline condition).
- Medical history.
- Physical examination including resting electrocardiogram (ECG), body height, weight, vital signs and ECOG performance status (PS).
- Tumour staging and assessment: including baseline imaging (e.g. CT or MRI no repeat imaging requested if imaging already done within 28 days prior to Cycle 1).
- Occurrence of adverse events (AEs) since informed consent.
- Concomitant therapy.
- Safety laboratory (no repeat examination in case a safety laboratory investigation has been performed within 7 days prior to registration), including haematology, biochemistry, coagulation parameters and urinalysis.
- Pregnancy test in women of childbearing potential.
- Patient eligibility (inclusion and exclusion criteria).
- Assignment of treatment after reviewing in- and exclusion criteria and registration would be done following Remote Data Capture. As there are no pre-defined cohorts, the IRT system can assign nintedanib and docetaxel kit numbers. For the nintedanib, the site would enter the dose that is needed and the IRT system would allocate a kit number.

- Patients who failed screening may repeat the screening after discussion between investigator and sponsor providing that reasons for screening failure have resolved.

6.2.2 Treatment cycles

For the cohorts of the patients of level-1, 1 and 2: continuous administration with nintedanib except the days of docetaxel administration.

For the cohort of patients of level 3: continuous treatment with nintedanib 200 mg b.i.d without interruption during docetaxel infusion.

6.2.2.1 Cycle 1 Day 1 (C1D1)

The following will be obtained and/or performed before administration of docetaxel:

- The patients may be hospitalized (outpatient hospital) at Day 1 to receive docetaxel infusion after premedication. The premedication is as follows: Oral corticosteroid, such as dexamethasone (8mg twice daily orally the day before and after docetaxel infusion; 8 mg intravenously 30 minutes before docetaxel) and antiemetics before docetaxel infusion (Section 4.1.1).
- Occurrence of AEs since last visit
- Changes in concomitant therapies
- Physical examination, vital signs (pre-dose and after docetaxel infusion) and ECOG
- Body weight
- Resting ECG (may be performed within 2 days prior to the administration of docetaxel)
- Safety laboratory: haematology, biochemistry, coagulation including urinalysis (may be performed within 2 days prior to the administration of docetaxel)
- Pregnancy test (in females of childbearing potential). If the test was already performed at screening within 72 hours prior to docetaxel administration it may be skipped

- Nintedanib dispensation and treatment: :
  - For the cohort of the patients of level -1, level 1 and level 2: treatment of nintedanib continuously except the days of docetaxel infusion. The patients will begin nintedanib treatment at Day 2.
  - For the cohort of patients of level 3: continuous treatment with nintedanib 200 mg b.i.d without interruption during docetaxel infusion. The patients will take the morning dose of nintedanib at hospital after docetaxel infusion.

6.2.2.2 Cycle 1 Day 8 (C1D8) and Cycle 1 Day 15 (C1D15)

- Eligibility of the patients to receive nintedanib treatment and docetaxel infusion (sections 4.1.4.1 – 4.1.4.2).
  - In case retreatment criteria of docetaxel are not met, the administration of docetaxel will be not done. If omission of two consecutive docetaxel infusions occurs, the

continuation of docetaxel should be discussed between the investigator and the sponsor.

- If retreatment criteria are met, the patients will be hospitalized (outpatient hospital) at Day 8 and at Day 15 to receive docetaxel infusion after premedication. Premedication is detailed in Section 4.1.1.
- Nintedanib treatment and PK sampling:
  - For the patients of the cohort level 3: At Cycle 1 Day 8 and at Cycle 1 Day 15, PK blood samples will be withdrawn before nintedanib treatment and before docetaxel infusion. The patients will begin their nintedanib treatment at the hospital after the PK blood sample, during breakfast. The next dose of nintedanib will be taken during the next meal after an interval of 12 hours.
  - For the cohort of the patients of level -1, level 1 and level 2: treatment of nintedanib continuously except the days of docetaxel infusion. The patients will restart nintedanib treatment at Day 9 and at Day 16, respectively.

- Vital signs (pre-dose and after docetaxel infusion) and weight.
- Safety laboratory (haematology and biochemistry) will be obtained before docetaxel infusion.
- AEs occurring during or after docetaxel administration
- Changes in concomitant therapy.

6.2.2.3 Cn Day 1 ± 2 days of every treatment onwards

This CnD1 of the new treatment cycle will be performed on Day 29 (duration of 1 cycle = 28 days, 2 weeks of rest). The last administration of docetaxel was at Day 15 of the previous cycle. A treatment cycle may be postponed for administrative reasons for up to 2 days (e.g. vacation, public holiday).

- Patient eligibility (treatment criteria for docetaxel and for nintedanib, see Sections 4.1.4.):
  - In case retreatment criteria of docetaxel are not met, the administration of docetaxel will be not done. If omission of two consecutive docetaxel infusions occurs, the continuation of docetaxel should be discussed between the investigator and the sponsor.

If retreatment criteria are met, the patients may be hospitalized (outpatient hospital) at Cn Day 1 to receive docetaxel infusion after premedication. At Cycle 2 Day 1: For the cohort of patients of dose level 3 (continuous administration of nintedanib 200 mg b.i.d. without interruption during docetaxel infusion), PK blood samples will be withdrawn before nintedanib treatment and before docetaxel infusion. The patients will begin their nintedanib treatment at the hospital after PK blood sample during breakfast. The next dose of nintedanib will be taken during the next meal after an interval of 12 hours.

The following will be obtained and/or performed before administration of docetaxel.

- Occurrence of AEs since last visit.
- Changes in concomitant therapies.

- Compliance check of nintedanib intake.
- Collection of unused trial medication of nintedanib.
- Physical examination, vital signs (pre-dose and after docetaxel infusion), ECOG PS.
- Body weight.
- Resting ECG (may be performed on Day 1 of every odd number of treatment cycle (3, 5 and so on).within 2 days prior to the administration).
- Safety laboratory including haematology, biochemistry, coagulation parameters and urinalysis (may be performed within 2 days prior to the administration).
- Pregnancy test (in females of childbearing potential) on Day 1 of every odd number of treatment cycle (3, 5, and so on) within 2 days prior to the administration).
- Tumour assessment (CT or MRI) have to be performed on Day 1 of every odd number of treatment cycle (3, 5and so on) until tumour progression. These tumoral assessments could be done within 7 days prior to planned date and outside of scheduled visit if progression is suspected.
- Nintedanib dispensation and treatment:
  - Dispense medication kit nintedanib for the treatment cycle.
  - The patients of the cohorts level -1, level 1 and level 2: continuous administration with interruption during docetaxel infusion. The patients will restart nintedanib treatment at Day 2.
  - The patients of the cohort level 3: continuous treatment without interruption during docetaxel infusion.

6.2.2.4 Cn Day 8 and Cn Day 15 of treatment cycle 2 onwards :

CnDay 8 and Cn Day 15 will be performed at Day 8 ± 2 days and at Day 15± 2 days.

- Patient eligibility (treatment criteria for docetaxel) (see Section 4.1.4.1) :
  - In case retreatment criteria of docetaxel are not met, the administration of docetaxel will be not done. If omission of two consecutive docetaxel infusions occurs, the continuation of docetaxel should be discussed between the investigator and the sponsor.
  - If retreatment criteria are met, the patients may be hospitalized (outpatient hospital) at Day 8 and Day 15 to receive docetaxel infusion after premedication.

At Cycle 2 Day 8 and Day 15: For the cohort of patients of dose level 3 (continuous administration of nintedanib 200 mg b.i.d. without interruption during docetaxel infusion), PK blood samples will be withdrawn before nintedanib treatment and before docetaxel infusion. The patients will begin their nintedanib treatment at the hospital after PK blood sampling, during breakfast. The next dose of nintedanib will be taken during the next meal after an interval of 12 hours.
The following will be obtained and/or performed before administration of docetaxel:

- Occurrence of AEs since last visit.
- Changes in concomitant therapies.
- Vital signs (pre-dose and after docetaxel treatment) and weight.

- Safety laboratory including haematology and biochemistry (may have been performed within 2 days prior to the administration).
- Nintedanib treatment:
  - The patients of the cohorts level -1, level 1 and level 2: continuous administration with interruption during docetaxel infusion. The patients will restart nintedanib treatment at Day 9 and at Day 16 respectively.
  - The patients of the cohort level 3: continuous treatment without interruption during docetaxel infusion.

6.2.2.5 Cn (Day 1) of a treatment cycle with monotherapy nintedanib

Patients who are not eligible for further treatment with docetaxel anymore may be treated with monotherapy of nintedanib if the criteria defined in Section 4.1.4.1.4. are met. Treatment cycles will have duration of 28 days (postponed up to 7 days). Discussion and agreement between investigator and sponsor is mandatory in case retreatment criteria are not met on the scheduled treatment day and a delay for more than 21 days is required.
The following checks will be obtained and/or performed:

- Reason for stopping docetaxel treatment is collected with a specific Trial Termination page at EOT visit.
- Occurrence of AEs since last visit.
- Changes in concomitant therapies.
- Compliance check of nintedanib intake.
- Collection of unused nintedanib.
- Physical examination, vital signs, ECOG PS.
- Body weight and resting ECG (may have been performed within 2 days prior to the administration);
- Safety laboratory: haematology, biochemistry, coagulation parameters including urinalysis (may have been performed within 2 days prior to the administration).
- Pregnancy test (in women of childbearing potential every odd number of treatment cycle within 2 days prior to the administration).
- Patient eligibility for monotherapy/retreatment.
- Tumour assessment (performed on Day 1 of every odd number of treatment cycle).
- Dispense medication kit of nintedanib for the treatment cycle, starting intake on Day 1, plus reserve medication if required (see Section 4.1.1).

6.2.2.6 Optional visits between scheduled visits

Additional unscheduled visits can be performed (on demand) if pathological laboratory findings were observed which have not yet recovered or if close observation of anticoagulant therapy is required or if other issues would require additional visit(s) and/or control. These additional visits will be recorded under the respective scheduled previous visit of an individual treatment cycle in the electronic case report form (eCRF). Differentiation between the various days would be by date.
The following checks will be obtained and/or performed.

- Occurrence of AEs since last visit.
- Changes in concomitant therapies.
- Safety laboratory parameters (haematology, biochemistry, coagulation and urinalysis) of the pathological values only and/or of those which require control.

Other examination and therapy by medical judgement are allowed if required

6.2.3 End of treatment (EOT) and EOR (End of Residual Effect Period (REP)

6.2.3.1 End of treatment

The end of treatment (EOT) visit is to be performed within 7 days after permanent discontinuation of all trial medication (docetaxel and nintedanib).

- Occurrence of AEs since last visit.
- Changes in concomitant therapies.
- Compliance check of nintedanib intake.
- Collection of unused trial medication of nintedanib.
- Physical examination, resting ECG, body weight, vital signs, and ECOG PS.
- Safety laboratory including haematology, biochemistry, coagulation parameters and urinalysis.
- Pregnancy test.
- Tumour assessment and imaging must be performed (If a patient finishes active treatment without having progressive disease unless done within the past 21 days).
- Conclusion of patient's active treatment (including reason for stopping active treatment, date of last administration of the drug[s]).

In case if the patient stops active treatment on scheduled visit, this date should be considered as EOT, and the above investigations should be performed at the visit. If the patient has to stop the active treatment at other than scheduled visit, above investigations should be performed promptly. If the investigator decided to discontinue the trial medication during treatment interruption, examinations that were performed after interruption may be used as data of EOT. Other examinations that were not performed after interruption will be performed at EOT visit as soon as possible.

6.2.3.2 EOR (End of Residual Effect Period (REP)

The EOR visit should be performed at 28 days +7 days after permanent discontinuation of the trial medication. The following will be obtained at the EOR visit:

- Occurrence of AEs since EOT visit and a follow-up of AEs that are not recovered at EOT.
- Any new concomitant therapies or changes in ongoing concomitant therapies
- ECOG PS
- Name and date of any other anti-cancer drug treatment if received

6.2.3.3 Trial completion for an individual patient

A patient has to be considered to have completed the trial in case any of the following applies:

- Completion of planned EOR, and follow-up for AE if required
- Lost to follow up
- Withdrawal of informed consent
- Death

At the earliest of the above criteria, the patient completion information should be entered in the eCRF.

7. STATISTICAL METHODS AND DETERMINATION OF SAMPLE SIZE

7.1 STATISTICAL DESIGN - MODEL

The trial will be performed as an open-label study. The primary objective of the trial is to determine the MTD of nintedanib in combination with weekly docetaxel. To determine the MTD, patients are entered sequentially using the 3+3 design (see Section 3.1). The statistical methods used in this trial will be descriptive.

7.2 NULL AND ALTERNATIVE HYPOTHESES

The analyses in this trial are descriptive and exploratory. No formal statistical tests will be performed.

7.3 PLANNED ANALYSES

No per protocol set will be used in the analysis. However, important protocol violations will be summarised. The TSAP will specify the important protocol violations in detail. For the determination of the MTD, only MTD evaluable patients will be considered. All patients who received at least one dose of study medication (nintedanib or docetaxel) will be included in the analyses of the further endpoints.

7.3.1 Primary endpoint analyses

The primary objective of this study is the tolerability and safety of nintedanib in combination with weekly docetaxel as reflected by the MTD. In order to identify the MTD, the number of patients with DLTs at each dose level must be presented. For the analysis of tolerability and safety, refer to Section 7.3.4.

7.3.2 Secondary endpoint analyses

Not applicable, as there are no secondary endpoints defined.

7.3.3 Further endpoint analyses

Efficacy signals of nintedanib in combination with weekly docetaxel will be explored as further endpoints in this phase I trial. PFS, objective response according to RECIST criteria version 1.1, and duration of objective response will be analysed descriptively for all treated patients.

The PK parameters specified as further endpoints will be analysed according to BI standards (see Section 7.3.5).

7.3.4 Safety analyses

Adverse events will be coded using the Medical Dictionary for Regulatory Activities (MedDRA) coding dictionary. Standard BI summary tables and listings will be produced. All adverse events with an onset between start of treatment and end of the REP will be assigned to the on-treatment period for evaluation.
All treated patients will be included in the safety analysis. In general, safety analyses will be descriptive in nature and will be based on BI standards. No hypothesis testing is planned.

Statistical analysis and reporting of adverse events will concentrate on treatment-emergent adverse events. To this end, all adverse events occurring between start of treatment and end of the residual effect period will be considered ‘treatment-emergent’. Adverse events that start before first drug intake and deteriorate under treatment will also be considered as ‘treatment-emergent’.

Frequency, severity, and causal relationship of adverse events will be tabulated by system organ class and preferred term after coding according to the current version of MedDRA.

Laboratory data will be analysed both quantitatively as well as qualitatively. The latter will be done via comparison of laboratory data to their reference ranges. Values outside the reference range as well as values defined as clinically relevant will be highlighted in the listings. Treatment groups will be compared descriptively with regard to distribution parameters as well as with regard to frequency and percentage of patients with abnormal values or clinically relevant abnormal values.

Vital signs, physical examinations, or other safety-relevant data observed at screening, baseline, during the course of the trial and at the end-of-trial evaluation will be assessed with regard to possible changes compared to findings before start of treatment.

7.3.5 Pharmacokinetic analyses

The pharmacokinetic parameters listed in Section 5.4 will be calculated according to the relevant Corporate Procedure of the Sponsor (MCS-36-472, current version).
Only concentration values within the validated concentration range and actual sampling times will be used for the calculation of pharmacokinetic parameters. Concentrations used in the pharmacokinetic calculations will be in the same format provided in the bioanalytical report, (that is, to the same number of decimal places provided in the bioanalytical report).

Plasma concentrations of nintedanib, BIBF 1202 and BIBF 1202 glucuronide will be presented in tabular and, if feasible, in graphic form for all subjects and sampling points with drug concentrations above the lower limit of quantification. Individual values will be listed and standard summary statistics will be calculated per treatment group for pharmacokinetic parameters.

7.4 INTERIM ANALYSES

No interim analysis is planned.

7.5 HANDLING OF MISSING DATA

Missing baseline laboratory values will be imputed by the respective values from the screening visit.
No other imputations will be performed on missing data although every effort will be made to obtain complete information on all adverse events, with particular emphasis on potential DLTs.

7.5.1 Plasma drug concentration - time profiles

Handling of missing PK data will be performed according to the sponsor's standard operating procedure (MCS-36-472, current version).

Drug concentration data identified with NOS (no sample available), NOR (no valid result), NOA (not analyzed), or BLQ (below the lower limit of quantification) will be displayed as such and not replaced by zero at any time point (this rule also applies to the lag phase, including the pre-dose values).

Descriptive statistics of concentrations at specific time points will be calculated only when at least 2/3 of the individuals have concentrations within the validated concentration range. The overall sample size to decide whether the "2/3 rule" is fulfilled will be based on the total number of samples intended to be drawn for that time point (i.e. BLQ, NOR, NOS, NOA are included).

7.6 RANDOMISATION

No randomisation will be performed. Patients will be assigned to escalating dose groups by order of admission into the trial.

7.7 DETERMINATION OF SAMPLE SIZE

No formal statistical power calculations to determine sample size have been performed. In this open-label phase I trial, up to 24 patients will be allocated to the dosage tiers, in order to find the MTD. Each dose cohort used in this trial will contain at least three individuals. Further 6 patients will be included at dose level 3 and an extension cohort consisting of up to six additional patients will be evaluated. This results in a total number of approximately 30 patients in the trial. However, the number of individuals entered in the trial may vary due to replacement of patients. The simulation study (see statistical appendix) confirms this assumption on patients needed for the dose escalation in this trial.

8. INFORMED CONSENT, DATA PROTECTION, TRIAL RECORDS

The trial will be carried out in accordance with the Medical Devices Directive (93/42/EEC) and the harmonized standards for Medical Devices (ISO 14155-01 and ISO 14155-02).

The trial will be carried out in compliance with the protocol, the ethical principles laid down in the Declaration of Helsinki, in accordance with the ICH Harmonized Tripartite Guideline for Good Clinical Practice (GCP) and relevant BI Standard Operating Procedures (SOPs)*.

Standard medical care (prophylactic, diagnostic and therapeutic procedures) remains in the responsibility of the treating physician of the patient.

The Investigator will inform the Sponsor immediately of any urgent safety measures taken to protect the trial subjects against any immediate hazard, and also of any serious breaches of the protocol or of ICH GCP*.

The rights of the Investigator and of the Sponsor with regard to publication of the results of this trial are described in the Investigator contract. As a rule, no trial results should be published prior to finalization of the Clinical Trial Report.

The certificate of insurance cover is made available to the Investigator and the patients, and is stored in the ISF (Investigator Site File)."

8.1 TRIAL APPROVAL, PATIENT INFORMATION, AND INFORMED CONSENT

This trial will be initiated only after all required legal documentation has been reviewed and approved by the respective Institutional Review Board (IRB) / Independent Ethics Committee (IEC) and competent authority (CA) according to national and international regulations. The same applies for the implementation of changes introduced by amendments.
Prior to patient participation in the trial, written informed consent must be obtained from each patient (or the patient's legally accepted representative) according to ICH / GCP and to the regulatory and legal requirements of the participating country. Each signature must be personally dated by each signatory and the informed consent and any additional patient-information form retained by the Investigator as part of the trial records. A signed copy of the informed consent and any additional patient information must be given to each patient or the patient's legally accepted representative.

The Investigator must give a full explanation to trial patients including the items listed below in association with the use of the patient information form, which is prepared avoiding the use of technical terms and expressions. The patient is given sufficient time to consider participation in the trial. The Investigator obtains written consent of the patient's own free

will with the informed consent form after confirming that the patient understands the contents. The Investigator must sign (or place a seal on) and date the informed consent form. If a trial collaborator has given a supplementary explanation, the trial collaborator also signs (or places a seal on) and dates the informed consent.

8.2 DATA QUALITY ASSURANCE

A quality assurance audit/inspection of this trial may be conducted by the Sponsor, Sponsor's designees, or by IRB / IEC or by regulatory authorities. The quality assurance auditor will have access to all medical records, the Investigator's trial-related files and correspondence, and the informed consent documentation of this clinical trial.

8.3 RECORDS

eCRFfor individual patients will be provided by the Sponsor. For drug accountability, refer to Section 4.1.8.

8.3.1 **Source documents**

Source documents provide evidence for the existence of the patient and substantiate the integrity of the data collected. Source documents are filed at the Investigator's site.
Data reported on the CRF must be consistent with the source data or the discrepancies must be explained. The Investigator may need to request previous medical records or transfer records, depending on the trial; current medical records must also be available.

For the CRF, the following data need to be derived from source documents:
- Patient identification (gender, date of birth).
- Patient participation in the trial (substance, trial number, patient number, date patient was informed).
- Dates of Patient's visits, including dispensing of trial medication.
- Oncological history (advanced solid tumour classification, histology result, stage at screening, location of metastasis sites, EGFR mutation status, ALK mutation status).
- Previous therapies (previous systemic chemotherapies, previous other therapies (immunotherapy, hormone therapy, other), previous radiotherapy, previous surgery).
- Medical history (including trial indication and concomitant diseases, if applicable).
- Adverse events and outcome events (onset date (mandatory), and end date (if available)).
- Serious adverse events (onset date (mandatory), and end date (if available)).
- Concomitant therapy (start date, changes).
- Originals or copies of laboratory results (in validated electronic format, if available).
- Originals or copies of tumour assessments (imaging).
- Completion of Patient's Participation in the trial".
- Prior to allocation of a patient to a treatment into a clinical trial, there must be documented evidence in the source data (e.g. medical records) that the trial participant

meets all inclusion criteria and does not meet any exclusion criteria. The absence of records (either medical records or testing conducted specific for a protocol) to support inclusion/exclusion criteria does not make the patient eligible for the clinical trial.

8.3.2 Direct access to source data and documents

The Investigator / institution will permit trial-related monitoring, audits, IRB / IEC review and regulatory inspection, providing direct access to all related source data / documents. eCRF and all source documents, including progress notes and copies of laboratory and medical test results must be available at all times for review by the Sponsor's clinical trial monitor, auditor and inspection by health authorities (e.g. FDA). The Clinical Research Associate (CRA) / on site monitor and auditor may review all eCRF, and written informed consents. The accuracy of the data will be verified by reviewing the documents described in section 8.3.1.

8.3.3 Storage period of records

Trial site(s):
The trial site(s) must retain the source documents and essential documents for a period defined by the European GCP regulation and trial site's contract with the sponsor.

Sponsor:
The Sponsor must retain the essential documents according to the Sponsor's SOPs.
When it is no longer necessary for the trial site to retain the source documents and essential documents, the Sponsor must notify the head of trial site.

8.4 LISTEDNESS AND EXPEDITED REPORTING OF ADVERSE EVENTS

8.4.1 Listedness

To fulfil the regulatory requirements for expedited safety reporting, the Sponsor evaluates whether a particular adverse event is "listed", i.e. is a known side effect of the drug or not. Therefore, a unique reference document for the evaluation of listedness needs to be provided. For nintedanib this is the current version of the Investigator's Brochure (Number: c 01632700 – 18))-
For the investigational medicinal product, docetaxel, the reference document is the SmPC or another regulatory label document.
The current versions of these reference documents are provided in the ISF.

8.4.2 Expedited reporting to health authorities and IEC / IRB

Expedited reporting of serious adverse events, e.g. suspected unexpected serious adverse reactions (SUSAR) to health authorities and IEC / IRB, will be done according to local regulatory requirements.

8.6 END OF TRIAL

The trial will end when the last patient has completed the EOR visit, 28 days after permanent discontinuation of trial medication and follow up for AE if required.
The IEC / competent authority in each participating EU member state needs to be notified about the end of the trial or early termination of the trial.

8.7 PROTOCOL VIOLATIONS

The investigator should document any deviation from the protocol regardless of their reasons. Only when the protocol was not followed in order to avoid an immediate hazard to trial subjects or for other medically compelling reason, the principal investigator should prepare and submit the records explaining the reasons thereof to the sponsor, and retain a copy of the records.

8.8 COMPENSATION AVAILABLE TO THE PATIENT IN THE EVENT OF TRIAL RELATED INJURY

In the event of health injury associated with this trial, the Sponsor is responsible for compensation based on the contract signed by the trial site.

9. REFERENCES

9.1 PUBLISHED REFERENCES

P08-08684 Hilberg F, Roth GJ, Krssak M, Kautschitsch S, Sommergruber W, Tontsch-Grunt U, et al. BIBF 1120: triple angiokinase inhibitor with sustained receptor blockade and good antitumor efficacy. Cancer Res 2008; 68(12):4774-4782.

P11-08063 Langer CJ, Besse B, Gualberto A, Brambilla E, Soria JC, The evolving role of histology in the management of advanced non-small-cell lung cancer. J Clin Oncol 2010;28(36):5311-5320.

P13-13346 Kaiser R, Barrueco JR, Reck M, Hanna N, Gann C, Glomb P, Gaschler-Markefski B. Identification of a clinical biomarker for 2nd line combination with nintedanib in adenocarcinoma non-small cell lung cancer (NSCLC) patients in two phase III trials. 38th Ann Cong of the European Society for Medical Oncology (ESMO), Amsterdam, 27 Sep - 1 Oct 2013. Eur J Cancer 49 (Suppl 2), S822, Abstr 3479 (2013)

P13-13353 Mellemgaard A, Kaiser R, Douillard JY, Orlov SV, Krzakowski MJ, Pawel J von, Gottfried M, Barrueco JR, Hocke J, Reck M. Analysis of overall survival in adenocarcinoma NSCLC patients receiving 2nd line combination treatment with nintedanib (BIBF 1120) + docetaxel in the LUME-Lung 1 trial: a randomized, double-blind, placebo-controlled phase 3 study. 38th Ann Cong of the European Society for Medical Oncology (ESMO), Amsterdam, 27 Sep - 1 Oct 2013. Eur J Cancer 49 (Suppl 2), S798, Abstr 3409 (2013)

P14-00479 Reck M, Kaiser R, Mellemgaard A, Douillard JY, Orlov S, Krzakowski M, et al. LUME-Lung 1 Study Group Docetaxel plus nintedanib versus docetaxel plus placebo in patients with previously treated non-small-cell lung cancer (LUME-Lung 1): a phase 3, double-blind, randomised controlled trial. Lancet Oncol 2014; 15(2):143-155.

R04-2230 Taxotere (docetaxel), injection concentrate, Rx only (prescribing information as of May 2004). 2004.

R04-4507 Lynch TJ, Bell DW, Sordella R, Gurubhagavatula S, Okimoto RA, Brannigan BW, et al. Activating mutations in the epidermal growth factor receptor underlying responsiveness of non-small-cell lung cancer to gefitinib. N Engl J Med 2004;350(21):2129-2139.

R06-0986 Antiemetic Subcommittee of the Multinational Association of Supportive Care in Cancer (MASCC) Prevention of chemotherapy- and radiotherapy-induced emesis: results of the 2004 Perugia International Antiemetic Consensus Conference. Ann Oncol 17 (1), 20 - 28 (2006)

R07-0220 Handbook of statistics in clinical oncology. Crowley J, Ankerst DP, Second Edition, Boca Raton, Chapman & Hall/CRC Press, 2006 (2006)

R07-1161 Sandler A, Gray R, Perry MC, Brahmer J, Schiller JH, Dowlati A, et al. Paclitaxel-carboplatin alone or with bevacizumab for non-small-cell lung cancer. N Engl J Med 2006;355(24):2542-2550.

R07-1199 Thatcher N, Chang A, Parikh P, Rodrigues Pereira J, Ciuleanu T, Pawel J von, et al. Gefitinib plus best supportive care in previously treated patients with refractory advanced non-small-cell lung cancer: results from a randomised, placebo-controlled, multicentre study (Iressa Survival Evaluation in Lung Cancer). Lancet 2005;366:1527-1537.

R07-4086 Taxotere 20 mg concentrate and solvent for solution for infusion (23/10/2006). 2006.

R08-2275 Gridelli C, Gallo C, Maio M di, Barletta E, Illiano A, Maione P, Salvagni S, Piantedosi FV, Palazzolo G, Caffo O, Ceribelli A, Falcone A, Mazzanti P, Brancaccio L, Capuano MA, Isa L, Barbera S, Perrone F, DISTAL (Docetaxel In Second-line Treatment of Advanced Lung cancer) Investigators. A randomised clinical trial of two docetaxel regimens (weekly vs 3 week) in the second-line treatment of non-small cell lung cancer. The DISTAL 01 study. Br J Cancer 91 (12), 1996 - 2004 (2004)

R08-2280 Camps C, Massuti B, Jimenez A, Maestu I, Garcia Gomez R, Isla D, Gonzalez JL, Almenar D, Blasco A, Rosell R, Carrato A, Vinolas N, Batista N, Garcia Giron C, Galan A, Lopez M, Blanco R, Provencio M, Diz P, Felip E, Spanish Lung Cancer Group. Randomized phase III study of 3-weekly versus weekly doctaxel in pretreated advanced non-small-cell lung cancer: a Spanish Lung Cancer Group trial. Ann Oncol 17 (3), 467 - 472 (2006)

R08-2617 Molina JR, Yang P, Cassivi SD, Schild SE, Adjei AA. Non-small cell lung cancer: epidemiology, risk factors, treatment, and survivorship. Mayo Clin Proc 2008;83(5):584-594.

R09-0262 Eisenhauer EA, Therasse P, Bogaerts J, Schwartz LH, Sargent D, Ford R, et al. New response evaluation criteria in solid tumours: revised RECIST guideline (version 1.1). Eur J Cancer 2009;45:228-247.

R11-0052 Goldstraw P, Crowley J, Chansky K, Giroux DJ, Groome PA, Rami-Porta R, et al. International Association for the Study of Lung Cancer International Staging Committee and Participating Institutions. The IASLC Lung Cancer Staging Project: Proposals for the Revision of the TNM Stage Groupings in the Forthcoming (Seventh) Edition of the TNM Classification of Malignant Tumours. J Thorac Oncol 2007;2(8):706-714.

R11-1327 Weiss J, et al. Frequent and focal FGFR1 amplification associates with therapeutically tractable FGFR1 dependency in squamous cell lung cancer. Sci Transl Med 2010:2(62); 62ra93.

R12-2552 Hanahan D, Weinberg RA, Hallmarks of cancer: the next generation. Cell 144 (5), 646 - 674 (2011)

R13-0232 Shaw AT, Kim DW, Nakagawa K, Seto T, Crino L, Ahn M, et al. Phase III study of crizotinib versus pemetrexed or docetaxel chemotherapy in patients with advanced ALK-positive non-small cell lung cancer (NSCLC) (PROFILE 1007). 37th Ann Cong of the European Society for Medical Oncology (ESMO), Vienna, 28 Sep - 2 Oct 2012 Ann Oncol 23 (Suppl 9), IXe21, Abstr LBA1_PR (2012).

R13-3173 Ellis LM, Bernstein D, Voest E, Berlin J, Sargent D, Cortazar P, Garrett-Mayer E, Herbst R, Lillienbaum R, Sima C, Venook A, Gonen M, Schnipper L, ASCO Clinically Meaningful Outcomes Working Groups. Defining clinically meaningful outcomes: ASCO recommendations to raise the bar for clinical trials (draft for public comment). http://www.asco.org/sites/www.asco.org/files/asco_meaningful_outcomes_4.23.12_draft_for_comment_april_20131.pdf (access date: 8 July 2013) (2013)

R13-3128 Ferrara N. Pathways mediating VEGF-independent tumor angiogenesis. Cytokine Growth Factor Rev 2010;21(1):21-26.

R13-4194 Mercurio AM, Bachelder RE, Bates RC, Chung J. Autocrine signaling in carcinoma: VEGF and the alpha6beta4 integrin. Semin Cancer Biol 2004;14: 115-122.

R13-4197 Weigand M, Hantel P, Kreienberg R, Waltenberger J. Autocrine vascular endothelial growth factor signalling in breast cancer. Evidence from cell lines and primary breast cancer cultures in vitro. Angiogenesis 2005;8:197-204.

R13-4198 Yang F, Tang X, Riquelme E, Behrens C, Nilsson MB, Giri U, et al. Increased VEGFR-2 gene copy is associated with chemoresistance and shorter survival in patients with non-small-cell lung carcinoma who receive adjuvant chemotherapy. Cancer Res 2011;7(16):5512-5521.

R14-2236 Ferlay J, Steliarova-Foucher E, Lortet-Tieulent J, Rosso S, Coebergh JWW, Comber H, et al. Cancer incidence and mortality patterns in Europe: estimates for 40 countries in 2012. Eur J Cancer 2013;49:1374-1403.

R15-1184 Schuette W, Nagel S, Blankenburg T, Lautenschlaeger C, Hans K, Schmidt EW, Dittrich I, Schweisfurth H, Weikersthal LF von, Raghavachar A, Reissig A, Serke M. Phase III study of second-line chemotherapy for advanced non-small-cell lung cancer with weekly compared with 3-weekly docetaxel. J Clin Oncol 23 (33), 8389 - 8395 (2005)

R15-3057 Paz-Ares L, Horn L, Borghaei H, Spigel DR, Steins M, Ready N, Chow LQM, Vokes EE, Felip E, Holgado E, Barlesi F, Kohlhaeufl M, Rodriguez O, Burgio MA, Fayette J, Gettinger SN, Harbison C, Dorange C, Finckenstein FG, Brahmer JR Phase III, randomized trial (CheckMate 017) of nivolumab (NIVO) versus docetaxel (DOC) in advanced non-squamous cell (non-SQ) non-small cell lung cancer (NSCLC). 51st Ann Mtg of the American Society of Clinical Oncology (ASCO), Chicago, 29 May - 2 Jun 2015 ; J Clin Oncol 33 (Suppl),, Abstr LBA109 (2015)

R15-3058 Spigel DR, et al. A phase III study (CheckMate 017) of nivolumab (NIVO; anti-programmed death-1 [PD-1]) vs docetaxel (DOC) in previously treated advanced or metastatic squamous (SQ) cell non-small cell lung cancer (NSCLC). 51st Ann Mtg of the American Society of Clinical Oncology (ASCO), Chicago, 29 May - 2 Jun 2015 ; J Clin Oncol 33 (Suppl),, Abstr 8009 (2015)

R15-5085 Maio M di, Perrone F, Chiodini P, Gallo C, Camps C, Schuette W, Quoix E, Tsai CM, Gridelli C. Individual patient data meta-analysis of docetaxel administered once every 3 weeks compared with once every week second-line treatment of advanced non small-cell lung cancer.
J Clin Oncol 25 (11), 1377 - 1382 (2007)

9.2 UNPUBLISHED REFERENCES

c01632700-18 Kaiser R;Loembe AB;Morsli N. Investigator's Brochure Nintedanib (BIBF1120) for Cancer. 19 Jan 2015

c03149997 Kretschmar G, Knirsch L, LüdtkeD, KraemerS. Pharmacokinetics, safety and tolerability of nintedanib single oral dose in male and female patients with different degrees of hepatic impairment (Child-Pugh classification A and B) as compared with nintedanib administration to male and female healthy subjects (a non-blinded, parallel group study of Phase I) 20 my 2015

U02-1109 Ulrike T.G.. Analysis of potency and selectivity of the vascular endothelial growth factor receptor-2 inhibitor BIBF1120. in vitro studies. Report/Study No. 01-02, 19-feb-2002.

U02-1482 Kaiser R. Investigator's brochure: nintedanib, Indication: Oncology Project: 1199.P1, 1199.P2, 1199.P4, 1199.P5, 1199.P6, 1199.P7, 1199.P8, 1199.P11, Version 13, 15-Nov-2013.

U08-1273-02 Raymond E, De-Mont-Serrat H, Rouyrre N, Hoesl C, Stopfer P. A phase I open label dose escalation study of continuous (except on the days of chemotherapy infusion) oral treatment with BIBF 1120 together with

docetaxel and prednisone in patients with hormone refractory prostate cancer. BI Trial No. 1199.4. 16 May 2008.

U08-1946 Hilberg F. Effects of BIBF 1120 on MAPK and Akt signaling, cell proliferation and induction of apoptosis in endothelial cells, smooth muscle cells and pericytes in vitro. Document/Study No. BIA09-08, 11-Sep-2008.

U10-1846-01 Konishi K, Kagimura T, Sarashina A, A Phase I open-label dose-escalation study of continuous twice-daily oral treatment with BIBF 1120 in Japanese patients with advanced solid tumours. BI Trial No. 1199.19. 26 June 2009.

U11-1947-01 G Bader. X-ray structure analysis of VEGFR-2 kinase in complex with BIBF 1120. Report/Study No. BIRCV09-11, 26-Jul-2011.

10. APPENDICES

10.1 CLINICAL EVALUATION OF LIVER INJURY

10.1.1 Introduction

Alterations of liver laboratory parameters, as described in Section 5.3.6 (adverse events of special interest [AESI]), are to be further evaluated using the following procedures:

10.1.2 Procedures

ALT and/or AST and total bilirubin increases falling into the categories as defined in Section 5.3.6 have to be confirmed using the initial blood sample if possible (sample still available, no lysis etc). The alkaline phosphatase (ALP) should be measured using the same sample too. In case local laboratories are being used, the laboratory parameters have to be repeated locally. If site receives a potential drug induced liver injury (DILI) alert notice from the local laboratory, the following tasks have to be completed:

1. Repeat the following laboratory tests locally for confirmation within 48 hours:

- AST, ALT, bilirubin measurement (total and direct bilirubin) and ALP.
- Haptoglobin.
- Complete blood count and cell morphology.
- Reticulocyte count.
- Creatine kinase (CK).
- Lactate dehydrogenase (LDH).

The results of these repeated laboratory tests must be documented on the electronic case report form (eCRF) and reported immediately via the serious adverse event (SAE) form to BI.

2. An evaluation of the patient within 48 hours with respect to but not limited to:

- Abdominal ultrasound or clinically appropriate alternate imaging and investigations adequate to rule out biliary tract, pancreatic, intra- or extrahepatic pathology e.g. bile duct stones, neoplasm, hepatic tumour involvement, biliary tract, pancreatic or intrahepatic pathology, vascular hepatic conditions such as portal vein thrombosis or right heart failure. These data need to be collected, documented in the respective field of the eCRF/DILI checklist, and the respective SAE form has to be updated and forwarded to BI.
- The findings from the imaging and investigations (including comparison to prior imaging if available) must be discussed with representatives of the Sponsor via face to face meeting or phone.

For all patients fulfilling protocol specified criteria for liver injury, please collect and document the following information in the eCRF and the SAE form according to the “DILI checklist” provided in the ISF:

- Detailed history of current symptoms, concurrent diagnoses and medical history.
- Detailed history of concomitant drug use (including non-prescription medications, herbal and dietary supplement preparations e.g. steroids as concomitant supportive treatment), alcohol use, recreational drug use, and special diets.
- Detailed history of exposure to environmental chemical agents (consider home and work place exposure).

Please complete the following laboratory tests as detailed in the DILI checklist provided in the ISF in case that both imaging and laboratory value did not unequivocally confirm cholestasis as the reason for ALT/AST increase, in particular if ALP <2x ULN:

- Clinical chemistry

  ALP, cholinesterase (either plasma or red blood cell), albumin, prothrombin time (PT) or international normalised ratio (INR), CK, CK-MB, coeruloplasmin*, α-1 antitrypsin*, transferrin, ferritin, amylase*, lipase*, fasting glucose*, cholesterol, triglycerides

- Serology

  Hepatitis A (Anti-IgM, Anti-IgG), Hepatitis B (HbsAg, Anti-HBs, DNA), Hepatitis C (Anti-HCV, RNA if Anti-HCV positive), Hepatitis D (Anti-IgM, Anti-IgG)*, HDV-RNA* analysis is accepted if Anti-IgM and Anti-IgG are not available at the laboratory)**, Hepatitis E (Anti-HEV, Anti-HEV IgM, RNA if Anti-HEV IgM positive)*, HEV-RNA* analysis is accepted if Anti-HEV and Anti-HEV IgM are not available at the laboratory)**, Anti- Smooth Muscle antibody (titer)*, Anti-nuclear antibody (titer)*, Anti-LKM (liver- kidney microsomes) antibody*, Anti-mitochondrial antibody*, Epstein Barr Virus (VCA IgG, VCA IgM), cytomegalovirus (IgG, IgM), herpes simplex virus (IgG, IgM), varicella (IgG, IgM), parvovirus (IgG, IgM)

- Hormones

  Thyroid-stimulating hormone (TSH)*

- Haematology

  Thrombocytes*, eosinophils*

  * If clinically indicated and in case additional investigations are needed (e.g. immunocompromised patients)

  ** HDV and HEV related analyses have to be performed if possible at the study site or the external laboratory vender which is standard vender for the study site.

Initiate close observation of all patients with elevated liver enzyme and total bilirubin increases as defined in Section 5.3.6 by repeat testing of ALT, AST, bilirubin (with fractionation to total and direct) and ALP at least weekly until the laboratory values return to normal or to the values as defined in the protocol.

In case that transaminases and/or total bilirubin increase despite cessation of the experimental therapy, more frequent intervals will be warranted.
Depending on further laboratory changes, additional parameters identified e.g. by reflex testing will be followed up based on medical judgment and Good Clinical Practices.

10.2 PHARMACOKINETIC ANALYSES

10.2.1 Handling procedure of blood samples for plasma concentration-time measurements

For the calculation of pharmacokinetic parameters, only concentrations within the validated concentration range will be used. The actual sampling times will be used. For pre-dose samples, the actual sampling time will be set to zero. Pharmacokinetic analyses will be done according Corporate Procedure of the Sponsor (SOP 001-MCS-36-472, current version).

10.2.2 Time schedule for Pharmacokinetic (PK) blood sampling

Table 10.2.2: 1 Time schedule for PK blood sampling Cycle 1

Treatment cycle	Day	Time Point [hh:min]	Event	Sample No.	Nintedanib[a)]
Cycle 1	8	Just before docetaxel/nintedanib administration [-0:05]	PK Blood	1	X
	15	Just before docetaxel/nintedanib administration [-0:05]	PK Blood	2	X

[a)]Analytes: BIBF 1120, BIBF 1202, BIBF 1202 glucuronide

Table 10.2.2: 2 Time schedule for PK blood sampling Cycle 2

Treatment cycle	Day	Time Point [hh:min]	Event	Sample No.	Nintedanib [a)]
Cycle 2	1	Just before docetaxel/nintedanib administration [-0:05]	PK Blood	1	X
	8	Just before docetaxel/nintedanib administration [-0:05]	PK Blood	2	X
	15	Just before docetaxel/nintedanib administration [-0:05]	PK Blood	3	X

[a)]Analytes: BIBF 1120, BIBF 1202, BIBF 1202 glucuronide

10.3 RECIST CRITERIA (VERSION 1.1)

The criteria below are based on RECIST 1.1 [R09-0262].

Measurability of the disease

Measurable lesions

Lesions that can be accurately measured in at least one dimension with longest diameter ≥10 mm (by CT scan, MRI, caliper measurement) or ≥20 mm (by chest X-ray).

Measurable disease

Measurable disease requires the presence of at least one measurable lesion. If the measurable disease is restricted to a solitary lesion, its neoplastic nature should be confirmed by cytology/histology.

Non-measurable disease

Non-measurable lesions are all other lesions, including small lesions (longest diameter <10 mm with CT scan, MRI or caliper measurement or <20 mm with chest X-ray or pathological lymph nodes with shortest axis ≥10 and <15 mm) as well as truly non-measurable lesions. Lesions considered truly unmeasurable include leptomeningeal disease, ascites, pleural or pericardial effusion, inflammatory breast disease, lymphangitic involvement of skin or lung, abdominal masses/ abdominal organomegaly identified by physical exam that is not measurable by reproducible imaging techniques.

<u>New lesions in irradiated fields</u>

Previously irradiated lesions should not be used as indicator lesions. However, new lesions occurring in previously irradiated fields can be used to assess the antitumour response.

Methods of measurement

All measurements must be recorded in metric notation, using a ruler or calipers. All baseline evaluations must be performed as close as possible to the treatment start and never more than 4 weeks before the beginning of the treatment. If a lesion is considered too small to measure, a default measurement of 5 mm should be applied. If the lesion is not visible, a default measurement of 0 mm should be applied.

The same method of assessment and the same technique must be used to characterise each identified and reported lesion at baseline and during follow-up.

Clinical lesions will only be considered measurable when they are superficial (e.g., skin nodules, palpable lymph nodes). In the case of skin lesions, documentation by colour photography including a ruler to estimate the size of the lesion is obligatory.

CT and MRI are the best currently available and reproducible methods to measure target lesions selected for response assessment. Conventional CT and MRI should be performed with cuts of 5 mm or less in slice thickness contiguously. Spiral CT should be performed using a 5 mm contiguous reconstruction algorithm. This applies to the chest, abdomen and pelvis.

Ultrasound, endoscopy and laparoscopy should not be used to measure tumour lesions or evaluate tumour response. However, these techniques can be useful to supplement information from other techniques.

Tumour markers alone cannot be used to assess response. If markers are initially above the upper normal limit, they must normalise for a patient to be considered in complete clinical response.

Cytology and histology can be used to differentiate between PR and CR in rare cases (for example, residual lesions in tumour types such as germ cell tumours, where known residual benign tumours can remain).

Baseline Documentation of Target and Non-target Lesions

All measurable lesions up to a maximum of two lesions per organ and five lesions in total, representative of all involved organs should be identified as target lesions and will be recorded, measured (longest diameter = LD) and numbered at baseline. Target lesions should be selected on the basis of their size (lesions with the longest diameter) and their suitability for accurate repetitive measurements (either by imaging techniques or clinically). Lymph nodes must be ≥15mm (short axis) in order to be considered as target lesions.

A sum of the longest diameter (LD) for all target lesions will be calculated and reported as the baseline sum LD. For lymph nodes, the short axis will contribute to the baseline sum. The baseline sum LD will be used as reference to further characterize the objective tumour response of the measurable dimension of the disease (see Table 10.3: 1).

Table 10.3: 1 Evaluation of target lesions

Complete Response (CR)	Disappearance of all target lesions. Any pathological lymph nodes (whether target or non-target) must have reduction in short axis to <10 mm (the sum may not be "0" if there are target nodes)
Partial Response (PR)	At least a 30% decrease in the sum of LD of target lesions taking as reference the baseline sum LD.
Progression (PD)	At least a 20% increase in the sum of LD of target lesions taking as reference the smallest sum LD recorded since the treatment started, together with an absolute increase in the sum of LD of at least 5mm.<br>OR<br>The appearance of one or more new lesions.
Stable Disease (SD)	Neither sufficient shrinkage to qualify for PR, taking as reference the baseline sum LD, nor sufficient increase to qualify for PD taking as reference the smallest sum LD since the treatment started.

All other lesions (or sites of disease) should be identified as non-target lesions and should also be recorded at baseline. Measurements are not required and these lesions should be followed as "present" or "absent" (see Table 10.3: 2).

Table 10.3: 2 Evaluation of non-target lesions and new lesions

Complete Response (CR)	Disappearance of all non-target lesions and normalisation of tumour marker level. All lymph nodes must be non-pathological in size (<10 mm short axis)
Non-CR/Non-PD	Persistence of one or more non-target lesions or/and maintenance of tumour marker level above normal limits.
Progression (PD)	Appearance of one or more new lesions and/or unequivocal progression of existing non-target lesions.<br><br>Although a clear progression of non-target lesions only is exceptional, in such circumstances, the opinion of the treating physician should prevail and the progression status should be confirmed later by the review panel (or study chair).

In some circumstances it may be difficult to distinguish residual disease from normal tissue. When the evaluation of complete response depends upon this determination, it is recommended that the residual lesion be investigated (fine needle aspirate/biopsy) before confirming the complete response status.

Duration of stable disease

In the case of SD, follow-up measurements must have met SD criteria at least once after study entry at a minimum interval of six weeks.

Evaluation of overall response

The overall response is determined via the assessment of target lesions, non-target lesions and the appearance of new lesions. The algorithm for evaluation of overall response in patients with measurable disease at baseline is given in table 10.3: 3. For patients with non-measurable disease at baseline, the algorithm for evaluation of overall response is defined in Table 10.3: 4.

Table 10.3: 3 Algorithm for evaluation of overall response in patients with measurable disease at baseline

Target lesions	Non-target lesions	New lesions	Overall response
CR	CR	No	CR
CR	Non-CR/ Non-PD	No	PR
CR	Not evaluated	No	PR
PR	Non-PD or not evaluated	No	PR
SD	Non-PD or not evaluated	No	SD
Not evaluated	Non-PD	No	NE
PD	Any	Yes or No	PD
Any	PD	Yes or No	PD
Any	Any	Yes	PD

Table 10.3: 4 Algorithm for evaluation of overall response in patients with non-measurable disease at baseline

Non-target lesions	New lesions	Overall response
CR	No	CR
Non-CR/ Non-PD	No	Non-CR/ Non-PD
Not all evaluated	No	NE
Unequivocal PD	Yes or No	PD
Any	Yes	PD

Patients with a global deterioration of health status requiring discontinuation of treatment without objective evidence of disease progression at that time should be reported as "*symptomatic deterioration*". Every effort should be made to document the objective progression even after discontinuation of treatment.

10.4 STATISTICAL APPENDIX

A 3+3 design will be used to define the dose escalation steps in this study. This design is introduced in Section 3.1. The purpose of this statistical appendix is to present performance metrics (operating characteristics) that illustrate the precision of the design in determining the MTD under various dose-toxicity relationships through computer simulations. Table 10.4: 1 describes 5 assumed true dose-toxicity scenarios which were used to assess the operating characteristics of the design. These scenarios reflect a wide range of possible cases as follows:

- Scenario 1: aligned with prior toxicity expectations
- Scenario 2: high-toxicity scenario
- Scenario 3: low-toxicity scenario

Table 10.4: 1 Assumed true dose-toxicity scenarios

Scenario		Dose combination		
		100mg / 35mg/m^2	150mg / 35mg/m^2	200mg / 35mg/m^2
1: Expect	P(DLT)	0.13	0.20	0.32
2: High Tox		0.26	0.35	0.48
3: Low Tox		0.05	0.12	0.19

For each of these scenarios, 1000 trials were simulated. Each cohort consisted of 3 patients and dose escalation complied with the rules described in Section 3.1.
The MTD was considered reached at the highest dose combination where not more than 1 out of 6 patients experienced a DLT. If the MTD could not be reached, because already the first specified dose combination was too toxic, the simulated trial was stopped. In case no DLT has been observed during the simulated trial, the highest dose combination that was specified has been declared as MTD.

It was then assessed how often a dose combination was declared as MTD. Furthermore, the average, minimum and maximum number of patients per trial are reported. Results are shown in Table 10.4: 2.

Table 10.4: 2 Simulated operating characteristics

Scenario	% of trials declaring MTD at dose [combination]			% of stopped trials	# Patients
	100mg / 35mg/m^2	150mg / 35mg/m^2	200mg / 35mg/m^2		Mean (Min-Max)
1	20.1	35.8	32.2	11.9	9.94 (6-18)
2	22.5	25.7	7.6	44.1	10.31 (6-18)
3	11.6	22.8	63.6	2.0	8.79 (6-18)

In scenario 1, which reflects the case that the true dose-toxicity is aligned with the prior expectations regarding toxicity probabilities, dose combination 200mg / 35mg/m^2 has been declared as MTD in approximately the same amount of trials as dose combination 150mg / 35mg/m^2 and almost 12% of the trials stopped because the first specified dose combination was too toxic.
In Scenario 2 (high-toxicity scenario), 44% percent of the simulated trials have been stopped prematurely because of toxicity. Therefore, patients are sufficiently protected from being treated with an overly toxic dose.
Scenario 3 (low-toxicity scenario) shows that with low underlying toxicity probabilities, the 3+3 design escalates in many simulated trials up to the highest specified dose combination and declares this as MTD.
The mean patient numbers range from 8.79 patients (Scenario 3) to 10.31 patients (Scenario 2) and the maximum number of patients was 18. Therefore, the patient numbers are as expected for the 3+3 design.

Overall, by reviewing the metrics presented in Table 10.4: 2, it can be seen that the 3+3 design serves as an adequate design to determine the MTD of nintedanib in combination with weekly docetaxel.

R version 3.0.3 was used for the simulations.

11. DESCRIPTION OF GLOBAL AMENDMENT(S)

"This is the original protocol."

**Number of global amendment**		
**Date of CTP revision**		
**EudraCT number**		
**BI Trial number**		
**BI Investigational Product(s)**		
**Title of protocol**		
**To be implemented only after approval of the IRB / IEC / Competent Authorities**		
**To be implemented immediately in order to eliminate hazard – IRB / IEC / Competent Authority to be notified of change with request for approval**		
**Can be implemented without IRB / IEC / Competent Authority approval as changes involve logistical or administrative aspects only**		
**Section to be changed**		
**Description of change**		
**Rationale for change**		
